# Expressional analysis of disease-relevant signalling-pathways in primary tumours and metastasis of head and neck cancers

**DOI:** 10.1038/s41598-018-25512-7

**Published:** 2018-05-09

**Authors:** Dorothee Goesswein, Negusse Habtemichael, Aslihan Gerhold-Ay, Johanna Mazur, Désirée Wünsch, Shirley K. Knauer, Julian Künzel, Christoph Matthias, Sebastian Strieth, Roland H. Stauber

**Affiliations:** 1 Molecular and Cellular Oncology, ENT/University Hospital of Mainz, Mainz, 55131 Germany; 2 Institute for Medical Biostatistics, Epidemiology and Informatics (IMBEI), University of Mainz Medical Center, Mainz, 55101 Germany; 3 Institute for Molecular Biology, Centre for Medical Biotechnology, University of Duisburg-Essen, Essen, 45117 Germany

## Abstract

Head and neck squamous cell carcinoma (HNSCC) often metastasize to lymph nodes resulting in poor prognosis for patients. Unfortunately, the underlying molecular mechanisms contributing to tumour aggressiveness, recurrences, and metastasis are still not fully understood. However, such knowledge is key to identify biomarkers and drug targets to improve prognosis and treatments. Consequently, we performed genome-wide expression profiling of 15 primary HNSSCs compared to corresponding lymph node metastases and non-malignant tissue of the same patient. Differentially expressed genes were bioinformatically exploited applying stringent filter criteria, allowing the discrimination between normal mucosa, primary tumours, and metastases. Signalling networks involved in invasion contain remodelling of the extracellular matrix, hypoxia-induced transcriptional modulation, and the recruitment of cancer associated fibroblasts, ultimately converging into a broad activation of PI3K/AKT-signalling pathway in lymph node metastasis. Notably, when we compared the diagnostic and prognostic value of sequencing data with our expression analysis significant differences were uncovered concerning the expression of the receptor tyrosine kinases *EGFR* and *ERBB2*, as well as other oncogenic regulators. Particularly, upregulated receptor tyrosine kinase combinations for individual patients varied, implying potential compensatory and resistance mechanisms against specific targeted therapies. Collectively, we here provide unique transcriptional profiles for disease predictions and comprehensively analyse involved signalling pathways in advanced HNSCC.

## Introduction

With over 500,000 new cases per year worldwide, head and neck squamous cell carcinoma (HNSCC) develops from multiple anatomic subsites, including oral cavity, hypopharynx, oropharynx, larynx and nasopharynx^1,2^. Main risk factors include tobacco exposure, together with alcohol use, as well as infection with potentially oncogenic viruses, such as HPV-16, HPV-18, HPV-31, and HPV-33^1,3,4^. Moreover, the increasing (ab)use of so called ‘e-liquids’, as a more ‘healthy’ way of smoking, might need to be considered as an additional risk factor. However, conclusive studies are missing so far. When discovered in early-stage, HNSCC are treated with surgery and/or radiotherapy; for advanced stages treatment is multimodal^1^. Surgery is used when possible, usually combined with adjuvant chemoradiation, or definitive chemoradiation^1^. For recurrent and metastatic diseases, chemotherapy is applied with the option of alternative immunotherapy when chemotherapy is not tolerated^1^. All treatment is indicated mainly by primary site, TNM classification and other pathologic features limited by performance status^1,5^. Cancer therapies are changing gradually, shifting from systemic cytotoxic drugs to individual approaches. Essential for these personalized therapies are diagnostic tests predicting the clinical outcome of certain therapies using the vast knowledge of cancer origins and maintenance obtained by molecular biology^6^.

As the accumulation of mutations in tumours from patients with lymph node metastasis is not increased compared to patients without lymph node involvement^7^, other alterations have been suggested^8^. Expression profiling has been used to distinguish between cancer subtypes and predict disease outcome in different types of cancer^9–13^. Previous studies allowed the categorization of HNSCC tumours into distinct subtypes^14^, discovered a gene expression signature associated with recurrent disease^15^, or suggested genes with diagnostic or prognostic potential^16–23^. Microarray analysis performed on normal oral epithelium compared to HNSCC elucidated the roles of microenvironment remodelling and immune responses in survival of HNSCC^24^. The only prognostic marker for HNSCC is the HPV status of the tumour, and predictive biomarkers to influence treatment selection are completely lacking^1,25^. Although HPV positive tumours differ significantly in their expression profiles, treatment options do not depend on the HPV status of the tumour^1^. Several new therapies targeting growth factor receptors or downstream signalling pathways are currently tested in clinical trials^26^. Without matching companion diagnostics, physicians will soon be challenged in choosing the most suitable therapy for each patient.

In this study, we analysed differentially expressed genes of HNSCC primary tumours compared to lymph node metastases in order to explore new biomarker candidates and involved signalling pathways. In contrast to other studies concentrating only on the analysis of primary tumours, we additionally focused on identifying the changes that lead to the dissemination of the primary tumour to lymph node metastasis. Gene expression microarray technology was applied to a group of 15 HNSCC tumours, their corresponding normal tissue mucosa and lymph node metastases resected by surgery. To facilitate data interpretation, statistical gene-set-enrichment methods and Qiagen’s software Ingenuity (Pathway Analysis, Comparison and Upstream Analysis, Biomarker Discovery) were used to intersect expressed genes with sets of genes associated with particular biological functions or pathways (as reviewed in^27^). We focused on obtaining a global view of differential gene expression in HNSCC compared to normal tissue mucosa, the primary tumour and corresponding lymph node metastases. The identification of characteristic gene expression signatures may potentially be exploited for diagnostic, prognostic or therapeutic purposes and promotes the reliability of the corresponding method.

## Results

### Validation of the gene expression data generated by oligonucleotide chip-arrays

In order to analyse gene expression, RNA from primary tumours (PT), lymph node metastases (M), and corresponding normal tissue mucosa (N) was subjected to microarray analysis using Affymetrix HG-U133A chips. The verification of the obtained data quality by independent experimental approaches is a prerequisite for further analytical approaches in all ‘omics’ technologies. Accordingly, we confirmed our chip-array data by quantitative real-time PCR (qRT-PCR) and reverse transcription PCR (RT-PCR) for selected representative genes and samples. RT-PCR was performed to analyse expression patterns of randomly chosen *CLCA4, FN1, POSTN, KRT24* and *PRR4* in primary HNSCC *vs*. their corresponding normal tissue mucosa (PT*vs*.N, Fig. S1A) and *ARPGAP25*, *FAIM3, RASGRP2, LYPD3, SERPINB4* and *TP73L* in lymph node metastasis *vs*. their corresponding primary HNSCC (M*vs*.PT, Fig. S1B). *FN1, POSTN* and *OLR1* genes were confirmed as up-regulated, *KRT24, KRT4* and *PRR4* as down-regulated in PT*vs*.N. *ARPGAP25*, *FAIM3* and *RASGRP2* genes were up-regulated, whereas *LYPD3, SERPINB4* and *TP73L* were down-regulated in M*vs*.PT. Additionally, quantitative PCR analyses were performed for *ARGHAP25* (Fig. S2), *SERPINB4* (Fig. S3) and *PRR4* (Fig. S4). All results of qRT-PCR analyses were in accordance to the microarray data. In summary, the conducted controls underline the quality and reliability of our obtained GeneChip array data, fulfilling all prerequisites for further bioinformatics exploitation, model building and in depth functional and clinical analysis.

### Identification of significantly upregulated genes in primary tumours and corresponding lymph node metastases

After normalization, we calculated the expression differences over all patients to compare primary tumours to normal mucosa (PT*vs*.N) and lymph node metastasis to primary tumours (M*vs*.PT). By sorting the resulting lists using stringent filter criteria (first by p-value, second by log_2_FC), we obtained four TOP 30 lists for highly upregulated PT*vs*.N (Table S4), downregulated PT*vs*.N (Table S5), upregulated M*vs*.PT (Table S6) and downregulated M*vs*.PT (Table S7).

**Figure 1:**
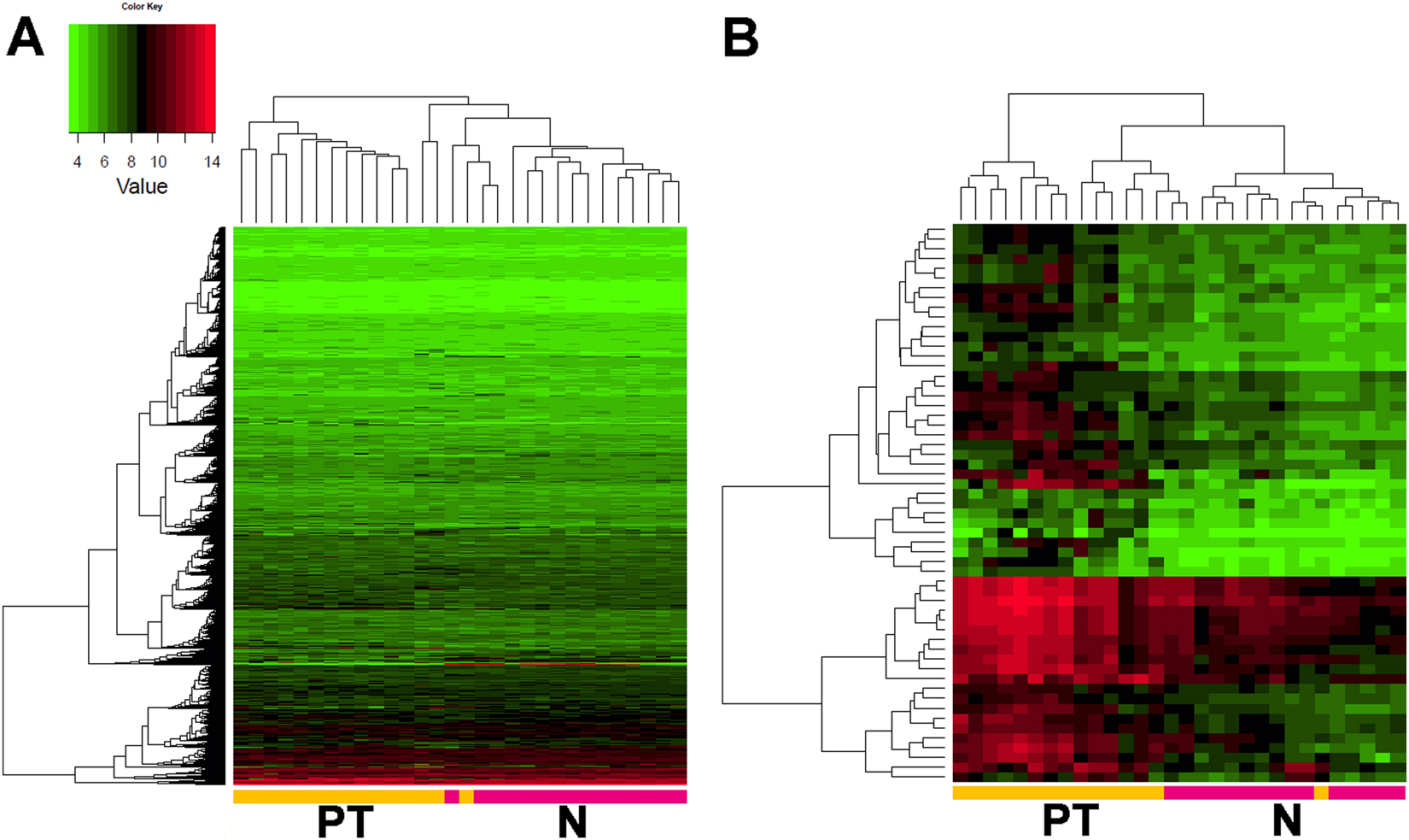
Gene set cluster analysis of 15 HNSCC samples and their 15 corresponding normal mucosa allows profiling of disease progression by identifying a condensed list of 38 most significant genes. Supervised two-way hierarchical clustering and gene tree representation adjusted to the primary site of all genes (A) and differentially regulated genes (B) allow separating primary tumours and normal mucosa. X-axis’ represent patient samples; y-axis’ represent the list of probe sets grouped by similarity using Pearson correlation. Intensities recorded for genes are colour coded: Green – low expression, Red – high expression, Black – medium expression. Orange bar: primary tumour samples; pink bar: normal mucosa samples. (**A**) Heatmap of all probesets shows the comparison of primary tumours and normal tissue mucosa (PT*vs*.N). (**B**) Stringent filter criteria (log_2_FC ≥ 1.5 and p-value < 0.0001, corresponding to a FDR of 0.003) were used to reduce the probesets to the most significant for PT*vs*.N, leading to a condensed list of 38 genes able to distinguish diseased from non-malignant tissue. Signature genes are listed separately in Table S8.

**Figure 2:**
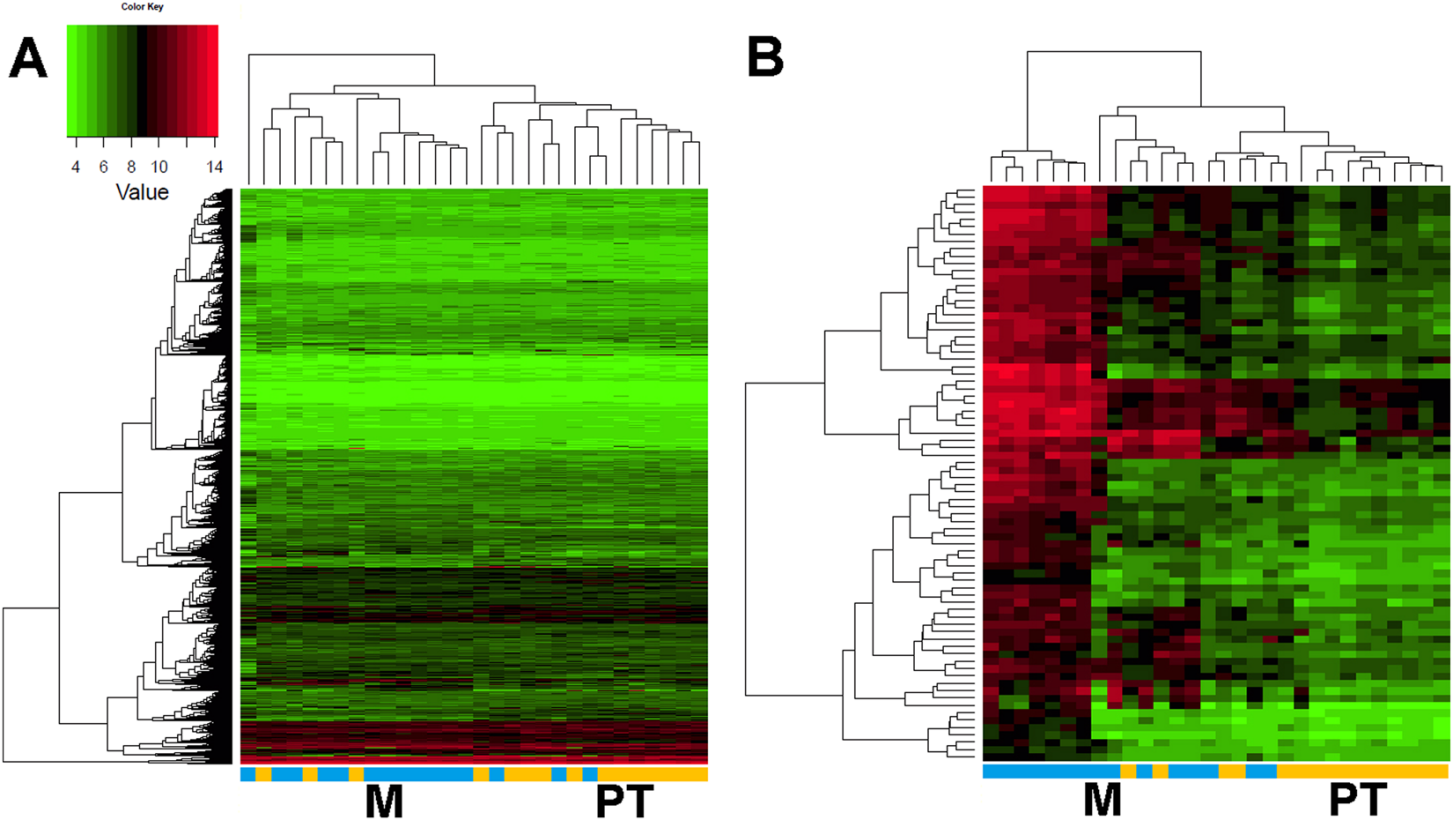
Gene set cluster analysis of 15 lymph node metastasis and their 15 corresponding primary tumours allows profiling of disease progression by identifying a condensed list of 62 most significant genes. Supervised two-way hierarchical clustering and gene tree representation adjusted to the primary site of all genes (A) and differentially regulated genes (B) allow distinguishing lymph node metastasis and primary tumours. X-axis’ represent patient samples; y-axis’ represent the list of probe sets grouped by similarity using Pearson correlation. Intensities recorded for genes are colour coded: Green – low expression, Red – high expression, Black – medium expression. Orange bar: primary tumour samples; blue bar: lymph node metastasis samples. (**A**) Heatmap of all probesets shows the comparison of lymph node metastasis to primary tumours (M*vs*.PT). (**B**) Stringent filter criteria (log_2_FC ≥ 1.5 and p-value < 0.0001, corresponding to a FDR of 0.004) were used to reduce the probesets for M*vs*.PT, leading to a condensed list of 62 genes most significantly changed in primary tumours to their corresponding lymph node metastasis. Signature genes are listed separately in Table S9.

The 45 data sets were subjected to supervised (adjusted to the origin of the primary site) average-linkage hierarchical clustering. Normal tissue mucosa and primary tumours grouped separately on a clustering dendrogram (Fig. 1A) as well as normal tissue mucosa and metastasis (data not shown). Comparison of primary tumours *vs*. metastasis showed only marginal differences, displaying a rather gradual transition (Fig. 2A). As expected, these results show that metastasis and primary tumour were closely related and could not be clearly distinguished from each other.

In order to pinpoint the most important upregulated genes for either PT*vs*.N or M*vs*.PT, we restricted the dataset to a p-value of lower than 0.0001 (corresponds to a FDR of 0.003 in PT*vs*.N and 0.004 in M*vs*.PT) and a log_2_ fold change (FC) higher than 1.5 (equates to a FC of 2.83). This data restriction resulted in a signature gene set of 38 upregulated genes for PT*vs*.N (Fig. 1B and Table S8), and 62 upregulated genes for M*vs*.PT respectively (Fig. 2B and Table S9).

Several proteins involved in the (re-)organization and assembly of the extracellular matrix were significantly overexpressed in all primary tumours compared to normal tissue mucosa (Table S8): Matrix Metallopeptidases, Collagens, Asporin, Fibronectin, Laminin, Lysyl Oxidases, Microfibrillar-Associated Protein, Periostin, Serpin Inhibitors, Osteonectin and Osteopontin. Higher expression of genes like *KIF23, UBE2C, MCM7, NUP107, RSRC1, MAD2, BTAK, RFC5, ECT2* and *CENPA* was also found in metastatic tumours reports from other studies^14^. The latter 5 genes were also present in the distant recurrence predictor of van’t Veer *et al*. for breast cancer^28^. Regarding the comparison of M*vs*.PT (Table S9), we identified upregulation of several genes related to the immune response, cellular metabolism, several membrane proteins, as well as genes interacting with or regulating GTPases.

### Ingenuity Analysis of significantly up-/downregulated genes

**Figure 3:**
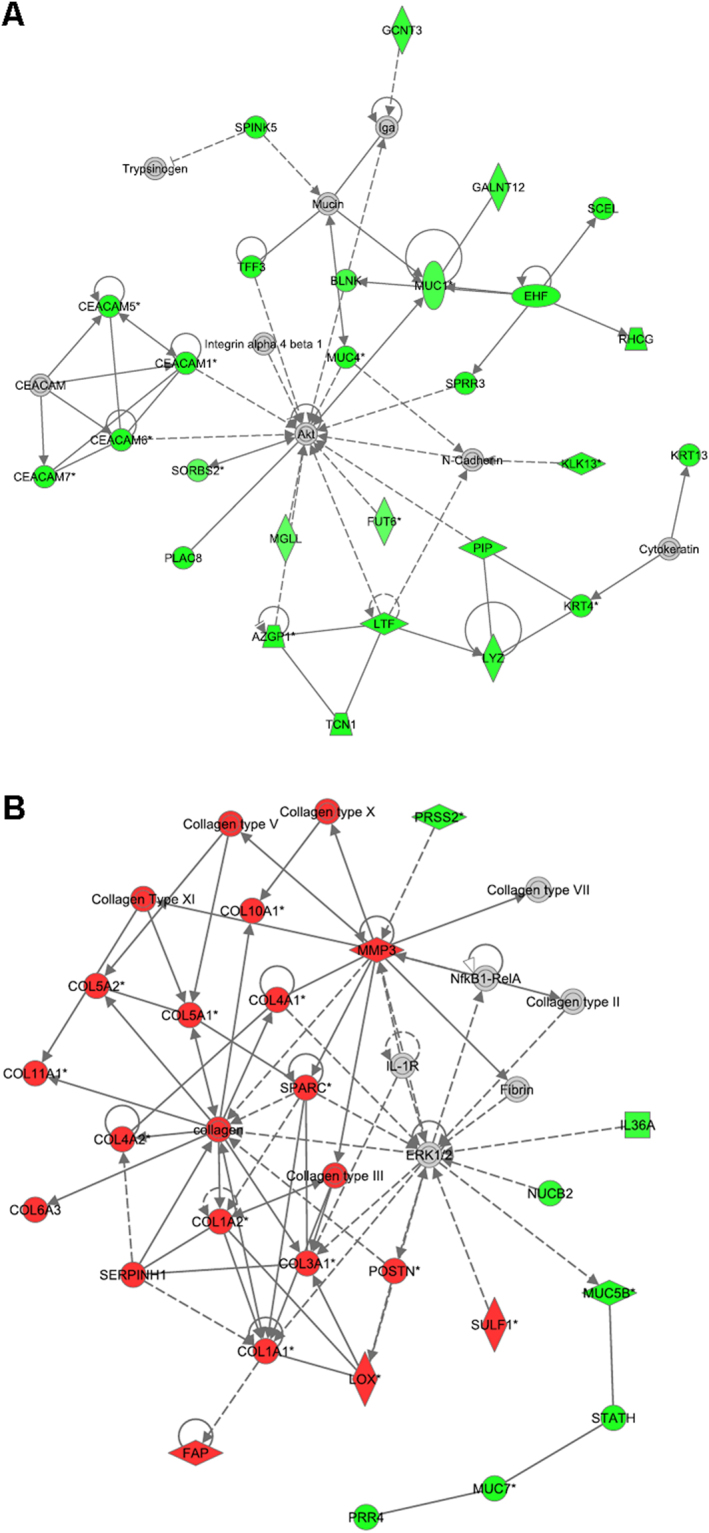
Top networks of differentially expressed genes in PT*vs*.N as identified by Ingenuity Pathway Analysis show strong association to cancer and connective tissue disorders. For network analyses a threshold of log_2_FC > |1.5| with a p-value < 0.0001 (corresponding to a FDR of 0.003) was used, giving an overview of involved genes within the networks and their differential expression. Node colour indicates up-regulated (red), down-regulated (green) or not significantly changed according to threshold (grey) genes. Lines and arrows between nodes represent direct (solid lines) and indirect (dashed lines) interactions between molecules. All edges are supported by at least one reference from the literature or from canonical information stored in the Ingenuity Knowledge Base. Node shapes represent functional classes of gene products: square → cytokine, triangle → kinase, rectangle → nuclear receptor, concentric circle → group or complex, vertical diamond → enzyme, horizontal diamond → peptidase, trapezium → transporter, vertical ellipse → transmembrane receptor horizontal ellipse → transcription regulator, circle → other. (**A**) Top 01 significant PT*vs*.N, network (Cellular Development, Infectious Disease, Cancer) contains 27 focus genes. (**B**) Top 02 significant PT*vs*.N network (Connective Tissue Disorders, Dermatological Diseases and Conditions, Cellular Assembly and Organization) contains 24 focus genes.

**Figure 4:**
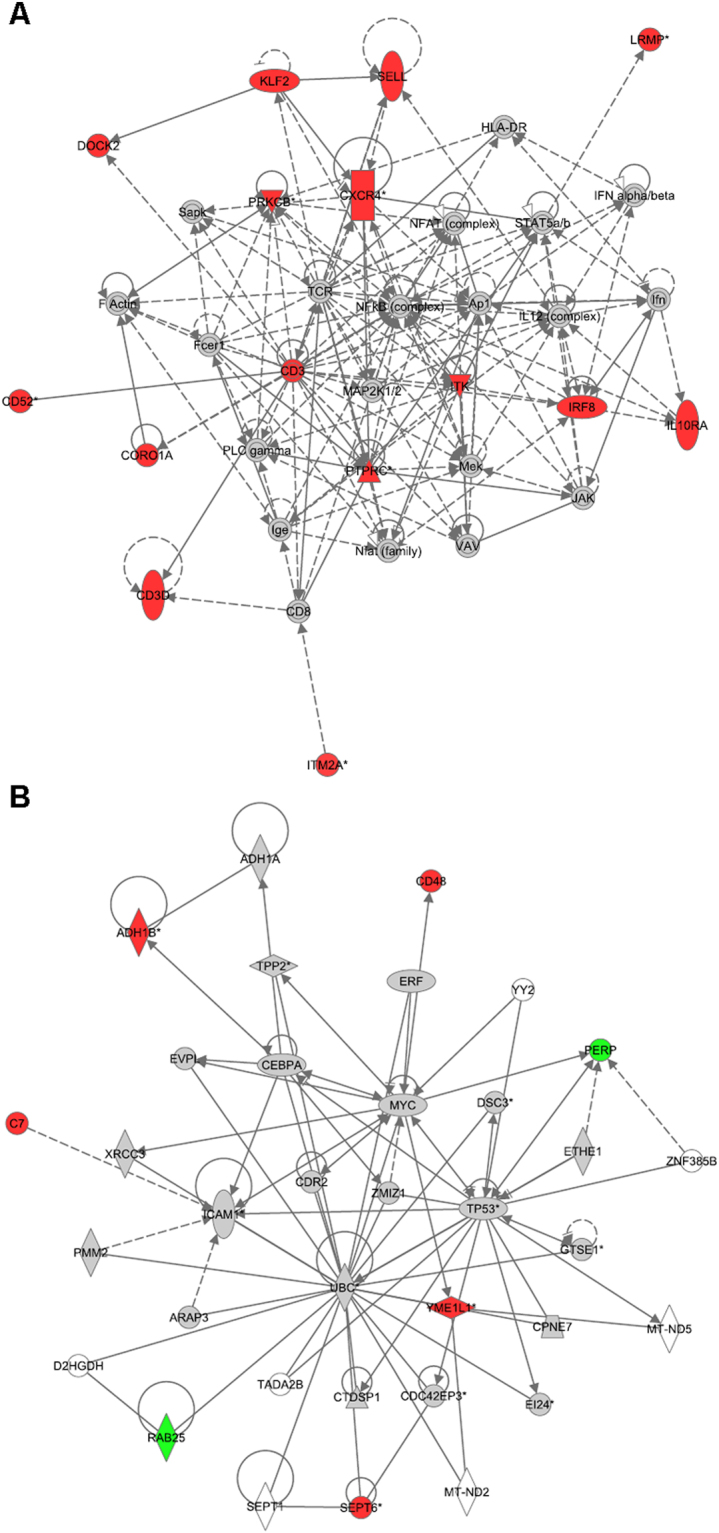
Top networks of differentially expressed genes in M*vs*.PT as identified by Ingenuity Pathway Analysis show strong association to cancer and connective tissue disorders. For network analyses a threshold of log_2_FC > |1.5| with a p-value < 0.0001 (corresponding to a FDR of 0.004) was used, giving an overview of involved genes within the networks and their differential expression. Node colour indicates up-regulated (red), down-regulated (green) or not significantly changed according to threshold (grey) genes. Lines and arrows between nodes represent direct (solid lines) and indirect (dashed lines) interactions between molecules. All edges are supported by at least one reference from the literature or from canonical information stored in the Ingenuity Knowledge Base. Node shapes represent functional classes of gene products: square → cytokine, triangle → kinase, rectangle → nuclear receptor, concentric circle → group or complex, vertical diamond → enzyme, horizontal diamond → peptidase, trapezium → transporter, vertical ellipse → transmembrane receptor horizontal ellipse → transcription regulator, circle → other. (**A**) Top 03 significant M*vs*.PT network (Cell-To-Cell Signalling and Interaction, Haematological System Development and Function, Tissue Morphology) contains 14 focus genes. (**B**) Top 07 significant M*vs*.PT network (Cancer, Endocrine System Disorders, Developmental Disorder) contains 7 focus genes.

Previously reported correlations were investigated thoroughly using the Ingenuity Pathway Analysis tool to reinforce the validity of our analysis. We applied the same restrictions as before, but included downregulated genes (p-value of lower than 0.0001 and higher than a log_2_FC of |1.5|) to our Ingenuity analysis. Comparing gene expression profiles of PT*vs*.N, we identified several disease/function networks for cancer and connective tissue disorders with a score higher than 20 (Fig. 3 and Table S10). For the dataset M*vs*.PT, top networks included one network for immune response, as well as several networks for cell movement, cell-to-cell signalling and interaction with a score higher than 20 (Fig. 4 and Table S11). All top networks are illustrated in detail in Supplementary Figures S12–S27.

### Ingenuity Analysis of upstream regulators

To further understand the context of activated networks, we used the upstream analysis tool of the Ingenuity software. Among the analysed activated upstream regulators for PT*vs*.N (z-score higher than 2) we found glycoprotein CD44, a stem cell marker for HNSCC^29^ and several growth factors like FGF2, TGFB1 and TGFB3 (Table 1A). Notably, there were only two significantly activated upstream regulators in Mvs.PT, namely the PI3K family which has been implicated with a more aggressive and invasive tumour type^8,30^ and the cytokine IFNγ which has previously been suggested as a salivary biomarker for early diagnosis of oral cancer^31^ (Table 2).

**Table 1:** Activated/Inhibited Upstream Regulators (Ingenuity) PT*vs*.N.

Upstream Regulator	Log Ratio	Molecule Type	Predicted Activation State	Activation z-score	p-value of overlap
CD44	0.382	enzyme	Activated	2.137	6.39E-09
FGF2	−0.133	growth factor	Activated	2.261	1.08E-10
IL1A	0.090	cytokine	Activated	2.298	1.51E-11
TGFB3	0.471	growth factor	Activated	2.416	3.72E-09
Ap1		complex	Activated	2.581	9.43E-08
CTGF	0.357	growth factor	Activated	2.930	2.80E-08
TGFB1	0.688	growth factor	Activated	3.316	2.75E-09
EHF	−2.432	transcription regulator	Inhibited	−2.333	6.02E-08
MYC	−0.071	transcription regulator	Inhibited	−2.433	4.50E-10
WISP2	−0.000	growth factor	Inhibited	−2.630	1.64E-07
ABCB4	−0.054	transporter	Inhibited	−2.631	1.16E-08
CR1L		other	Inhibited	−2.804	4.24E-10
FBN1	0.528	other	Inhibited	−2.804	5.11E-12
estrogen receptor		group	Inhibited	−2.937	2.28E-10
SPDEF	−0.558	transcription regulator	Inhibited	−3.000	3.99E-08
AHR	0.269	ligand-dependent nuclear receptor	Inhibited	−3.063	7.40E-10

**Table 2:** Activated/Inhibited Upstream Regulators (Ingenuity) M*vs*.PT.

Upstream Regulator	Log Ratio	Molecule Type	Predicted Activation State	Activation z-score	p-value of overlap
PI3K (family)		group	Activated	2.216	3.43E-05
IFNG	−0.038	cytokine	Activated	2.449	1.48E-05

Surprisingly, inhibited upstream regulators (z-score lower than -2) for PT*vs*.N included the oncogene *MYC* (Table 1). There were no significantly inhibited upstream regulators for M*vs*.PT (Table 2). The Ingenuity Comparison Analysis of selected canonical pathways showed a high relevance for cancer pathways and tissue morphology in both primary tumours as well as metastases, whereas cell movement and inflammatory response were predicted more prominent just in metastases (Figs S8 and S9).

Further downstream analysis revealed several interesting networks, one of them being activated by Fibronectin 1 (FN1) in PT*vs*.N (Fig. S5). Since FN1 is involved in several cellular processes, such as remodelling of the extracellular matrix, it is of general note to investigate the expression and activation status of its downstream targets. This will allow the identification of potential activation processes and prediction of corresponding cellular processes. Downstream targets of FN1 include VEGFA, the Jnk Family, the Tgf beta family, HRAS and the ERK1/2 family, which collectively activate diverse cellular functions via the transcription factor ETS1 (expression of cytokine and chemokine genes), beta-Catenin (key downstream component of Wnt signalling), the transcription factor Jun-B (regulation of gene activity following growth factor response), the corresponding nuclear phosphoprotein c-Fos and Ap-1, as well as the transcription factor SP1. All of these genes were activated and/or overexpressed in our dataset PT*vs*.N (Fig. S5).

The PI3K family has been shown to be essentially important for metastatic transformation^30^ and is the most significant activated upstream activator in M*vs*.PT (Table 2). The PI3K group activates NFkB via Akt and promotes the release of MMP9, which is the most important matrix metalloprotease in degrading the basement membrane, which is also predicted by Ingenuity (Fig. S6). The NFkB complex usually activates DNA damage response via RELA and RELB, blocking PPARG and the Jnk family but this could not be confirmed by our Ingenuity prediction for M*vs*.PT (Fig. S7). The nuclear receptor PPAR gamma is activated, which should block pro-inflammatory NFkB-mediated responses^32^. In contrast, tumour-promoting cytokines, such as TNF-α and IL10 are upregulated (Fig. S7) underlining the complexity and crosslinking of malignant signalling pathways as previously described^33^.

Deregulated canonical pathways are essential for tumour maintenance and important factors of malignancy. For both primary tumours (Fig. S8) and metastases (Fig. S9), the pathways of Nicotine Degradation and Ethanol Degradation were activated (Figs S10 and S11). Since all patients were smokers and alcohol consumers (Table S1), those findings are in accordance to our expectations.

### Comparison of amplification profiles of next generation genome sequencing data to our analysis

Since next generation sequencing (NGS) has had a major impact on cancer research, we compared recent NGS amplification^34^ and PARADIGM prediction data^34^ based on RNA sequencing data from the Broad Institute TCGA Genome database^35^ to our microarray analysis. Here, a log_2_FC higher than 0.263 was used as an overexpression tendency (corresponding to a 20% higher expression). For many genes, we found a concordant tendency of their regulation when we compared the genomic amplification and PARADIGM percentages (AMP) published by The Cancer Genome Atlas (TCGA) project^36^ and our expressional data (Table 3). Especially *FGFR1* (13% in PT*vs*.N and 7% in M*vs*.PT to 10% in AMP), *FGFR3* (7% in PT*vs*.N and M*vs*.PT to 2% in AMP), *DDR2* in lymph node metastasis (13% in M*vs*.PT to 3% in AMP) and *MET* (7% in PT*vs*.N and M*vs*.PT to 2% AMP, FC 2.4; Fig. S28) seem to be similarly overexpressed. Remarkably, we did not find a significant overexpression of *EGFR*, neither in the primary tumour, nor in their corresponding lymph node metastasis. In contrast, RNA-Seq-based amplification data suggested an AMP of 15% for *EGFR*. The receptor *Her-2 (ERBB2*), which was amplified in 4% of specimens in TCGA cohort, was mostly downregulated in our expression data as well as the RNA-Seq data (FC 0.479; Fig. S28). *MYC*, which was shown to be inactivated in our Ingenuity upstream analysis, was overexpressed in 7% of patients, compared to 14% amplification in TCGA samples. *E2F1* and *NFE2L2* overexpression values were both higher than expected by TCGA data, and even more elevated in metastasis than in primary tumours. We also found a higher expression for *EPHA2, DDR2* and *PIK3R1* compared to AMP published by TCGA. No significant differences between the fold changes of the PT*vs*.N to the M*vs*.N dataset could be detected for *FGFR1, IGF1R, FGFR2, MET, HRAS, PTEN* and *NF1. ERBB2, FGFR3* and *MYC* overexpression percentages were significantly higher in primary tumours than in metastases.

**Table 3:** Comparison of Microarray Expression Data with RNA-Seq Data.

		Upregulation of 20% or higher		TCGA RNA-Seq* AMP**	
Group	Gene	% in PT*vs*.N	% in M*vs*.N	HPV(−)	HPV(+)
(R)TK	EGFR	0	0	15↑	6↑
	ERBB2	0	0	4↑	3↑
	FGFR1	13	7	10↑	0
	FGFR3	7	7	2↑	11↑
	IGF1R	0	0	4↑	0
	EPHA2	0	0	4 m	3 m
	DDR2	0	13	3↑	6↑
	MET	7	7	2↑	0
Oncogenes	CCND1	7	0	31↑	3↑
	MYC	7	7	14↑	3↑
	HRAS	13	13	5 m	0
PI(3)K	PIK3CA	7	0	34↑/m	56↑/m
Survival	BIRC2	7	0	7↑	3↑
	FADD	33	13	32↑	6↑
	E2F1	0	7	2↑	19↑
Other	TP63	13	0	19↑	28↑
	NFE2L2	0	0	14↑	0
	NF1	0	0	3↑/m	0

^*^TCGA RNA-Seq: The RNA sequencing data as published by The Cancer Genome Atlas Network^36^; the Broad Institute TCGA Genome Data Analysis Center^35^ and the tumorportal.org^91^.

^**^AMP: genomic amplification percentage (AMP) as published by The Cancer Genome Atlas Network^36^. ↑ predicted as amplified; ↑/m predicted as amplified or mutated.

### Comparison of individual Receptor Tyrosine Kinase (RTK) expression patterns

**Figure 5:**
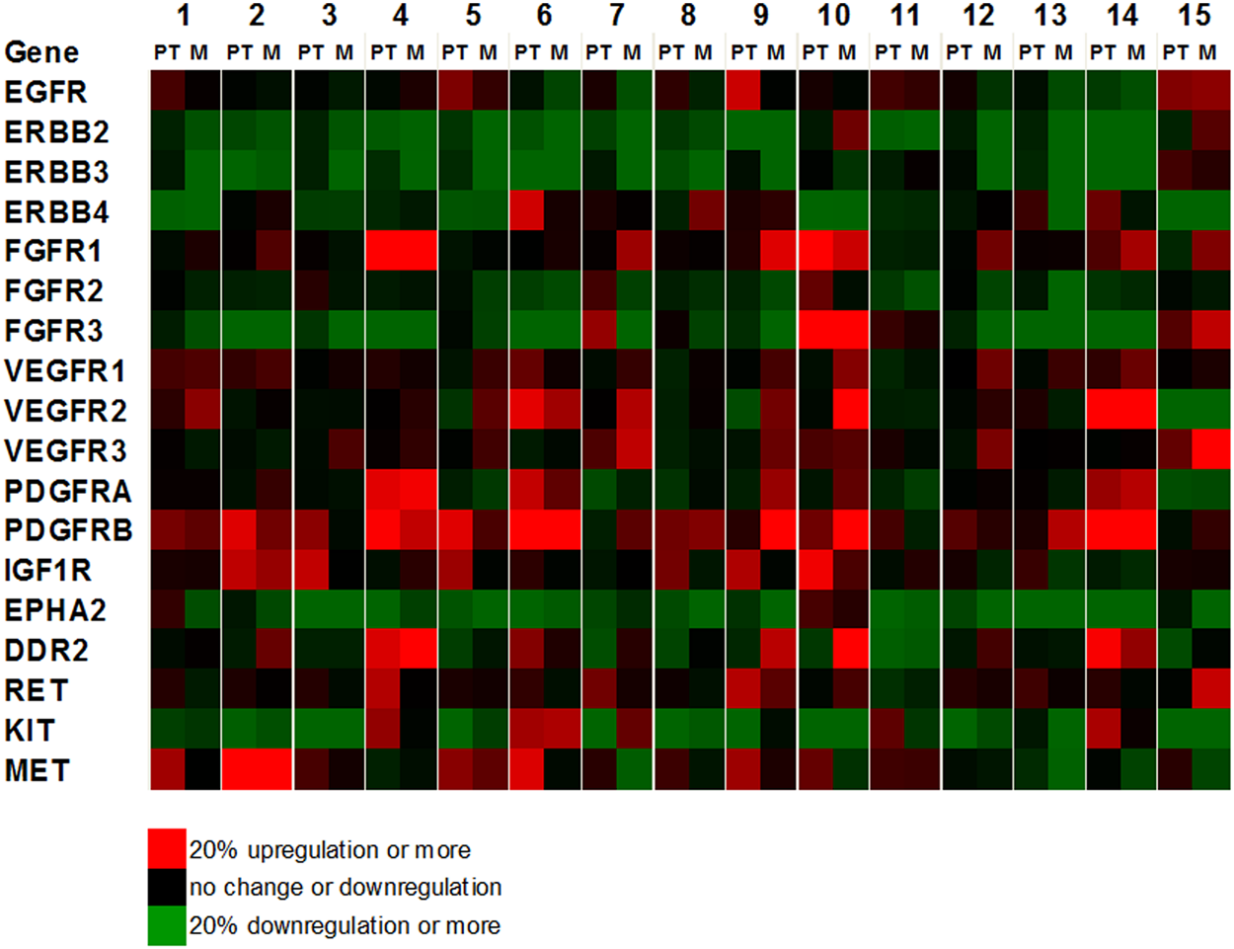
Comparison of Receptor Tyrosine Kinase (RTK) Expression of all patients. Differential expression values for selected genes were colour coded (bright red for an overexpression of ≥20%, bright green for a downregulation of ≥20%). In a ratio to their corresponding normal tissue mucosa, primary tumour values (PT) and metastasis values (M) are listed for each patient 1–15.

Due to their crucial role in HNSCC as upstream regulators of e.g. the phosphoinositol-3-kinase (PI3K) pathway mediating invasiveness and malignancy^30^, we concentrated on further exploring the expression of growth factor receptors (receptor tyrosine kinases, RTKs). Remarkably, in our study PI3K was one of only two significantly activated upstream regulators in M*vs*.PT (Table 2). Based on the list of amplified genes from the HNSCC TCGA project, we created an overview of relative RTK expression for PT*vs*.N and M*vs*.N (Fig. 5). With the exception of two patients, all patients showed overexpression tendency of at least one RTK indicated by an upregulation of at least 20%. Most patients showed an upregulation of random combinations of at least two RTKs. For example, patient 6 showed an upregulation combination of *ERBB4, VEGFR2* and *PDGFRB*, and a slight upregulation of *MET* in PT*vs*.N. In patient 14, *VEGFR2, PDGFRB* and *DDR2* were higher expressed in comparison to normal mucosa. An expanded overview of RTK and oncogene expression can be found in the Supplementary Material (Fig. S29).

## Discussion

Precision medicine requires individualized therapeutic approaches that fulfill the needs of each patient. Therefore, both prognostic as well as predictive markers are a crucial prerequisite. The thorough understanding of underlying mechanisms of tumorigenicity is not only the basis for the development of new drugs, but also for biomarker development.

In this study we analysed and compared the expression profiles of 15 primary tumours to their corresponding normal tissue mucosa and paired lymph node metastasis. In contrast, previous microarray-based studies of HNSCC have focused only on primary tumours versus normal mucosa or primary tumour patterns of expression^16–21,37^. Since the number of patients is relatively low, p-values have to be interpreted with caution^38^. We are aware of the limitations of this study and recommend replicate tests for their verification.

**Figure 6:**
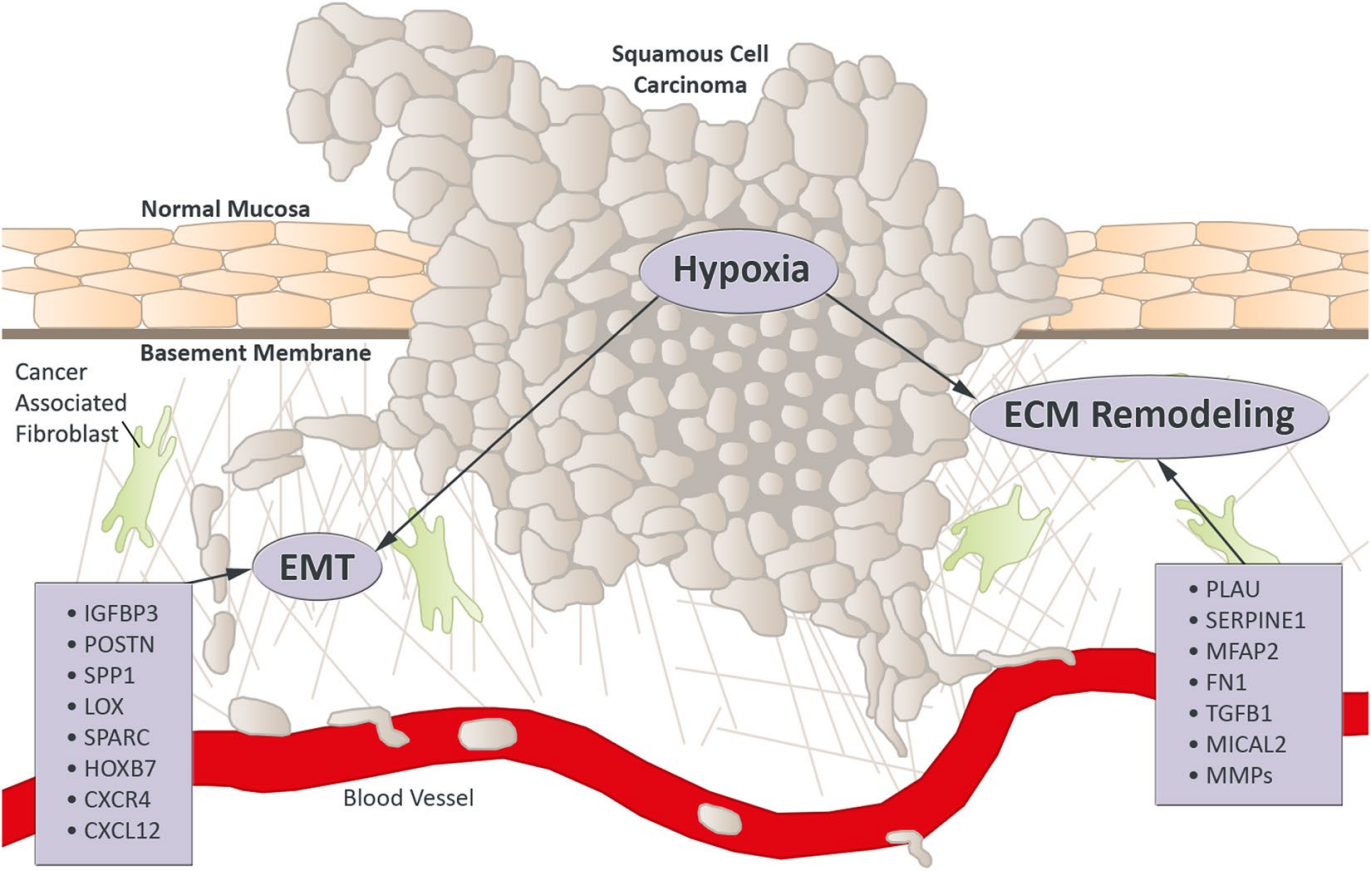
Potential prognostic biomarkers for HNSCC linked to hypoxia, EMT and ECM remodelling are directly connected. Hypoxic environments induce genes promoting an aggressive, invasive phenotype, mediated by epithelial to mesenchymal transition (EMT) and remodelling of the extracellular matrix (ECM). Matrix metalloproteinases (MMPs) and other proteases degrade the basement membrane and new collagen fibres are aligned by modifying enzymes to facilitate local invasion and recruitment of cancer associated fibroblasts.

We have identified and verified several genes and pathways that are significantly overexpressed in HNSCC tumours with lymph node metastases. Based on this study, we here provide a model how genes involved in tumorigenesis and metastatic progression are differentially expressed in HNSCC, involving changes in the extracellular matrix, hypoxia and EMT (overview in Fig. 6). Our finding of highly upregulated matrix metalloproteases, integrins, collagens, fibronectin, laminin and other ECM proteins supports previous studies showing that degradation of the basement membrane and remodelling of the extracellular matrix are essential during the invasion process of a tumour^39–41^.

Molecules Interacting with CasL-2 Proteins (MICALs) are monooxygenases that cause actin to depolymerize and are implicated in the regulation of cytoskeletal dynamics, intracellular trafficking, the docking and fusion of exocytotic vesicles and also have anti-apoptotic properties^42^. MICAL2, which was significantly upregulated in our PT*vs*.N dataset, has been identified as one of the most significantly upregulated genes in smoking-induced lung cancer^43^ and has been associated with prostate cancer progression^44^ as well as EMT, cancer growth and invasion^45^, but has not been implicated with head and neck cancer, yet.

The destruction and remodelling of the extracellular matrix not only drives the mechanical and proteolytic invasion of tumour cells, but also favours metastatic cell reprogramming and behaviour^46–48^. Specialized Cancer Associated Fibroblasts (CAFs) can degrade various ECM proteins like collagens, basement membrane laminin and fibronectin^46^, leading to a more aggressive behaviour of the cancer, lymph node metastasis and poor prognosis, as shown in oral cancer^49,50^. When the Chemokine C-X-C Motif Receptor Type 4 (CXCR4), whose expression can be enhanced by hypoxia, binds Stromal Cell-derived Factor 1 (CXCL12), secreted by CAFs, release of MMP-9 to the tumour microenvironment is stimulated^51,52^. In our data, we identified a switch from MMP1, 3 and 13 in primary tumours to MMP1, 9 and 12 in metastases, as well as a significant upregulation of CXCL12/CXCR4. Resulting MMP levels and the ratio of activated MMPs to total MMP concentration compared to adjacent normal tissue is positively correlated with lymph node involvement^51,53^. MMP9 is the most versatile of the MMPs and can degrade various elements of the tumour microenvironment, like elastin, fibrillin, laminin, gelatin and collagens of type IV, V, XI and XVI^52,54^.

Hypoxia is very common in solid tumours, especially in HNSCC^55^, which has repeatedly been associated with poor prognosis^56^. Additionally, hypoxic environments induce genes supporting stem cell maintenance, angiogenesis and epithelial to mesenchymal transition (EMT), leading to an aggressive, invasive phenotype^57–59^. CXCR4 inhibitors have most recently been suggested as treatment for advanced and metastasizing HNSCC^60^, and have previously been reported to have synergistic effects with anti-PDL1 therapies^61^.

Homeobox B7 (HOXB7) is a developmental gene we found highly upregulated in our PT*vs*.N dataset. HOXB7 confers epithelial-mesenchymal transition in breast cancer^62^, promotes malignant progression by activation of TGF signalling^63^ and lung metastasis^64^ and renders breast cancer cells resistant to tamoxifen through activation of the EGFR pathway^65^. HOXB7 overexpression is associated with poor prognosis^66^ and has been suggested as a prognostic factor for oral squamous cell carcinoma^67^. Migration of endothelial progenitor cells is mediated by CXCL12/CXCR4 via PI3K/Akt/eNOS signalling^68^. The CXCL12/CXCR4 system also facilitates lymph node metastatic potential in oral squamous cell carcinoma by enabling EMT^69^.

The phosphoinositol-3-kinase (PI3K) pathway is the most frequently altered oncogenic driver in head and neck cancer and its upstream regulators and downstream targets might be the most interesting targets for predictive biomarkers^30,70^. While we could not show a single significantly upregulated growth factor receptor for all patients, we could confirm the global upregulation of the pathway in our Ingenuity Analysis. Interestingly, most patients showed a tendency for upregulation of at least two RTKs in varying combinations (Fig. 5). Several correlations involving sets of RTKs have previously been shown in different tumour entities. A meta-analysis for breast cancer has indicated the association of an overexpression of various RTKs with poor outcomes and suggested a clinical evaluation of combination agents against RTKs or relevant oncogenic nodes^71^. In breast cancer, MEK inhibition resulted in acute ERK activity loss and subsequent c-MYC degradation that induced expression and activation of PDGFRB, DDR2 and VEGFR2^72^, an RTK combination found in patients 6 (PT) and 10 (M) of our dataset (Fig. 5). It has been proposed that upregulation of FGFRs (as seen in patients 4, 9 and 10, as well as to a minor degree in patients 7, 12, 14 and 15, Fig. 5) could be involved in the development of resistance to anti-VEGFR therapies, since both pathways are regulating tumour neoangiogenesis (as reviewed in^73^). In breast cancer patients with *FGFR1* or *FGF3* amplification, multikinase FGFR/VEGFR inhibitors have shown promising activity^74^. It has been suggested to treat RTK-driven tumours by hitting signalling nodes interconnecting core pathways, targeting c-Abl as a signalling node connecting MET and TP53^75,76^ (cp. patients 2, 5, 6 and 9, Figs 5 and S29).

In comparison with recent high throughput sequencing data, we showed a comprehensive list of upregulated genes for each patient. Regarding numerous targeted therapies for HNSCC currently in clinical studies^26^, it will be essential to develop predictive biomarkers allowing the selection of an optimal therapy for each patient. Currently, molecular companion diagnostics are still lacking for all therapeutic approaches in HNSCC. For Cetuximab, the only targeted therapy currently in clinical use for HNSCC, the development of acneiform rash is still the best indicator for therapy response^77^. *EGFR*-expression status, determined by immunohistochemistry, could not be verified as a predictive marker for EGFR-targeted therapy response^78,79^, even though mechanisms of action and resistance mechanisms have been thoroughly investigated (as reviewed in^80,81^). *EGFR* is amplified in normal mucosa of tobacco using patients, and might thereby be not the oncogenic driver, but rather a response to carcinogenic agents^81^. This might explain our results for the comparison of *EGFR* expression in primary tumour vs. normal mucosa (Table 3 and Fig. 5).

The combination of NGS with other approaches is most promising for the development of clinically relevant, molecular subgroups that will guide treatment (as reviewed in^82^). Whole genome expression profiling, including mutational analysis by RNA-Seq might surpass the limitations of microarray technology, but currently is still too expensive to be included in large multi-centered clinical studies. This experimental approach might also lack reproducibility due to different platforms and handling. Additionally, the large amount of data produced by high throughput sequencing techniques has to be processed and interpreted carefully. Microarrays may no longer be the standard for basic scientific research, but are currently miniaturized and automated as a cost-effective on-chip nanotechnology for clinical diagnostic approaches^83,84^. We suggest analysing a selection of biomarker candidates by customized automated microarrays, which should be narrowed down by previous next generation sequencing approaches. These analyses should be performed parallel to clinical trials for HNSCC and other types of cancer. Correlating a small list of chosen potential biomarkers to clinical data of outcomes and treatment efficiency might facilitate following health technology assessment for drug approval as well as biomarker development. Such approaches should also be employed for investigating the pathobiological relevance of so called ‘e-liquids’, currently widely considered as a more ‘healthy’ way of smoking by the population.

In conclusion, we here shed new light on the expression differences in lymph node metastasis compared to primary HNSCC tumours, and provide the data for further analyses.

## Material and Methods

### Reagents

All reagents were purchased from Sigma unless stated otherwise.

### Patient characteristics and material

Tissue samples were obtained from 15 patients undergoing surgical resection at the departments of otolaryngology of the Universities of Frankfurt and Mainz. Investigation has been conducted in accordance with the ethical standards according to the Declaration of Helsinki and according to national and international guidelines. The study protocol has been approved by the ethics committees “Ethik-Kommission des Fachbereichs Medizin, Universitätsklinikum der Goethe-Universität” (#83756604) and “Ethik-Kommission der Landesärztekammer Rheinland-Pfalz” (#83748515) after obtaining the patients’ informed consent to participate in the study and was processed anonymously. All cases were diagnosed histopathologically as HNSCC and staged according to the TNM classification of malignant tumours recommended by the ‘Union International contre le Cancer’ UICC. In this study, tumour specimens, corresponding non-malignant tissue from the oral cavity (buccal mucosa), and lymph node metastasis from each patient were analysed. The median age of patients was 56 years and 86% were males. Six of the resected tumours were classified as grade 2 (40%), nine as grade 3 (60%). Twelve patients had a lymph node status of N1/2 (80%) and three of N3 (20%). The majority of cancers were located in the hypopharynx (60%), 13.3% in the larynx and 26.6% in the oropharynx (Table S1). Specimens included oropharyngeal and laryngopharyngeal carcinoma of different tumour size (T1–T4) and lymph node status (N1-3), without distant metastasis (M0). Additional inclusion criteria were positive alcohol and tobacco use, HPV-negative status of the primary tumour, a histopathological grading of 2–3 and the patients did not have previous cancer treatment before surgery. Upon resection, samples were immediately placed on ice and snap-frozen in liquid nitrogen within 30 min. Histological analyses were performed to ensure that each specimen of primary tumours and metastasis contained >70% tumour cells and <10% necrotic debris. Normal mucosa was histopathologically checked for non-malignancy. All criteria were cross-checked and approved by the independent analyses of at least two qualified head and neck pathologists. Samples not meeting these criteria were rejected.

### RNA extraction

Frozen tissue samples (30–50 mg) were collected in 1 ml Trizol (Invitrogen, Karlsruhe, Germany) and dispersed using an Ultra-Turrax T25 tissue homogenizer (IKA Werke, Staufen, Germany). Total RNA was extracted according to the recommendations given by the manufacturer’s Trizol protocol and further purified on RNeasy Mini spin columns (Qiagen, Hilden, Germany). Integrity and purity of total RNA were assessed on a Bioanalyzer 2100 (Agilent Technologies, Boeblingen, Germany) using RNA 6000 Nano LabChip Kit (Agilent) according to the manufacturer’s instructions as previously described^85^, accepting absorbance ratios of 1.8–2.2 for A_260_/A_280_ and a A_260_/A_230_ ratio of lower than 1.7. Samples not meeting these criteria were rejected.

### Reverse transcription (RT)-PCR

RT-PCR was performed using specific primer sets for OSF2, FN1, KRT24, CLCA4, PRR4, SCCA2b, TP73L, LYPD3, FCMR, ARHGAP25 and RASGRP2 genes (Table S9). GAPDH was used to control the amounts of cDNA generated from each sample as described^85–87^. First-strand cDNA synthesis was carried out using a cDNA synthesis kit for RT-PCR (Superscript II, Invitrogen life technologies). 1 µg of total RNA was converted to cDNA and 1 µl of the 20 µl RT product was amplified for 30 cycles (initial denaturation at 95 °C for 3 min, 30 sec at 95 °C, 30 sec at a variable temperature for annealing and 1 min at 72 °C) followed by an extension of 5 min at 72 °C. RT-PCR amplification products were analysed on 2% agarose gel stained with ethidium bromide.

### Quantitative Real-Time PCR analysis

For ARHGAP25, SERPINB4 and PRR4, changes in mRNA levels detected in microarray experiments were evaluated by quantitative real-time PCR analysis, using the iCycler (BioRad, Munich, Germany). One µg of total RNA was converted to cDNA using Superscript II (Invitrogen Corporation) and oligo(dT) primer according to manufacturer’s specifications. PCR reaction mixtures consisted of 12.5 µl of 2× iQ SYBER Green Supermix (Abgene, UK), 0.5 µl of each 10 µM target primer, 1 µl (1:10) cDNA template in a final scaled down reaction volume of 25 µl. Thermal cycling conditions comprised an initial denaturation step at 95 °C for 15 min, 40 cycles at 95 °C for 30 sec and variable annealing temperatures for 30 sec depending on the respective set of target primers. Cumulative fluorescence was measured at the end of the extension phase of each cycle. Specific amplicon formation with each primer pair was confirmed by dissociation curve analysis and by visualization of a single band on a 2% agarose gel. To define the relative expression of the genes, the PCR product results from each tumour sample were compared with the results from normal tissue or lymph node metastasis from the same patient. The relative expression ratio (R) of target gene is calculated using the equation: Ratio = (E_target_)^ΔCp target(control-sample)^/(E_ref_)^ΔCpref(control-sample)^ based on its real-time PCR efficiencies (E) and the crossing point (CP) difference of an unknown sample versus a control and expressed relative to the non-regulated housekeeping gene glyceraldehyde-3-phosphate dehydrogenase gene (GAPDH), as described^86,88^.

### Target preparation and hybridization for Affymetrix GeneChip Arrays

Total RNA (5 µg) was used to prepare biotinylated cRNAs for hybridization, following the guidelines given in the Affymetrix GeneChip Expression Analysis Technical Manual. cRNA clean-up was performed on RNeasy Mini filters (Qiagen). In all, 10 µg of fragmented, labelled cRNA were hybridized to Affymetrix HG-U133A arrays (Affymetrix, Santa Clara, CA, USA) using standard conditions (16 h, 45 °C). Arrays were washed and stained in a Fluidics Station 400 (Affymetrix) and scanned on a Gene Array Scanner 2500 (Agilent), as recommended by Affymetrix.

### Statistical Analysis

Analysis of the data was performed using R 2.15.2 with the *limma* package 3.14.4. Raw fluorescence intensities from all hybridizations were normalized, applying robust multichip average (RMA) normalization for the. CEL data, followed by a quantile normalization to compare expression results across specimens^89,90^. For the comparison of primary tumours to normal mucosa (PT*vs*.N), metastasis to normal mucosa (M*vs*.N) and metastasis to primary tumours (M*vs*.PT) of differentially expressed genes, we also utilized the *limma* package and adjusted our model for each tissue type (supervised clustering). For multiple testing a Bonferroni correction was performed. Due to the large number of tests applied in this study, p-values have to be interpreted with caution.

For comparative analysis of our microarray analysis to NGS amplification data^34^ and PARADIGM prediction data (including RNA-Seq)^34^, we used genomic amplification percentages (AMP). AMP specifies the percentage of samples for which amplification of a respective gene was published by The Cancer Genome Atlas (TCGA) project^36^. It should be noted that in our study we just compared data of HPV-negative tumours.

### Ingenuity analysis

The Ingenuity Pathway Analysis (IPA) tool (Ingenuity Systems) was used to identify pathways related to enriched genes in primary tumours (PT*vs*.N) or metastasis (M*vs*.N), as well as primary tumours compared to metastasis (M*vs*.PT). Data was filtered to meet the criteria p < 0,0001 and a log_2_FC > |1,5| threshold in our comparison analysis. For each probeset, its Affymetrix probeset ID and its p-value from the comparison analysis were loaded into the analysis tool and mapped to its corresponding gene object in the Ingenuity Pathways Knowledge Base.

### Data availability statement

The datasets generated during and/or analysed during the current study are available from the corresponding author on reasonable request.

## Electronic supplementary material

Supplementary Information
