# Supplementary material for: Expressional analysis of disease-relevant signalling-pathways in primary tumours and metastasis of head and neck cancers
Source: Sci Rep. 2018 May 9;8:7326. doi: 10.1038/s41598-018-25512-7 (PMC5943339; doi:10.1038/s41598-018-25512-7)
Supplement: Supplementary file 1 — Supplementary Information [file 41598_2018_25512_MOESM1_ESM.pdf]

Supplementary Information

Expressional analysis of disease-relevant signalling-pathways in primary tumours and metastasis of head and neck cancers

Short title: HNSCC expression profiling

Dorothee Goesswein [1*] (MSc), Negusse Habtemichael [1] (PhD), Aslihan Gerhold-Ay [2] (PhD), Johanna Mazur [2] (PhD), Désirée Wünsch [1] (PhD), Shirley K. Knauer [3] (PhD), Julian Künzel [1] (MD), Christoph Matthias [1] (MD), Sebastian Strieth [1] (MD), Roland H. Stauber [1*] (PhD, MD)

[1] Molecular and Cellular Oncology, ENT/University Hospital of Mainz, Mainz, 55131, Germany

[2] Institute for Medical Biostatistics, Epidemiology and Informatics (IMBEI), University of Mainz Medical Center, Mainz, 55101, Germany

[3] Institute for Molecular Biology, Centre for Medical Biotechnology, University of Duisburg-Essen, Essen, 45117, Germany

***Corresponding authors:** Dorothee Goesswein and Roland Stauber, Molecular and Cellular Oncology, ENT/University Hospital of Mainz, Langenbeckstr. 1, 55131 Mainz, E-Mail: goesswein@uni-mainz.de; Roland.Stauber@unimedizin-mainz.de; Phone: +49 (0)6131 / 17-7002, Fax: +49 (0)6131 / 17-7661

Supplementary Information

1. Supplementary Tables
2. Supplementary Figures

1. Supplementary Tables

Tab. 1 – Clinical and histopathological features

Patient	Primary site	Age	Sex	TNM	Grade	HPV	Tobacco/ Alcohol
**1**	hypopharynx	47	f	T2 N2 M0	2	-	+/+
**2**	hypopharynx	46	m	T4 N1 M0	2	-	+/+
**3**	hypopharynx	48	m	T3 N1 M0	2	-	+/+
**4**	hypopharynx	58	m	T3 N2 M0	2	-	+/+
**5**	hypopharynx	72	m	T3 N2 M0	2	-	+/+
**6**	hypopharynx	57	m	T1 N2 M0	3	-	+/+
**7**	hypopharynx	57	m	T2 N3 M0	2	-	+/+
**8**	hypopharynx	47	m	T2 N2 M0	2	-	+/+
**9**	hypopharynx	56	m	T2 N3 M0	2	-	+/+
**10**	larynx	68	m	T4 N2 M0	2	-	+/+
**11**	larynx	56	m	T4 N3 M0	3	-	+/+
**12**	oropharynx	58	f	T3 N2 M0	2	-	+/+
**13**	oropharynx	56	m	T3 N2 M0	2	-	+/+
**14**	oropharynx	49	m	T3 N1 M0	2	-	+/+
**15**	oropharynx	53	m	T2 N2 M0	2	-	+/+

Tab. S2 – Microarray Data of RT-PCR verified genes (PT*vs.*N)

Affy ID	Gene	logFC	AveExpr	t	P.Value	adj.P.Val	B
**220026_at**	CLCA4	-4.05	7.26	-7.13	1.39E-08	4.85E-06	9.64
**210495_x_at**	FN1	1.78	11.20	5.25	5.65E-06	3.71E-04	3.89
**211719_x_at**	FN1	1.83	11.02	5.21	6.36E-06	4.01E-04	3.78
**216442_x_at**	FN1	1.83	11.12	5.20	6.67E-06	4.13E-04	3.73
**212464_s_at**	FN1	1.87	10.53	5.16	7.56E-06	4.49E-04	3.61
**214701_s_at**	FN1	1.00	6.33	4.04	2.41E-04	5.46E-03	0.32
**214702_at**	FN1	0.37	4.19	1.72	9.40E-02	3.14E-01	-5.06
**210809_s_at**	POSTN/OSF2	3.06	9.31	7.26	9.40E-09	3.43E-06	10.01
**220267_at**	KRT24	-2.97	6.39	-6.14	3.26E-07	4.40E-05	6.62
**204919_at**	PRR4	-5.24	7.55	-6.62	7.11E-08	1.54E-05	8.08

Tab. S3 – Microarray Data of RT-PCR verified genes (M*vs.*PT)

Affy ID	Gene	logFC	AveExpr	t	P.Value	adj.P.Val	B
**211906_s_at**	SERPINB4/SCCA2	-2.14	6.19	-4.41	7.96E-05	3.50E-03	1.47
**220804_s_at**	TP73	0.20	4.57	1.27	2.12E-01	4.14E-01	-5.47
**204952_at**	LYPD3/C4.4A	-1.32	8.73	-3.17	2.92E-03	3.07E-02	-1.84
**221601_s_at**	FAIM3	2.23	6.69	5.08	9.52E-06	1.18E-03	3.45
**221602_s_at**	FAIM3	1.50	6.75	4.84	2.03E-05	1.74E-03	2.74
**38149_at**	ARHGAP25	1.23	6.80	4.67	3.54E-05	2.31E-03	2.23
**204882_at**	ARHGAP25	1.15	6.88	3.72	6.16E-04	1.19E-02	-0.42
**214369_s_at**	RASGRP2	1.10	5.04	5.07	9.98E-06	1.21E-03	3.40
**208206_s_at**	RASGRP2	1.42	6.16	4.92	1.61E-05	1.54E-03	2.96
**214368_at**	RASGRP2	0.43	5.61	4.41	7.89E-05	3.49E-03	1.48
**214367_at**	RASGRP2	0.12	5.90	1.26	2.15E-01	4.17E-01	-5.48

Tab. S4 – Top 30 list of significantly up-regulated genes in PT*vs.*N ($log_2FC > 1.5$, p-value < 0.0001)

No.	Gene	Protein	Localization	Molecular function	Biological process
1	**MMP1**	Matrix Metallopeptidase1	Secreted Extracellular space Extracellular matrix	Hydrolase, Metalloprotease	Collagen degradation Host-virus interaction
2	**SPP1**	Osteopontin	Secreted	Cytokine	Biomineralization Cell adhesion
3	**POSTN**	Periostin, Osteoblast Specific Factor	Secreted Extracellular space Extracellular matrix	Cell adhesion	Heparin-binding
4	**COL1A1**	Collagen, Type I, Alpha 1	Secreted Extracellular space Extracellular matrix	Metal Ion Binding	-
5	**COL11A1**	Collagen, Type XI, Alpha 1	Secreted Extracellular space Extracellular matrix	Metal Ion Binding	-
6	**ASPN**	Asporin	Secreted Extracellular space Extracellular matrix	Calcium Ion Binding	Mineralization
7	**INHBA**	Inhibin, Beta A	Secreted	Growth Factor Hormone	-
8	**MMP3**	Matrix Metallopeptidase 3	Secreted Extracellular space Extracellular matrix	Hydrolase, Metalloprotease	Collagen degradation
9	**COL5A2**	Collagen, Type V, Alpha 2	Secreted Extracellular space Extracellular matrix	Metal Ion Binding	-
10	**COL1A2**	Collagen, Type I, Alpha 2	Secreted Extracellular space Extracellular matrix	Metal Ion Binding	-
11	**MMP13**	Matrix Metallopeptidase 13	Secreted Extracellular space Extracellular matrix	Hydrolase, Metalloprotease	Collagen degradation
12	**COL5A1**	Collagen, Type V, Alpha 1	Secreted Extracellular space Extracellular matrix	Metal Ion Binding	-

13	**CDH11**	Cadherin 11,Type 2 (OSF-4)	Cell membrane	Calcium Ion Binding	Cell Adhesion
14	**COL3A1**	Collagen, Type III, Alpha 1	Secreted Extracellular space Extracellular matrix	Metal Ion Binding	-
15	**LOX**	Lysyl Oxidase	Secreted Extracellular space	Oxidoreductase	
16	**LAMC2**	Laminin, Gamma 2	Secreted Extracellular space Extracellular matrix Basement membrane	Heparin-Binding	Cell Adhesion
17	**COL4A2**	Collagen, Type IV, Alpha 2	Secreted Extracellular space Extracellular matrix Basement membrane	Metal Ion Binding	Angiogenesis
18	**COL4A1**	Collagen, Type IV, Alpha 1	Secreted Extracellular space Extracellular matrix Basement membrane	Metal Ion Binding	Angiogenesis
19	**FN1**	Fibronectin 1	Secreted Extracellular space Extracellular matrix	Acute Phase Angiogenesis Cell Adhesion Cell Shape	-
20	**LOXL2**	Lysyl Oxidase-like 2	Secreted Extracellular space Extracellular matrix Basement membrane Nucleus	Chromatin Regulator Oxidoreductase Repressor	Transcription Regulation
21	**COL6A3**	Collagen, Type VI, Alpha 3	Secreted Extracellular space Extracellular matrix	Serine Protease Inhibitor	-
22	**SULF1**	Sulfatase1	Cell Surface	Hydrolase	Hydrolase
23	**MAGEA4**	Melanoma Antigen Family A, 4	-	-	-
24	**COL10A1**	Collagen, Type X, Alpha 1	Secreted Extracellular space Extracellular matrix	Metal Ion Binding	-
25	**SERPINE1**	Serpin Peptidase Inhibitor	Secreted	Protease Inhibitor Serine Protease Inhibitor	-

**26**	**FAP**	Fibroblast Activation Protein Alpha	Cell membrane	Hydrolase<br>Serine Protease	Angiogenesis<br>Apoptosis<br>Cell Adhesion
**27**	**MFAP2**	Microfibrillar-Associated Protein 2	Secreted<br>Extracellular space<br>Extracellular matrix	-	ECM organization
**28**	**SPARC**	Osteonectin	Secreted<br>Extracellular space<br>Extracellular matrix	-	Calcium<br>Copper<br>Metal-binding
**29**	**IGFBP3**	Insulin-Like Growth Factor Binding Protein 3	Secreted<br>Nucleus	Apoptosis	Growth factor Binding
**30**	**PXDN**	Peroxidasin Homolog (Drosophila)	Secreted<br>Extracellular space<br>Extracellular matrix	Oxidoreductase<br>Peroxidase	Hydrogen peroxide

Tab. S5 – Top 30 list of significantly down-regulated genes in PT*vs.*N (log_2FC < -1.5, p-value < 0.0001)

No.	Gene	Protein	Localization	Molecular function	Biological process
**1**	**STATH**	Statherin	Secreted	-	Biomineralization
**2**	**PRR4**	Proline-rich protein 4	Secreted	-	Retina Homeostasis Visual Perception
**3**	**CRNN**	Cornulin	Cytoplasm	Calcium Ion Binding	-
**4**	**CRISP3**	Cystein-rich secretory protein 3	Secreted	-	-
**5**	**KRT4**	Keratin, type II cytoskeletal 4	Cytoskeleton Nucleus	Structure Molecule	Intermediate Filament
**6**	**PIP**	Prolactin-inducible protein	Secreted	Actin Binding Glycoprotein Binding	Transmembrane Transport Bitter Taste Perception
**7**	**MAL**	Myelin and lymphocyte protein	Membrane	Lipid Binding Peptidase Activator Channel Activity	Apoptosis Myelination Cell Differentiation
**8**	**KRT13**	Keratin, type I cytoskeletal 13	Cytoskeleton Nucleus	Structure Molecule	Intermediate Filament
**9**	**SCEL**	Sciellin	Cytoplasm Membrane	Zinc Ion Binding	Epidermis Development Keratinocyte Differentiation
**10**	**CLCA4**	Calcium-activated chloride channel regulator 4		Hydrolase Metalloprotease Protease	Transport
**11**	**SPINK5**	Serine protease inhibitor Kazal-type 5	Secreted	Serine Protease Inhibitor	-
**12**	**LTF**	Lactotransferrin	Secreted Cytoplasm Nucleus	Antibiotic Antimicrobial Hydrolase Serine Protease	Immunity Iron transport Osteogenesis Transcription Regulation
**13**	**SPRR3**	Small proline-rich protein 3	Cytoplasm	Structure Molecule	Epidermis Development Keratinocyte Differentiation
**14**	**TGM3**	Protein-glutamine gamma-glutamyltransferase E	Cytoplasm	Acyltransferase	Keratinization
**15**	**PRB3**	Basic salivary proline-rich protein 3	Secreted	-	-
**16**	**TMPRSS11E**	Transmembrane protease serine 11E	Membrane Secreted	Hydrolase Serine Protease	Cognition Proteolysis
**17**	**MSMB**	Beta-microseminoprotein	Secreted	-	-
**18**	**RHCG**	Ammonium transporter Rh type C	Membrane	Transmembrane Transporter	Ammonia Transport

**19**	**MUC5B**	Mucin-5B	Secreted	-	-
**20**	**SLURP1**	Secreted Ly-6/uPAR-related protein 1	Secreted	Cytokine	Cell Adhesion
**21**	**PLAC8**	Placenta-specific gene 8 protein	-	Chromatin Binding	-
**22**	**CLIC3**	Chloride intracellular channel protein 3	Nucleus Membrane Cytoplasm	Chloride Channel Voltage-gated Channel	Ion Transport
**23**	**KRT24**	Keratin, type I cytoskeletal 24	Cytoskeleton Nucleus	Structure Molecule	Intermediate Filament
**24**	**CRCT1**	Cysteine-rich C-terminal protein 1	Cytoplasm	Structure Molecule	Keratinocyte Differentiation
**25**	**TCN1**	Transcobalamin-1	Secreted	-	Cobalt Transport
**26**	**AZGP1**	Zinc-alpha-2-glycoprotein	Secreted	Protein Transmembrane Transporter	Antigen Processing and Presentation
**27**	**HOPX**	Homeodomain-only protein	Nucleus Cytoplasm	Developmental Protein Repressor	Transcription Regulation
**28**	**CEACAM5**	Carcinoembryonic antigen-related cell adhesion molecule 5	Membrane	GPI Anchor	Cell Adhesion
**29**	**DMBT1**	Deleted in malignant brain tumors 1 protein	Secreted	Developmental Protein	Antiviral Defense Differentiation Protein Transport
**30**	**TF**	Serotransferrin	Secreted	-	Iron Transport

Tab. S6 – Top 30 list of significantly up-regulated genes in M*vs.*PT (log_2FC > 1.5, p-value < 0.0001)

No.	Gene	Protein	Localization	Molecular function	Biological process
1	**ADH1B**	Alcohol dehydrogenase 1B	Cytoplasm	Oxidoreductase	Ethanol Oxidation
2	**MS4A1**	B-lymphocyte antigen CD20	Membrane	-	B-Cell Activation
3	**CCL21**	C-C motif chemokine 21	Secreted	Cytokine	Chemotaxis Inflammatory Response
4	**CD52**	CAMPATH-1 antigen	Cell Membrane	-	-
5	**C7**	Complement Component C7	Secreted	-	Complement pathway Cytolysis Innate Immunity
6	**TRBV5-4**	T cell receptor beta variable 5-4	-	-	-
7	**IGHM**	Ig mu chain C region	Cell Membrane Secreted	Antigen Binding	Immunity
8	**SELL**	L-Selectin	Membrane	-	Cell Adhesion
9	**LTB**	Leukotriene B4 Receptor 2	Cell Membrane	G-protein Coupled Receptor Transducer	Chemotaxis
10	**PTPRC**	Receptor-type Tyrosine-protein Phosphatase C	Membrane	Hydrolase Protein Phosphatase	Immunity
11	**CCL19**	C-C motif Chemokine 19	Secreted	Cytokine	Chemotaxis Inflammmatory response
12	**CCL2**	C-C motif Chemokine 2	Secreted	Cytokine	Chemotaxis Inflammmatory response
13	**FAIM3**	Fas Apoptotic Inhibitory Molecule 3	Membrane Secreted	-	Immunity
14	**TRBC1**	T-Cell Receptor Beta-1 Chain C Region	Membrane	Receptor	Immunity
15	**CCR7**	C-C Chemokine Receptor Type 7	Cell Membrane	G-protein Coupled Receptor Transducer	Activation of GTPase Activity Cellular Response to Cytokine Stimulus
16	**PAX5**	Paired Box Protein Pax-5	Nucleus	Developmental Protein	Differentiation Neurogenesis Transcription Regulation
17	**CD48**	CD48 Antigen	Cell Membrane	Receptor	Blood Coagulation Leukocyte Migration
18	**CD37**	Leukocyte Antigen CD37	Membrane	-	-
19	**ARHGAP15**	Rho GTPase-activating prot. 15	Cytoplasm Membrane	GTPase activation	Regulation of Cell Shape

**20**	**CD69**	Early Activation Antigen CD69	Membrane	Receptor	Cellular Response to Drug
**21**	**CORO1A**	Coronin-1A	Cytoplasm Cytoskeleton Cytoplasmic Vesicle Membrane	-	Cytoskeleton Organisation
**22**	**IRF8**	Interferon Regulatory Factor 8	Nucleus	-	Transcription Regulation
**23**	**LPL**	Lipoprotein Lipase	Cell Membrane Secreted	Hydrolase	Lipid Degradation
**24**	**CXCR4**	C-X-C Chemokine Receptor Type 4	Cell Junction Cell Membrane	G-protein Coupled Receptor Transducer	Host-Virus Interaction
**25**	**GMFG**	Glia Maturation Factor Gamma	Intracellular	Growth Factor	-
**26**	**PRKCB**	Proteinkinase C Beta Type	Cytoplasm Membrane Nucleus	Chromatin Regulator Serine/Threonine-Protein Kinase Transferase	Immunity Apoptosis Transcription Regulation
**27**	**ITK**	Tyrosin-Protein Kinase ITK/TSK	Cytoplasm	Transferase, Tyrosine-Protein Kinase	Immunity
**28**	**ENPP2**	Ectonucleotide Pyrophosphatase Family Member 2	Secreted	Hydrolase	Chemotaxis Lipid Degradation
**29**	**PTGDS**	Prostaglandin-H2 D-Isomerase	Cytoplasm Secreted	Isomerase	Fatty Acid Metabolism Lipid Biosynthesis Prostaglandin Biosynthesis Transport
**30**	**TRDV2**	TRDV2	-	-	-

Tab. S7 – Top 30 list of significantly down-regulated genes in M*vs.*PT (log_2FC < -1.5, p-value < 0.0001)

No.	Gene	Protein	Localization	Molecular function	Biological process
**1**	**DST**	Dystonin	Cyctoskeleton<br>Membrane<br>Cytoplasm<br>Nucleus	Muscle protein	Cell adhesion
**2**	**SERPINB4**	Serpin B4	Cytoplasm	Serine Protease Inhibitor	-
**3**	**FXYD3**	FXYD domain-containing ion transport regulator 3	Membrane	Chloride Channel	Ion Transport
**4**	**TFAP2A**	Transcription factor AP-2-alpha	Nucleus	Activator	Transcription Regulation
**5**	**PERP**	p53 apoptosis effector related to PMP-22	Membrane	-	Apoptosis<br>Cell Adhesion
**6**	**TRIM29**	Tripartite motif-containing protein 29	Cytoplasm	-	Transcription Regulation
**7**	**KLF5**	Krueppel-like factor 5	Nucleus	Activator	Transcription Regulation
**8**	**CDH1**	Cadherin-1	Membrane	-	Cell adhesion
**9**	**CD24**	Signal transducer CD24	Membrane	GPI Anchor	-
**10**	**DSP**	Desmoplakin	Cytoskeleton<br>Membrane<br>Cytoplasm	-	Cell adhesion
**11**	**SCNN1A**	Amiloride-sensitive sodium channel subunit alpha	Membrane	Sodium Channel	Sodium Transport<br>Taste
**12**	**IRF6**	Interferon regulatory factor 6	Cytoplasm<br>Nucleus	Activator	Differentiation<br>Transcription Regulation
**13**	**PPAP2C**	Phospholipid phosphatase 2	Membrane	Hydrolase	Biosynthesis
**14**	**RAB25**	Ras-related protein Rab-25	Membrane	-	Protein Transport
**15**	**CXADR**	Coxsackievirus and adenovirus receptor	Membrane<br>Secreted	Host cell receptor for virus entry	Cell adhesion<br>Host-virus interaction
**16**	**NEBL**	Nebulette	Cytoplasm	-	-
**17**	**MAP7**	Ensconsin	Cytoskeleton<br>Membrane<br>Cytoplasm	-	-
**18**	**TPD52L1**	Tumor protein D53	Cytoplasm	-	-
**19**	**ESRP1**	Epithelial splicing regulatory protein 1	Nucleus	-	mRNA Splicing
**20**	**WNT5A**	Protein Wnt-4	Secreted	Developmental Protein	Wnt Signaling Pathway
**21**	**C1orf106**	Uncharacterized protein C1orf106	-	-	-
**22**	**TUFT1**	Tuftelin	Secreted	-	Biomineralization

**23**	**ATP1B1**	Sodium/potassium-transporting ATPase subunit beta-1	Membrane	-	Sodium/Potassium Transport Cell Adhesion
**24**	**LAMA3**	Laminin subunit alpha-3	Secreted	Structural Molecule	Cell Adhesion
**25**	**FERMT1**	Fermitin family homolog 1	Cytoskeleton Membrane Cytoplasm	-	Cell Adhesion
**26**	**TMEM30B**	Cell cycle control protein 50B	Membrane	-	Lipid Transport
**27**	**FGFR2**	Fibroblast growth factor receptor 2	Membrane Secreted	Tyrosin-protein Kinase Receptor	Diverse
**28**	**GALNT3**	Polypeptide N-acetylgalactosaminyltransferase 3	Membrane Golgi-Apparatus	Glycosyltransferase	Phosphate Homeostasis
**29**	**EXPH5**	Exophilin-5	Endosome	-	-
**30**	**ERBB3**	Receptor tyrosine-protein kinase erbB-3	Membrane Secreted	Tyrosin-protein Kinase Receptor	Diverse

Tab S8: Sel Genes PTvs.N

Gene	log_2FC	P-Value	Protein	Localization	Molecular function	Biological process	Ligand	Reactome
**ASPN**	2,7333	6,72E-06	Asporin	Secreted<br>Extracellular space<br>Extracellular matrix	Calcium Ion Binding	Mineralization	Calcium	ECM proteoglycans
**CDH11**	2,1314	7,13E-05	Cadherin 11,Type 2 (OSF-4)	Cell membrane	Calcium Ion Binding	Cell Adhesion	Calcium<br>Metal-binding	Adherens junctions interactions
**COL1A1**	2,7697	7,79E-07	Collagen, Type I, Alpha 1	Secreted<br>Extracellular space<br>Extracellular matrix	Metal Ion Binding	-	Calcium<br>Metal-binding	ECM Organisation; Integrin and Non-Integrin Membrane-ECM Interactions;
**COL1A2**	2,0748	2,74E-05	Collagen, Type I, Alpha 2	Secreted<br>Extracellular space<br>Extracellular matrix	Metal Ion Binding	-	Calcium<br>Metal-binding	ECM Organisation; Integrin and Non-Integrin Membrane-ECM Interactions;
**COL3A1**	2,0382	4,13E-06	Collagen, Type III, Alpha 1	Secreted<br>Extracellular space<br>Extracellular matrix	Metal Ion Binding	-	Calcium<br>Metal-binding	ECM Organisation; Integrin and Non-Integrin Membrane-ECM Interactions; Signaling by PDGF
**COL4A1**	1,9220	1,58E-08	Collagen, Type IV, Alpha 1	Secreted<br>Extracellular space<br>Extracellular matrix<br>Basement membrane	Metal Ion Binding	Angiogenesis	Calcium<br>Metal-binding	ECM Organisation; Integrin and Non-Integrin Membrane-ECM Interactions; Signaling by PDGF;
**COL4A2**	1,7942	1,34E-07	Collagen, Type IV, Alpha 2	Secreted<br>Extracellular space<br>Extracellular matrix<br>Basement membrane	Metal Ion Binding	Angiogenesis	Calcium<br>Metal-binding	ECM Organisation; Integrin and Non-Integrin Membrane-ECM Interactions; Signaling by PDGF;
**COL5A1**	1,9556	3,05E-05	Collagen, Type V, Alpha 1	Secreted<br>Extracellular space<br>Extracellular matrix	Metal Ion Binding	-	Calcium<br>Heparin-binding<br>Metal-binding	ECM Organisation; Integrin and Non-Integrin Membrane-ECM Interactions; Signaling by PDGF;
**COL5A2**	2,4991	8,50E-07	Collagen, Type V, Alpha 2	Secreted<br>Extracellular space<br>Extracellular matrix	Metal Ion Binding	-	Calcium<br>Metal-binding	ECM Organisation; Integrin and Non-Integrin Membrane-ECM Interactions; Signaling by PDGF;
**COL6A3**	1,7597	1,03E-06	Collagen, Type VI, Alpha 3	Secreted<br>Extracellular space<br>Extracellular matrix	Serine Protease Inhibitor	-	Cell Adhesion	ECM Organisation; Integrin Cell Surface Interactions; Signaling by PDGF;
**COL10A1**	1,7102	3,33E-06	Collagen, Type X, Alpha 1	Secreted<br>Extracellular space<br>Extracellular matrix	Metal Ion Binding	-	Calcium<br>Metal-binding	Integrin and Non-Integrin Membrane-ECM Interactions
**COL11A1**	2,2924	5,46E-05	Collagen, Type XI, Alpha 1	Secreted<br>Extracellular space<br>Extracellular matrix	Metal Ion Binding	-	Calcium<br>Metal-binding	Non-Integrin Membrane-ECM Interactions
**FAP**	1,6717	9,01E-05	Fibroblast Activation Protein Alpha	Cell membrane	Hydrolase<br>Serine Protease	Angiogenesis<br>Apoptosis<br>Cell Adhesion	-	
**FN1**	1,8254	6,56E-06	Fibronectin 1	Secreted	Acute Phase	-	Heparin-Binding	Degradation of the extracellular matrix; Integrin

				Extracellular space<br>Extracellular matrix	Angiogenesis<br>Cell Adhesion<br>Cell Shape			cell surface interactions; MAPK signaling for integrins
**HOXB7**	1,5093	7,80E-09	Homeobox B7	Nucleus	Developmental protein	Transcription<br>Transcription Regulation	DNA-Binding	
**IGFBP3**	1,6046	7,30E-05	Insulin-Like Growth Factor Binding Protein 3	Secreted<br>Nucleus	Apoptosis	Growth factor Binding		Regulation of IGF transport and uptake by IGFBPs
**INHBA**	2,7222	4,22E-07	Inhibin, Beta A	Secreted	Growth Factor<br>Hormone	-	-	Signaling by Activin
**LAMC2**	1,9688	2,06E-05	Laminin, Gamma 2	Secreted<br>Extracellular space<br>Extracellular matrix<br>Basement membrane	Heparin-Binding	Cell Adhesion	Heparin-Binding	Degradation of ECM; Non-integrin membrane-ECM interactions; Anchoring Fibril Formation
**LOX**	1,8267	3,43E-05	Lysyl Oxidase	Secreted<br>Extracellular space	Oxidoreductase		Metal-Binding	Crosslinking of Collagen Fibrils; Elastic Fibre Formation
**LOXL2**	1,7868	8,94E-08	Lysyl Oxidase-like 2	Secreted<br>Extracellular space<br>Extracellular matrix<br>Basement membrane<br>Nucleus	Chromatin Regulator<br>Oxidoreductase<br>Repressor	Transcription Regulation	Metal-Binding	Crosslinking of Collagen Fibrils; Elastic Fibre Formation
**LRP12**	1,5531	1,18E-06	Low Density Lipoprotein Receptor-Related Protein 12	Membrane	Receptor	Endocytosis<br>Host-virus interaction		Retinoid metabolism and transport
**MAGEA4**	1,7378	5,33E-06	Melanoma Antigen Family A, 4	-	-	-	-	-
**MFAP2**	1,6469	3,23E-07	Microfibrillar-Associated Protein 2	Secreted<br>Extracellular space<br>Extracellular matrix	-	ECM organization		Elastic fibre formation
**MICAL2**	1,5042	9,79E-07	Microtubule Associated Monooxygenase, Calponin and LIM Domain containing 2	Cytoplasm (Cytoskeleton)	Monooxygenase<br>Oxidoreductase	Actin-binding<br>FAD<br>Metal-binding<br>NADP		Cytoskeleton Organization; Oxidation-Reduction Process
**MMP1**	4,8582	2,38E-09	Matrix Metallopeptidase 1	Secreted<br>Extracellular space<br>Extracellular matrix	Hydrolase, Metalloprotease	Collagen degradation<br>Host-virus interaction	Calcium<br>Metal-binding<br>Zinc	EC No. 3.4.24.7.; Degradation of ECM; Collagen degradation; Regulation of IGF transport and uptake
**MMP3**	2,7054	2,00E-07	Matrix Metallopeptidase 3	Secreted<br>Extracellular space<br>Extracellular matrix	Hydrolase, Metalloprotease	Collagen degradation	Calcium<br>Metal-binding<br>Zinc	Degradation of ECM, Collagen degradation, Assembly of collagen fibrils, EGFR transactivation
**MMP13**	2,3693	6,55E-07	Matrix Metallopeptidase 13	Secreted<br>Extracellular space<br>Extracellular matrix	Hydrolase, Metalloprotease	Collagen degradation	Calcium<br>Metal-binding<br>Zinc	EC No. 3.4.24.x.; Degradation of ECM; Collagen degradation; Assembly of Collagen fibrils

**NREP**	1,5573	2,80E-07	Neuronal Regeneration Related Protein	Cytoplasm	-	-	-	Regulation of TGFR signaling
**PLAU**	1,5493	9,87E-06	Plasminogen Activator, Urokinase	Secreted	Hydrolase<br>Serine Protease	Blood coagulation<br>Fibrinolysis<br>Hemostasis<br>Plasminogen activation		EC No. 3.4.21.73.; Dissolution of Fibrin Clot
**POSTN**	3,0609	9,40E-09	Periostin, Osteoblast Specific Factor	Secreted<br>Extracellular space<br>Extracellular matrix	Cell adhesion	Heparin-binding		ECM organization, Regulation of Notch signaling, Cell adhesion, tissue development
**PXDN**	1,5851	2,84E-05	Peroxidasin Homolog (Drosophila)	Secreted<br>Extracellular space<br>Extracellular matrix	Oxidoreductase<br>Peroxidase	Hydrogen peroxide	Calcium<br>Heme<br>Iron<br>Metal-binding	EC No. 1.11.1.7
**SCHIP1**	1,5408	3,04E-06	Schwannomin interacting protein 1	Cytoplasm	-	-	-	-
**SERPINE1**	1,6819	5,97E-05	Serpin Peptidase Inhibitor	Secreted	Protease Inhibitor<br>Serine Protease Inhibitor	-	-	Dissolution of Fibrin Clot, ECM proteoglycans; regulation of transcription
**SERPINH1**	1,6014	2,01E-07	Serpin Peptidase Inhibitor Clade H	Secreted	Chaperone	Stress response	-	Collagen biosynthesis and modifying enzymes
**SPARC**	1,6408	3,77E-07	Osteonectin	Secreted<br>Extracellular space<br>Extracellular matrix	-	Calcium<br>Copper<br>Metal-binding	-	ECM proteoglycans
**SPP1**	3,2720	2,01E-05	Osteopontin	Secreted	Cytokine	Biomineralization<br>Cell adhesion	Sialic acid	Degradation of the extracellular matrix; Integrin cell surface interactions; Signaling by PDGF
**SULF1**	1,6696	6,12E-05	Sulfatase1	Cell Surface	Hydrolase	Hydrolase	Calcium<br>Metal-binding	-
**TFRC**	1,5982	3,76E-06	Transferrin Receptor (CD71)	Cell membrane<br>Secreted	Receptor	Endocytosis<br>Host-virus interaction	-	Golgi Associated Vesicle Biogenesis; Transferrin endocytosis and recycling

Tab S9: Sel Genes Mvs.PT

Gene	log_2FC	P-Value	Protein	Localization	Molecular function	Biological process	Ligand	Reactome
**ADH1B**	3,02	3,04E-06	Alcohol Dehydrogenase 1B	Cytoplasm	Oxidoreductase	Ethanol Oxidation	Metal-binding<br>NAD<br>Zinc	EC No. 1.1.1.1; Xenobiotic Metabolic Process
**APOE**	1,62	2,96E-07	Apolipoprotein E	Secreted	Antioxidant Activity	Cholesterol-/Lipid-/Steroid-Metabolism<br>Lipid Transport	Heparin-binding	HDL-mediated Lipid Transport, Chylomicron-mediated Lipid Transport; Retinoid Metabolism and Transport
**ARHGAP15**	2,12	1,40E-07	Rho GTPase-activating prot. 15	Cytoplasm<br>Membrane	GTPase activation	Regulation of Cell Shape	-	Rho GTPase Cycle
**C7**	2,47	2,14E-08	Complement Component C7	Secreted	-	Complement pathway<br>Cytolysis<br>Innate Immunity	-	Regulation of Complement Cascade; Terminal pathway of Complement
**CCL19**	2,34	4,73E-06	C-C motif Chemokine 19	Secreted	Cytokine	Chemotaxis<br>Inflammmatory response	-	Chemokine Receptors bind Chemokines, G alpha(i) Signaling Events
**CCL2**	2,29	5,51E-05	C-C motif Chemokine 2	Secreted	Cytokine	Chemotaxis<br>Inflammmatory response	-	Chemokine Receptors bind Chemokines, ATF4 activates Genes
**CCL21**	2,70	6,60E-07	C-C motif Chemokin 21	Secreted	Cytokine	Chemotaxis<br>Inflammmatory response	-	Chemokine Receptors bind Chemokines, G alpha(i) Signalling Events
**CCR7**	2,22	2,56E-05	C-C Chemokine Receptor Type 7	Cell Membrane	G-protein Coupled Receptor<br>Transducer	Activation of GTPase Activity<br>Cellular Response to Cytokine Stimulus	-	Chemokine Receptors bind Chemokines, G alpha(i) Signalling Events
**CD22**	1,52	3,27E-06	B-cell receptor CD22	Cell Membrane	Carbohydrate-binding	Cell Adhesion	Lectin	-
**CD37**	2,13	1,05E-05	Leukocyte Antigen CD37	Membrane	-	-	-	Regulation of Cell Proliferation, Regulation of Humoral Immune Response, Regulation of Immunogolbulin Production
**CD48**	2,17	1,49E-06	CD48 Antigen	Cell Membrane	Receptor	Blood Coagulation<br>Leukocyte Migration	-	Cell Surface Interactions at the Vascular Wall
**CD52**	2,58	3,47E-07	CAMPATH-1 antigen	Cell Membrane	-	-	-	Regulation of Cytosolic Calcium Ion Concentration, Respiratory Burst
**CD53**	1,84	7,22E-07	Leukocyte Antigen CD53	Cell Junction<br>Cell Membrane	-	-	-	Regulation of Myoblast Function, Signal Transduction
**CD69**	2,10	2,17E-05	Early Activation Antigen CD69	Membrane	Receptor	Cellular Response to Drug	Calcium<br>Lectin	Signal Transduction
**CD209**	1,58	5,31E-07	CD209 antigen	Cell Membrane<br>Secreted	Receptor	Immunity<br>Cell Adhesion<br>Endocytosis	Calcium,Lectin<br>Mannose-binding<br>Metal-binding	CD209 (DC-SIGN) signaling
**CD3D**	1,91	2,01E-05	T-cell surface glycoprotein CD3	Membrane	Receptor	T-cell Regulation	-	Immunoregulatory Interactions between a Lymphoid and a non-Lymphoid Cell, TCR signaling, PD-1

			delta chain					Signaling
**CORO1A**	2,09	5,86E-06	Coronin-1A	Cytoplasm<br>Cytoskeleton<br>Cytoplasmic Vesicle<br>Membrane	-	Cytoskeleton Organisation	Actin-binding<br>Myosin-binding<br>PI3K-binding	Movement of Cell or Subcellular Component
**CXCL12**	1,92	6,23E-05	Stromal Cell-derived Factor 1	Secreted	Cytokine<br>Growth Factor	Chemotaxis	-	Nuclear Signalling by ERBB4, Chemokine Binding, G alpha (i) Signalling Events
**CXCR4**	1,97	6,04E-05	C-X-C Chemokine Receptor Type 4	Cell Junction<br>Cell Membrane	G-protein Coupled Receptor<br>Transducer	Host-Virus Interaction	-	Chemokine Binding, G alpha (i) Signaling Events
**CYFIP2**	1,57	3,33E-06	Cytoplasmic FMR1-interacting Protein 2	Cell Junction<br>Cytoplasm	-	Apoptosis<br>Cell Adhesion	-	VEGFA-VEGFR2 Pathway, Rho GTPase Activates WASPs and WAVEs, Regulation of Actin Dynamics in Phagocytosis
**CYTIP**	1,65	4,14E-05	Cytohesin-interacting Protein	Cytoplasm<br>Endosome	-	Cell Adhesion	-	Activation of ARFs by CYTH1
**DNASE1L3**	1,56	5,83E-05	Deoxyribonuclease Gamma	Nucleus	Endonuclease<br>Hydrolase<br>Nuclease	Apoptosis	Calcium	Apoptotic DNA Fragmentation, Developmental Programmed Cell Death
**DOCK2**	1,81	8,53E-07	Dedicator of Cytokinesis Protein 2	Cytoplasm<br>Cytoskeleton<br>Membrane	Guanine-nucleotide Releasing Factor	Cytoskeleton Organisation<br>Chemotaxis<br>Reg. of GTPase Activity	-	Nef and Signal Transduction, Factors Involved in Megakaryocyte Development and Platelet Production
**ENPP2**	1,90	1,05E-05	Ectonucleotide Pyrophosphatase Family Member 2	Secreted	Hydrolase	Chemotaxis<br>Lipid Degradation	Calcium<br>Metal-binding<br>Zinc	EC No. 3.1.4.39; LPA Production in Extracellular Fluids, Modulation of Cell Motility
**EVI2B**	1,54	6,94E-05	Protein EVI2B	Membrane	-	-	-	-
**FAIM3**	1,86	1,49E-05	Fas Apoptotic Inhibitory Molecule 3	Membrane<br>Secreted	-	Immunity	-	Protection of Cells from FAS-, TNF alpha- and FADD-induced Apoptosis
**FAM65B**	1,73	2,03E-05	Protein FAM65B	Cell Membrane<br>Cytoskeleton<br>Mitochondrion	Developmental Protein	Differentiation<br>Hearing<br>Myogenesis	-	Cell Differentiation, Muscle Organ Development, Sensory Perception of Sound
**GIMAP6**	1,58	1,30E-05	GTPase IMAP Family Member 6	Cytoplasm	-	-	GTP-binding<br>Nucleotide-binding	-
**GMFG**	2,01	7,13E-08	Glia Maturation Factor Gamma	Intracellular	Growth Factor	-	-	Cytoskeleton Reorganisation, Regulation of Protein Kinase Activity
**GPR183**	1,69	1,73E-06	G-protein Coupled Receptor 183	Cell Membrane	G-protein Coupled Receptor<br>Transducer	Immunity	-	Immune Response, Regulation of ERK1 and ERK2 Cascade
**HMHA1**	1,53	4,43E-06	Minor Histocompatibility Protein HA-1	Cytoplasm<br>Membrane	GTPase Activation	-	Metal-binding<br>Zinc	Rho GTPase Cycle
**ICAM2**	1,77	2,08E-07	Intracellular Adhesion Molecule2	Membrane	-	Cell Adhesion	-	Immunoregulatory Interactions between a Lymphoid and a Non-Lymphoid Cell, Integrin Interactions, CD209

								(DC-SIGN) Signaling
**IGHM**	2,42	1,81E-05	Ig Mu Chain C Region	Cell Membrane Secreted	Antigen-binding	Immunity	-	BCR Activation by Antigen
**IL10RA**	1,64	1,54E-07	Interleukin-10 Receptor Subunit Alpha	Membrane	Receptor	-	-	Cytokine-mediated Signaling Pathway, Response to Lipopolysaccharide
**IL7R**	1,63	3,86E-05	Interleukin-7 Receptor Subunit Alpha	Cell Membrane Secreted	Receptor	-	-	Interleukin-7 Signaling
**IRF8**	2,08	1,52E-06	Interferon Regulatory Factor 8	Nucleus	-	Transcription Regulation	DNA-binding	Interferon Signaling
**ITGB2**	1,56	6,51E-07	Integrin Beta-2	Membrane	Integrin, Receptor	Cell Adhesion	Metal-binding	Immunoregulatory Interactions between a Lymphoid and a non-LymphoidCell, Integrin Interactions, Toll Like Receptor 4 (TLR4) Cascade
**ITK**	1,98	8,38E-06	Tyrosin-Protein Kinase ITK/TSK	Cytoplasm	Transferase, Tyrosine-Protein Kinase	Immunity	ATP-binding Metal-binding Nucleotide-binding Zinc	EC No. 2.7.10.2; Generation of Second Messenger Molecules, FCERI mediated Ca2+ Mobilisation
**ITM2A**	1,50	6,82E-05	Integral Membrane Protein 2A	Membrane	-	-	-	-
**KLF2**	1,86	8,83E-08	Krueppel-Like Factor 2	Nucleus	Activator	Transcription Regulation	DNA-binding Metal-binding Zinc	Activation of Beta-Globin Transcription
**LAPTM5**	1,73	8,94E-07	Lysosomal-associated Transmembrane Protein 5	Lysosome Membrane	-	Transport	-	Role in Embryogenesis and in Hematopoietic Cells
**LPL**	2,04	1,86E-05	Lipoprotein Lipase	Cell Membrane Secreted	Hydrolase	Lipid Degradation	Heparin-binding	EC No. 3.1.1.34; Retinoid Metabolism and Transport, Transcriptional Regulation of White Adipocyte Differentiation, Chylomicron-mediated Lipid Transport
**LRMP**	1,62	7,55E-05	Lymphoid-restricted Membrane Protein	Cytoplasm	-	Fertilization Immunity	-	Delivery of Peptides to MHC Class I Molecules, Taste Signal Transduction Via ITPR3, Pronucleus Congression and Fusion
**LTB**	2,38	8,21E-07	Leukotriene B4 Receptor 2	Cell Membrane	G-protein Coupled Receptor, Transducer	Chemotaxis	-	G alpha (q) Signaling Events, Leukotriene Receptors
**LYVE1**	1,67	8,50E-06	Lymphatic Vessel Endothelial Hyaluronic Acid Receptor 1	Membrane	Receptor	Transport	-	Hyaluronan Uptake and Degradation
**MS4A1**	2,80	8,83E-05	B-Lymphocyte CD20	Cell Membrane	-	B-Cell Activation	-	Regulation of B-Cell Activation and Proliferation
**PAX5**	2,21	7,71E-05	Paired Box Protein Pax-5	Nucleus	Developmental Protein	Differentiation Neurogenesis Transcription Regulation	DNA-binding	Immune Response, Nervous System Development, Spermatogenesis, Skeletal Muscle Cell Differentiation, Multicellular Organismal Development

**PLIN1**	1,88	6,42E-05	Perilipin-1	Endoplasmatic Reticulum<br>Lipid Droplet	-	Lipid Metabolism	-	Transcriptional Regulation of White Adipocyte Differentiation, Hormone-sensitive Lipase (HSL)-mediated Tracylglycerol Hydrolysis
**PRKCB**	1,97	1,19E-05	Proteinkinase C Beta Type	Cytoplasm<br>Membrane<br>Nucleus	Chromatin Regulator<br>Serine/Threonine-Protein Kinase<br>Transferase	Immunity<br>Apoptosis<br>Transcription Regulation	ATP-binding<br>Calcium<br>Metal-binding<br>Nucleotide-binding<br>Zinc	EC No. 2.7.11.13; Activation of NF-kappaB in B Cells, G alpha (z) Signalling Events, Depolymerisation of the Nuclear Lamina, VEGR2-mediated Cell Proliferation
**PTGDS**	1,89	1,15E-06	Prostaglandin-H2 D-Isomerase	Cytoplasm<br>Secreted	Isomerase	Fatty Acid Metabolism<br>Lipid Biosynthesis<br>Prostaglandin Biosynthesis<br>Transport	-	Synthesis of Prostaglandins (PG) and Thromboxanes (TX)
**PTPRC**	2,20	8,53E-06	Receptor-type Tyrosine-protein Phosphatase C	Membrane	Hydrolase<br>Protein Phosphatase	Immunity	-	Phosphorylation of CD3 and TCR Zeta Chains, Semaphorin Interactions
**RASSF2**	1,61	2,47E-05	Ras-association Domain-containing Protein 2	Cytoplasm<br>Nucleus	-	Cell Cycle	-	Bone Remodeling, EGFR Signaling via NF-kappaB Cascade, Ossification, Skeletal System Development
**SELL**	2,41	1,37E-05	L-Selectin	Membrane	-	Cell Adhesion	Lectin	Immunoregulatory Interactions Between a Lymphoid and a non-Lymphoid Cell;
**SEPT6**	1,71	7,68E-06	Septin-6	Cytoplasm	-	Cell Cycle, Cell Division	GTP-binding<br>Nucleotide-binding	Cytokinesis, Viral Processes
**SH2D1A**	1,55	4,48E-06	SH2 Domain-containing Prot. 1A	Cytoplasm	-	Immunity	-	Inhibitor of SLAM Self-Association, Mediation of Interaction between FYN and SLAMF1
**SLC2A3**	1,80	1,46E-05	Solute Carrier Family 2, Facilitated Glucose Transporter Member 3	Membrane	-	Sugar Transport	-	Facilitative Na+-independant Glucose Transporters
**SRGN**	1,81	3,05E-05	Serglycin	Secreted	-	Apoptosis<br>Biomineralization	-	Platelet Degranulation
**STAP1**	1,65	8,24E-05	Signal-Transducing Adaptor Protein 1	Cytoplasm<br>Nucleus	-	Immunity	-	BCR Activation by Antigen
**TGFBR2**	1,52	2,43E-05	TGF-beta Receptor Type-2	Cell Membrane	Receptor<br>Serine/Threonine-Protein Kinase<br>Transferase	Apoptosis<br>Differentiation<br>Growth Regulation	ATP-binding<br>Magnesium<br>Metal-binding<br>Nucleotide-binding	EC No. 2.7.10.2; TGF-beta Receptor Signaling in EMT, SMAD2/3 Mutants in Cancer, TGFBR Mutants in Cancer;
**TRAC**	1,78	2,00E-05	T Cell Receptor Alpha Constant	Membrane	Receptor	Immunity	-	Immunoregulatory Interactions between a Lymphoid and a Non-Lymphoid Cell, TCR Signaling, PD-1 Signaling
**TRBC1**	2,24	1,85E-05	T-Cell Receptor Beta-1 Chain C Region	Membrane	Receptor	Immunity	-	Immunoregulatory Interactions between a Lymphoid and a Non-Lymphoid Cell, TCR Signaling, PD-1 Signaling
**YME1L1**	1,65	8,37E-06	ATP-dependant Zinc Metalloprotease YME1L1	Mitochondrion Inner Membrane	Hydrolase<br>Metalloprotease	Mitochondrial Protein Metabolism	ATP-binding<br>Zinc-binding<br>Nucleotide-binding	Maintenance of Cristae Morphology and Complex I Respiration Activity, Protection from Accumulation of Oxidatively Damaged Membrane Proteins

Tab. S10: Top Networks (by Ingenuity Pathway Analysis®) PT*vs.*N

ID	Score	Focus Molecules	Top Diseases and Functions
1	57	27	Cellular Development, Infectious Disease, Cancer
2	47	24	Connective Tissue Disorders, Dermatological Diseases and Conditions, Cellular Assembly and Organization
3	34	19	Molecular Transport, Cellular Growth and Proliferation, Embryonic Development
4	31	17	Embryonic Development, Organismal Development, Tissue Development
5	28	16	Inflammatory Disease, Organismal Injury and Abnormalities, Renal and Urological Disease
6	26	15	Dermatological Diseases and Conditions, Inflammatory Response, Gastrointestinal Disease
7	19	12	Organismal Injury and Abnormalities, Cellular Movement, Haematological System Development and Function
8	17	12	Cellular Function and Maintenance, Small Molecule Biochemistry, Amino Acid Metabolism
9	13	9	Cellular Assembly and Organization, Cellular Function and Maintenance, Cell Death and Survival
10	2	1	Cancer, Gastrointestinal Disease, Hepatic System Disease

Tab. S11: Top Networks (by Ingenuity Pathway Analysis®) M*vs.*PT

ID	Score	Focus Molecules	Top Diseases and Functions
1	38	18	Humoral Immune Response, Protein Synthesis, Haematological System Development and Function
2	27	14	Cellular Movement, Haematological System Development and Function, Immune Cell Trafficking
3	25	14	Cell-To-Cell Signalling and Interaction, Haematological System Development and Function, Tissue Morphology
4	19	11	Cell Cycle, Cell Morphology, Cellular Compromise
5	12	8	Cancer, Dermatological Diseases and Conditions, Gastrointestinal Disease
6	12	8	Lipid Metabolism, Small Molecule Biochemistry, Cardiovascular Disease
7	11	7	Cancer, Endocrine System Disorders, Developmental Disorder
8	2	1	Carbohydrate Metabolism, Cellular Assembly and Organization, Cellular Movement
9	2	1	Cell Morphology, Cellular Assembly and Organization, Cellular Compromise

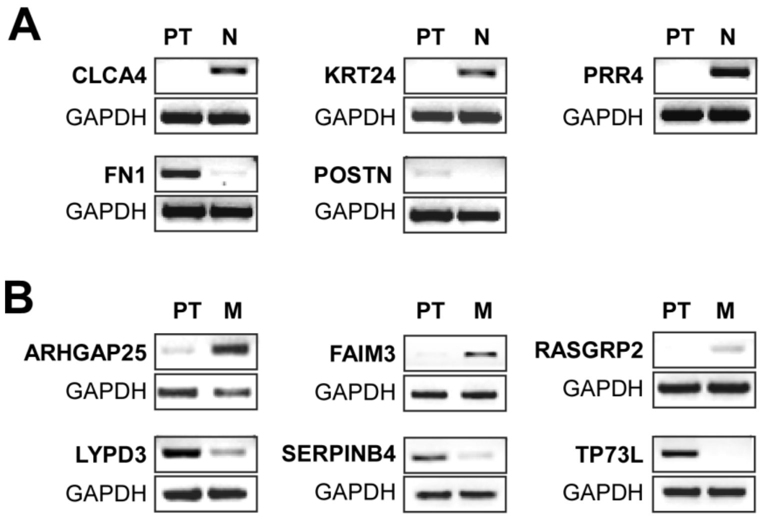

Fig. S1 – Verification of gene expression data obtained by Microarray Analysis via *Affymetrix U133A GeneChip* arrays for selected genes using RT-PCR. For selected representative genes that were significantly up- or downregulated in our respective dataset of PT*vs.*N (A) or M*vs.*PT (B), differential mRNA levels were analysed by RT-PCR.

A. RT-PCR amplification products of genes that are lower expressed (upper row) or higher expressed (lower row) in primary tumours (PT) compared to normal mucosa (N). **B.** RT-PCR amplification products of genes that are lower expressed (upper row) or higher expressed (lower row) in primary tumours (PT) compared metastasis (M). Shown gels are cropped. Complete gels are shown in Supplementary Fig. S30.

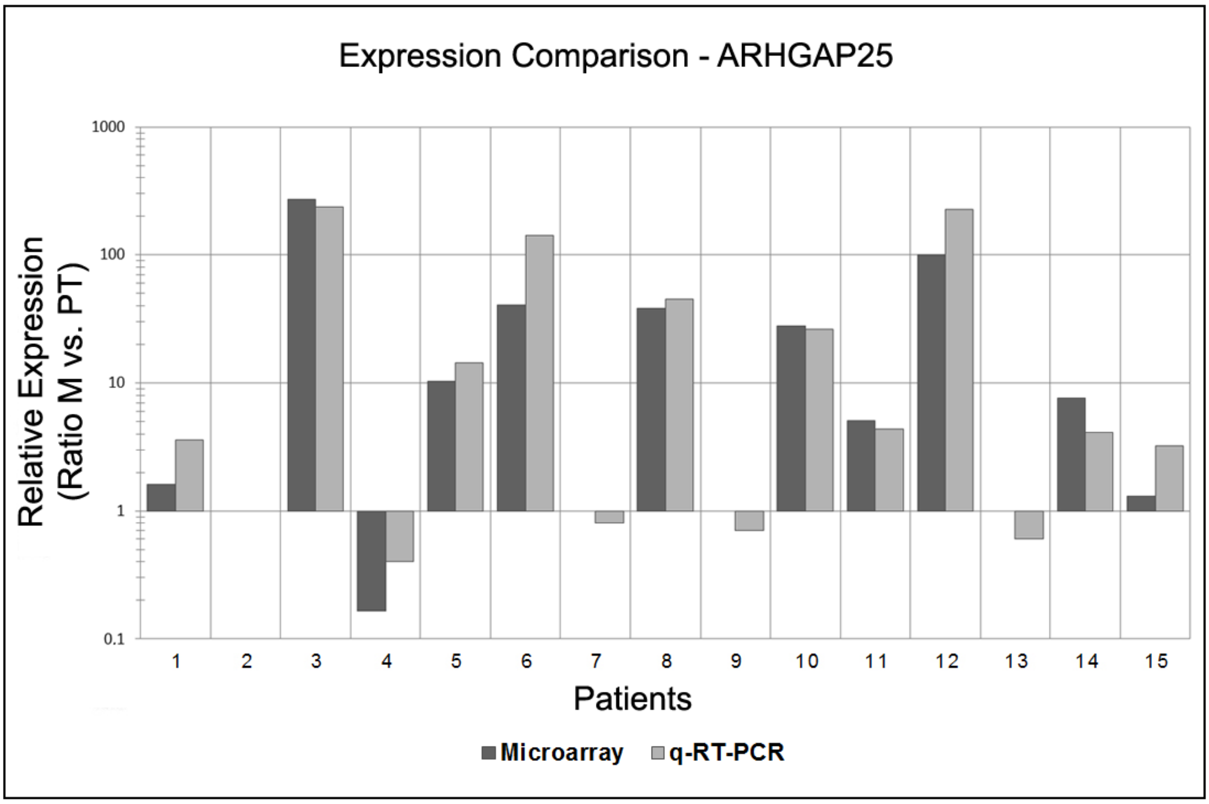

Fig. S2: Comparison of relative gene expression for the gene ARHGAP25. Expression ratios were determined for lymph node metastasis (M) vs. primary tumour (PT) tissue of the indicated patients, using either Affymetrix GeneChip arrays or real-time quantitative PCR analysis. Up-regulation of ARGHAP25 in metastasis was confirmed for most of the patients.

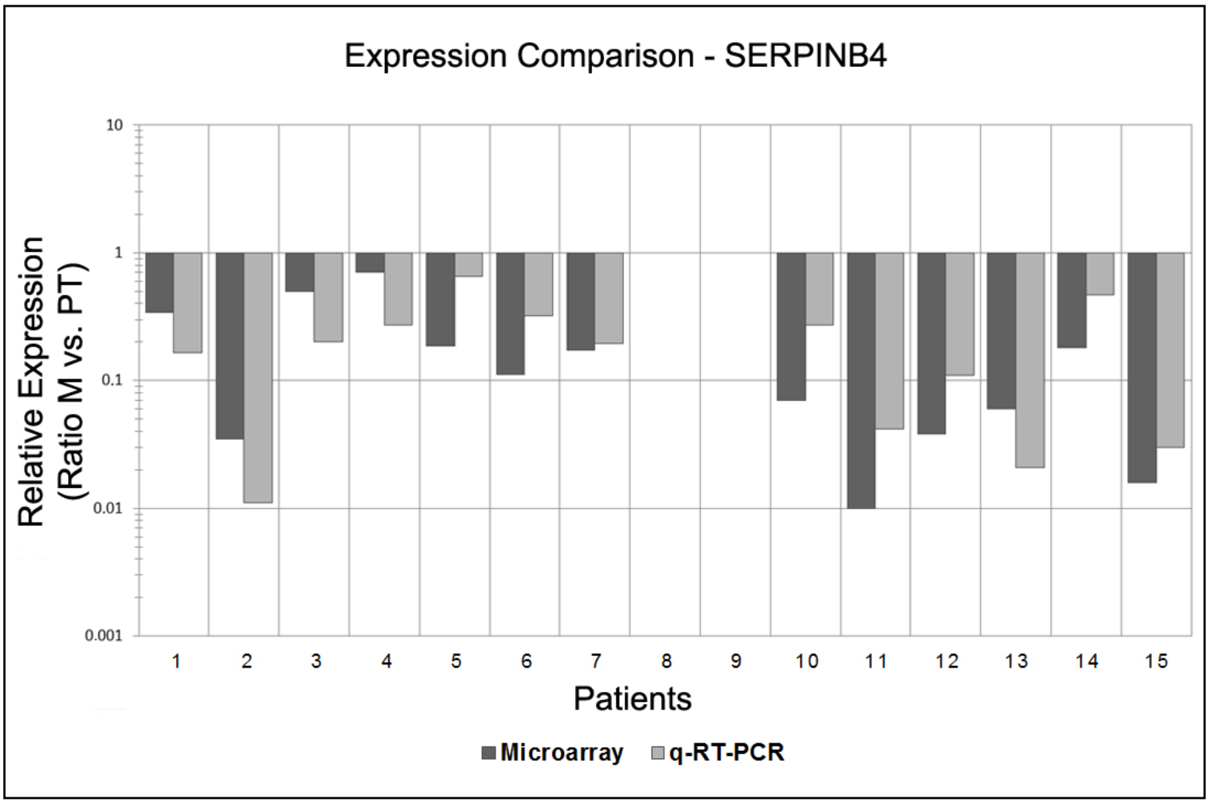

Fig. S3: Comparison of relative gene expression for the gene SERPINB4. Expression ratios were determined for lymph node metastasis (M) vs. primary tumour (PT) tissue of the indicated patients, using either Affymetrix GeneChip arrays or real-time quantitative PCR analysis. Down-regulation of SERPINB4 in metastasis was confirmed for most of the patients.

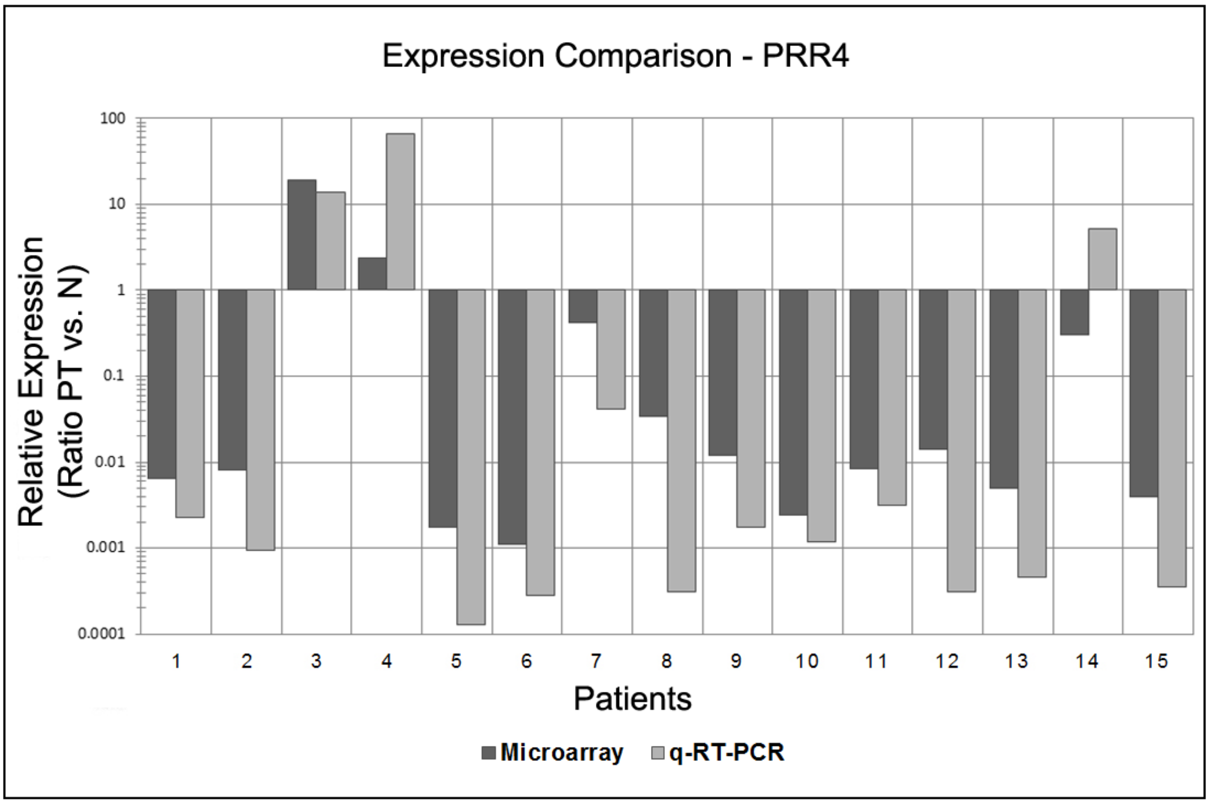

Fig. S4: Comparison of relative gene expression for the gene PRR4. Expression ratios were determined for primary tumour tissue (PT) vs. normal mucosa (N) of the indicated patients, using either Affymetrix GeneChip arrays or real-time quantitative PCR analysis. Down-regulation of PRR4 in primary tumors was confirmed for most of the patients.

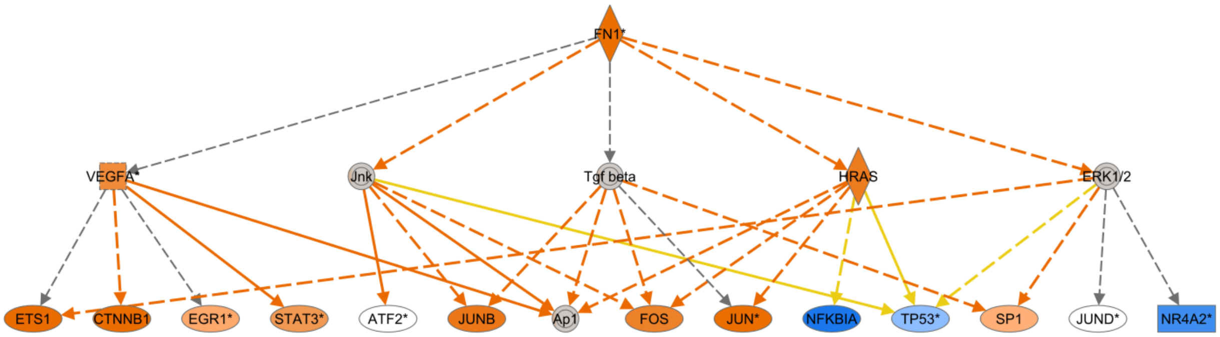

Fig. S5 – Upstream Activator network of FN1 in PT*vs.*N.

Predicted factors influenced by FN1 at a threshold of $\log_2FC > 1.5$, p-value < 0.0001 for genes differentially expressed in primary tumours compared to normal mucosa (PT*vs.*N). Orange: activated. Blue: inactivated. Yellow: findings inconsistent with prediction. Lines and arrows between nodes represent direct (solid lines) and indirect (dashed lines) interactions between molecules. All edges are supported by at least one reference from the literature or from canonical information stored in the Ingenuity Knowledge Base. Node shapes represent functional classes of gene products: square → cytokine, triangle → kinase, rectangle → nuclear receptor, concentric circle → group or complex, vertical diamond → enzyme, horizontal diamond → peptidase, trapezium → transporter, vertical ellipse → transmembrane receptor horizontal ellipse→ transcription regulator, circle → other. See Tab. 1A for reference.

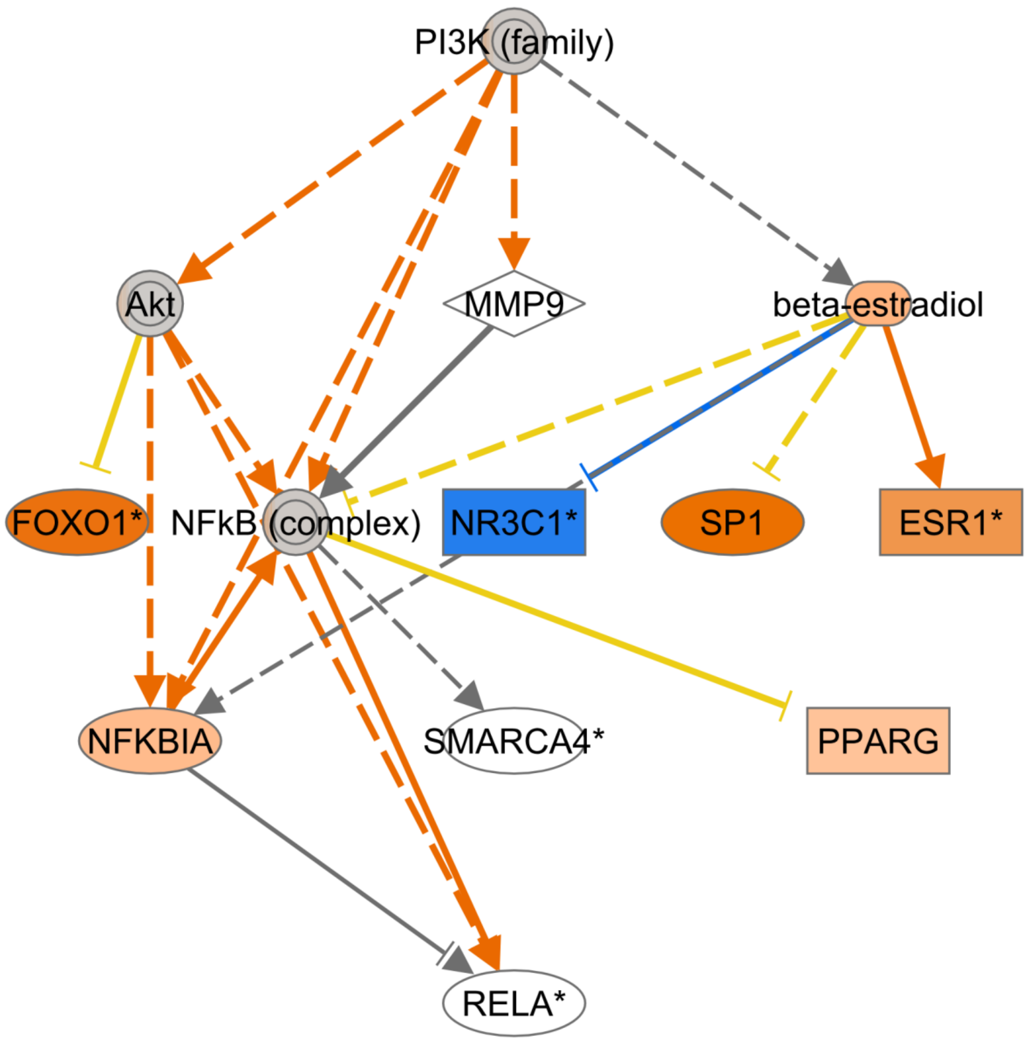

Fig. S6 – Upstream Activator network of PI3K in M*vs.*PT.
Predicted factors influenced by PI3K at a threshold of $log_2FC > 1.5$, p-value < 0.0001 for genes differentially expressed in metastasis compared to primary tumours (M*vs.*PT). Orange: activated. Blue: inactivated. Yellow: findings inconsistent with prediction. Lines and arrows between nodes represent direct (solid lines) and indirect (dashed lines) interactions between molecules. All edges are supported by at least one reference from the literature or from canonical information stored in the Ingenuity Knowledge Base. Node shapes represent functional classes of gene products: square → cytokine, triangle → kinase, rectangle → nuclear receptor, concentric circle → group or complex, vertical diamond → enzyme, horizontal diamond → peptidase, trapezium → transporter, vertical ellipse → transmembrane receptor horizontal ellipse→ transcription regulator, circle → other. See Tab. 1A for reference.

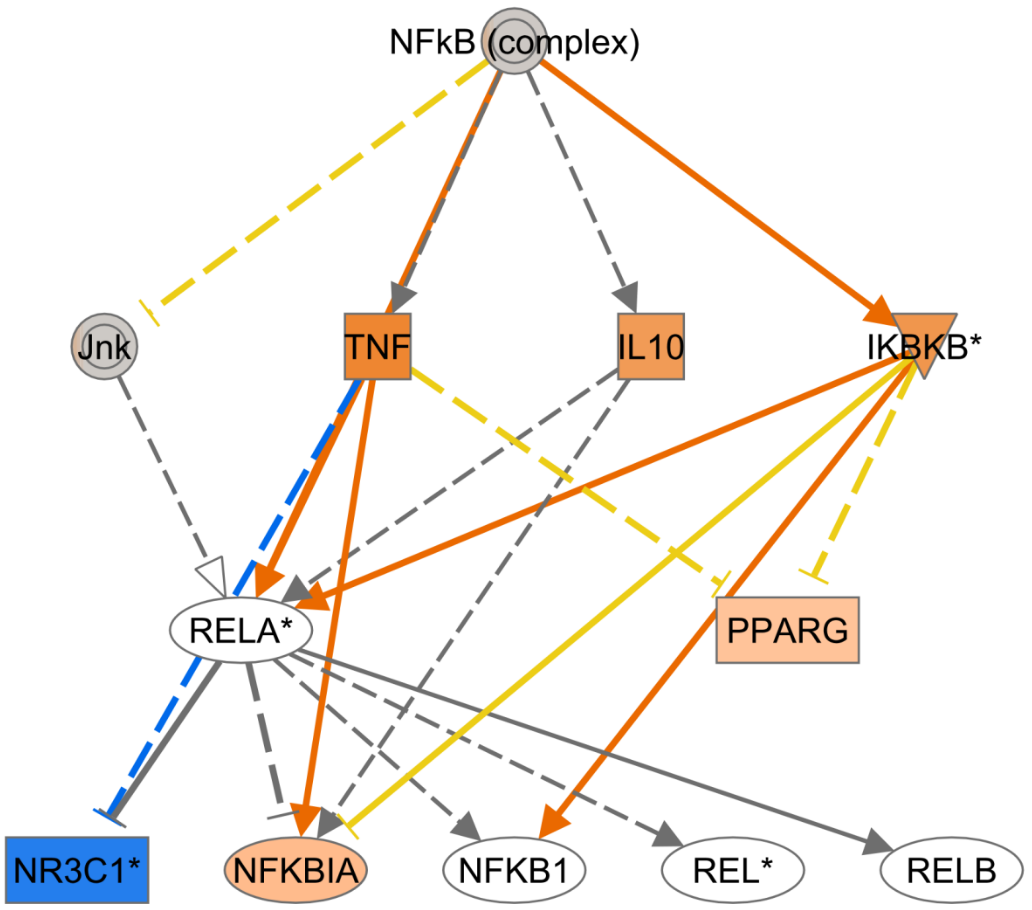

Fig. S7 – Upstream Activator NFkB complex in M*vs.*PT.
Predicted factors influenced by NFkB at a threshold of $log_2FC > 1.5$, p-value < 0.0001 for genes differentially expressed in metastasis compared to primary tumours (M*vs.*PT). Orange: activated. Blue: inactivated. Yellow: findings inconsistent with prediction. Lines and arrows between nodes represent direct (solid lines) and indirect (dashed lines) interactions between molecules. All edges are supported by at least one reference from the literature or from canonical information stored in the Ingenuity Knowledge Base. Node shapes represent functional classes of gene products: square → cytokine, triangle → kinase, rectangle → nuclear receptor, concentric circle → group or complex, vertical diamond → enzyme, horizontal diamond → peptidase, trapezium → transporter, vertical ellipse → transmembrane receptor horizontal ellipse→ transcription regulator, circle → other. See Tab. 1A for reference.

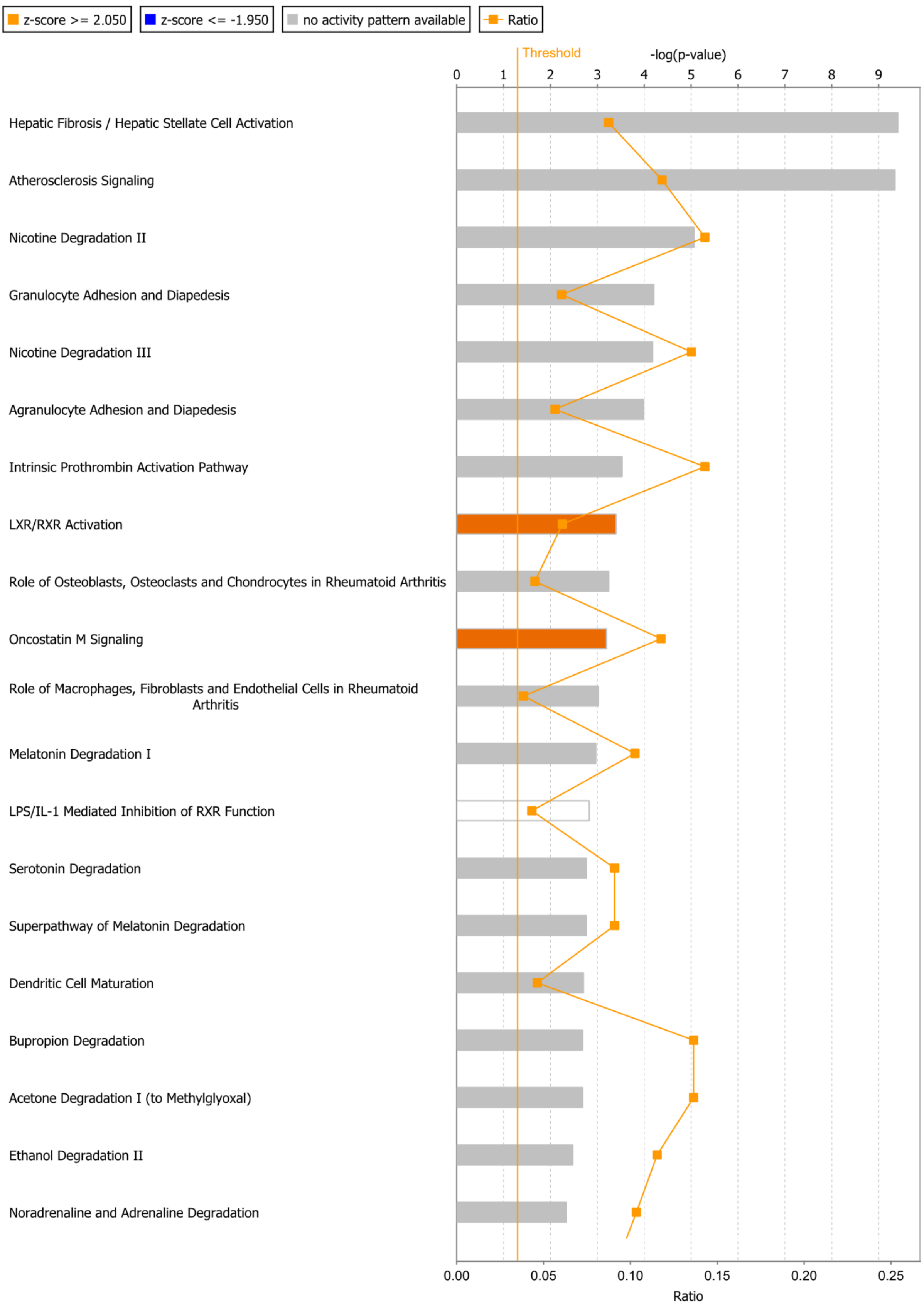

Fig. S8 – Top 20 canonical pathways of the PT*vs.*N dataset as determined by *Ingenuity* analysis.

Comparison analysis of selected top diseases and functions with similar expression analysis at a threshold of $\log_2$FC > 1.5, p-value < 0.0001 for genes differentially expressed in primary tumours compared to normal mucosa (PT*vs.*N). The overlap p-value of the canonical

pathways analysis is shown as –log(p-value) and aids to identify statistical significant overlaps between the observed dataset gene values and the genes that regulate a canonical pathway. The activation z-score compares likely activation states of the canonical pathways with randomly assigned regulation directions, thus allowing to infer on the activation states of the predicted canonical pathways with orange bars indicating pathways being activated, blue bars indicating pathways being inhibited. Ratio indicates the computed difference between genes expressed in primary tumours to normal mucosa for the particular pathway.

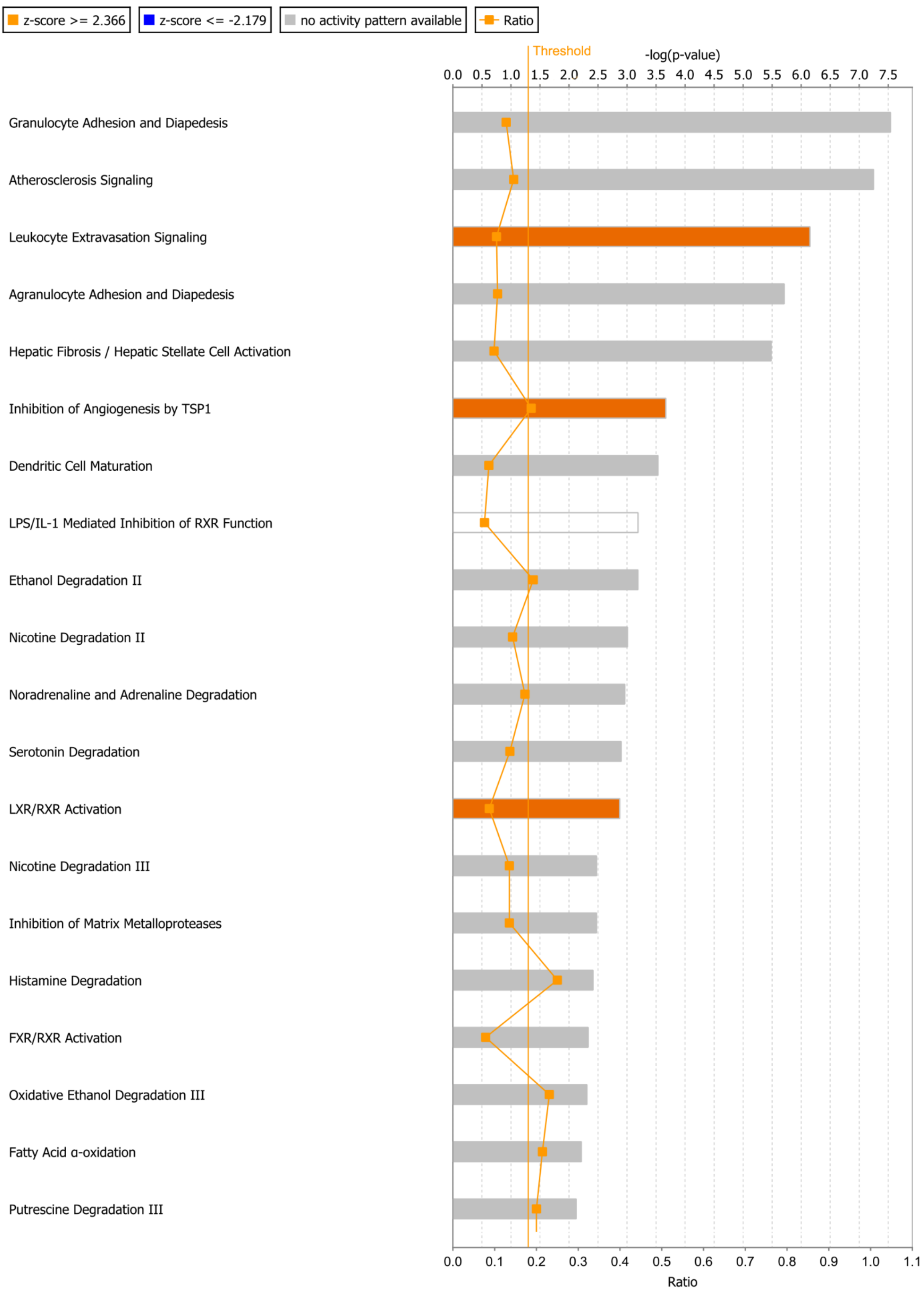

Fig. S9 – Top 20 canonical pathways of the M*vs.*N dataset as determined by *Ingenuity* upstream regulator analysis.
Comparison analysis of selected top diseases and functions with similar expression analysis at a threshold of $\log_2$FC > 1.5, p-value < 0.0001 for genes differentially expressed in lymph

node metastasis compared to primary tumours (PT*vs.*M). The overlap p-value of the canonical pathways analysis is shown as –log(p-value) and aids to identify statistical significant overlaps between the observed dataset gene values and the genes that regulate a canonical pathway. The activation z-score compares likely activation states of the canonical pathways with randomly assigned regulation directions, thus allowing to infer on the activation states of the predicted canonical pathways with orange bars indicating pathways being activated, blue bars indicating pathways being inhibited. Ratio indicates the computed difference between genes expressed in metastasis to primary tumours for the particular pathway.

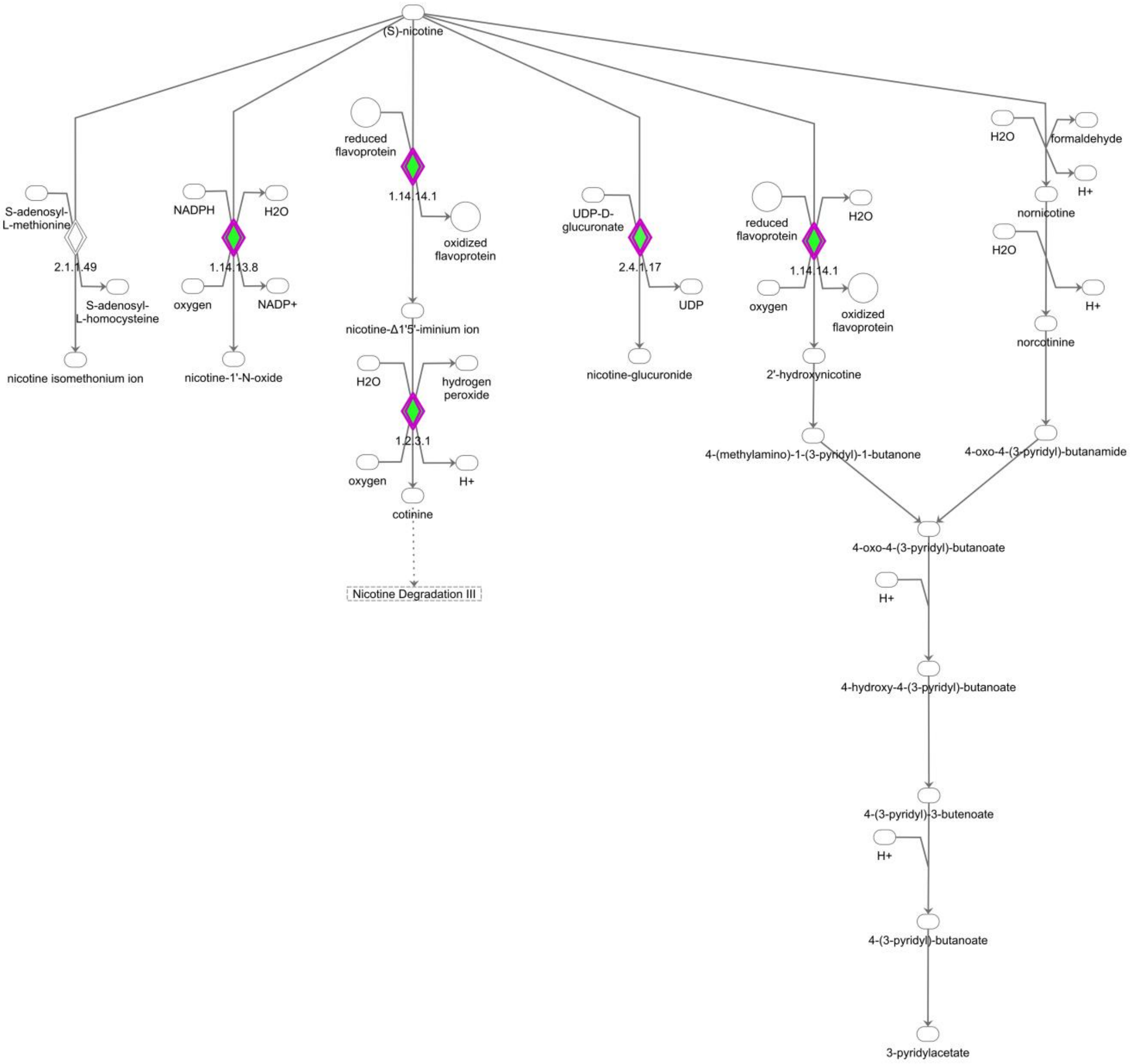

Fig. S10 – Activated and upregulated canonical pathway: Nicotine Degradation II. Almost all enzymes (with the exception of 2.1.1.49) involved in the degradation of nicotine within this pathway are activated and significantly upregulated in PT*vs.*N as well as M*vs.*PT. Green rhombi mark the upregulated enzymes, the purple borders mark the activated steps of the pathway.

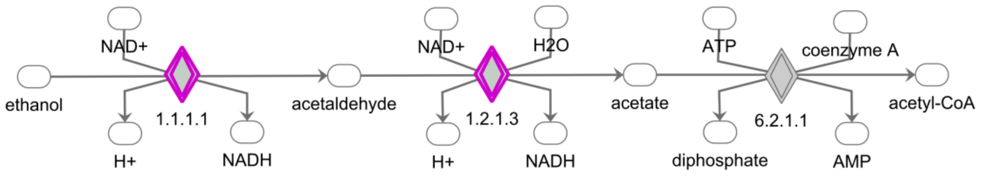

Fig. S11 – Activated canonical pathway: Ethanol Degradation II. Almost all enzymes (with the exception of 6.2.1.1) involved in the degradation of ethanol within this pathway are activated in PT*vs.*N as well as M*vs.*PT. Purple borders mark the activated steps of the pathway.

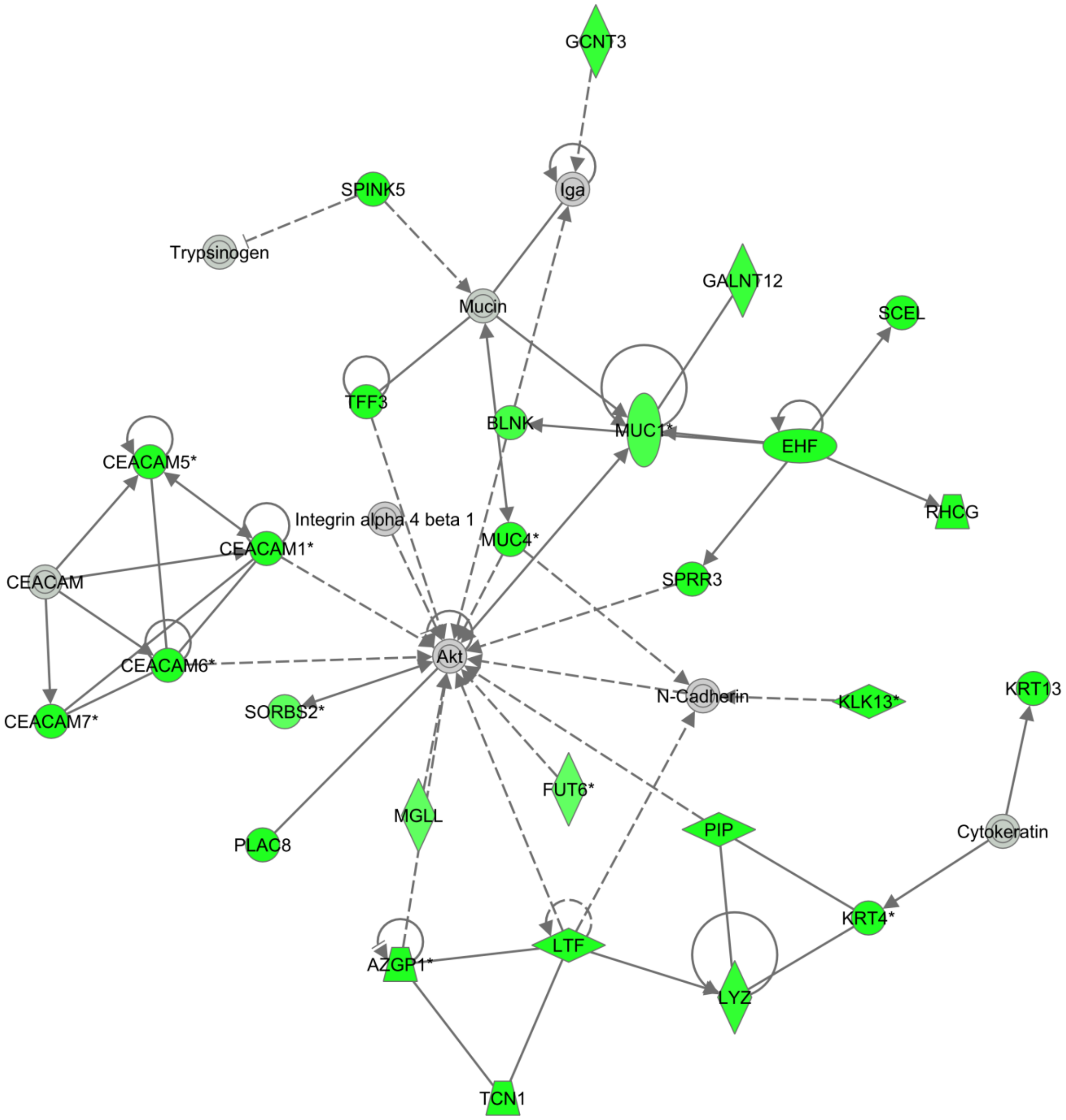

Fig. S12: Top 01 network of differentially expressed genes in PT*vs.*N as identified by Ingenuity Pathway Analysis show strong association to cellular development, infectious disease and cancer, containing 27 focus molecules. For network analyses a threshold of $\log_2 FC > |1.5|$ with a p-value < 0.0001 was used, giving an overview of involved genes within the networks and their differential expression. Node colour indicates up-regulated (red), down-regulated (green) or not significantly changed according to threshold (grey) genes. Lines and arrows between nodes represent direct (solid lines) and indirect (dashed lines) interactions between molecules. All edges are supported by at least one reference from the literature or from canonical information stored in the Ingenuity Knowledge Base. Node shapes represent functional classes of gene products: square → cytokine, triangle → kinase, rectangle → nuclear receptor, concentric circle → group or complex, vertical diamond → enzyme, horizontal diamond → peptidase, trapezium → transporter, vertical ellipse → transmembrane receptor horizontal ellipse→ transcription regulator, circle → other. See Tab. 1 for reference.

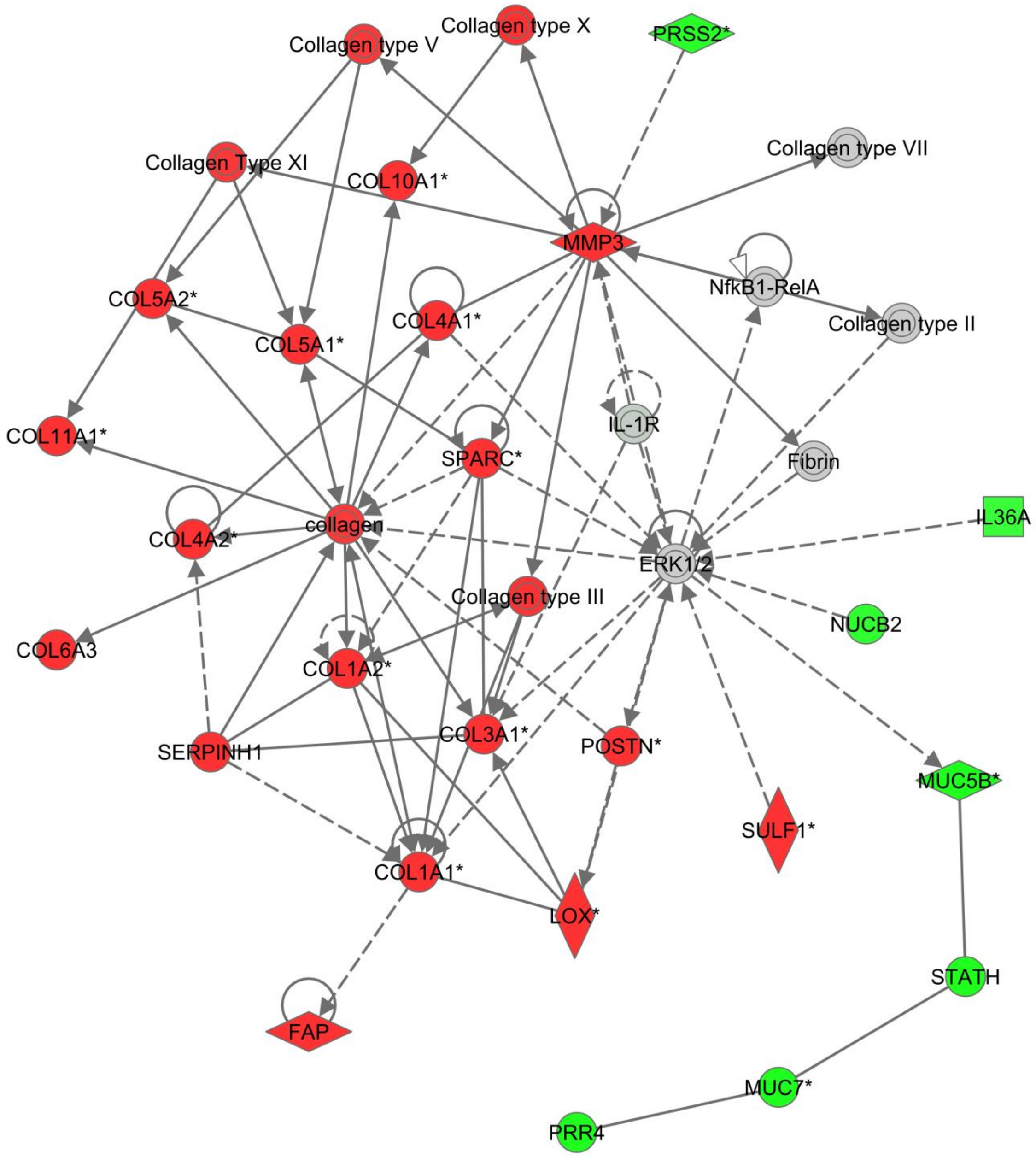

Fig. S13 – Top 02 network of differentially expressed genes in PT*vs.*N as identified by Ingenuity Pathway Analysis show strong association to connective tissue disorders, dermatological diseases and conditions, cellular assembly and organization, containing 24 focus molecules. For network analyses a threshold of $log_2FC > |1.5|$ with a p-value < 0.0001 was used, giving an overview of involved genes within the networks and their differential expression. Node colour indicates up-regulated (red), down-regulated (green) or not significantly changed according to threshold (grey) genes. Lines and arrows between nodes represent direct (solid lines) and indirect (dashed lines) interactions between molecules. All edges are supported by at least one reference from the literature or from canonical information stored in the Ingenuity Knowledge Base. Node shapes represent functional classes of gene products: square → cytokine, triangle → kinase, rectangle → nuclear receptor, concentric circle → group or complex, vertical diamond → enzyme,

horizontal diamond → peptidase, trapezium → transporter, vertical ellipse → transmembrane receptor horizontal ellipse→ transcription regulator, circle → other. See Tab. 1 for reference.

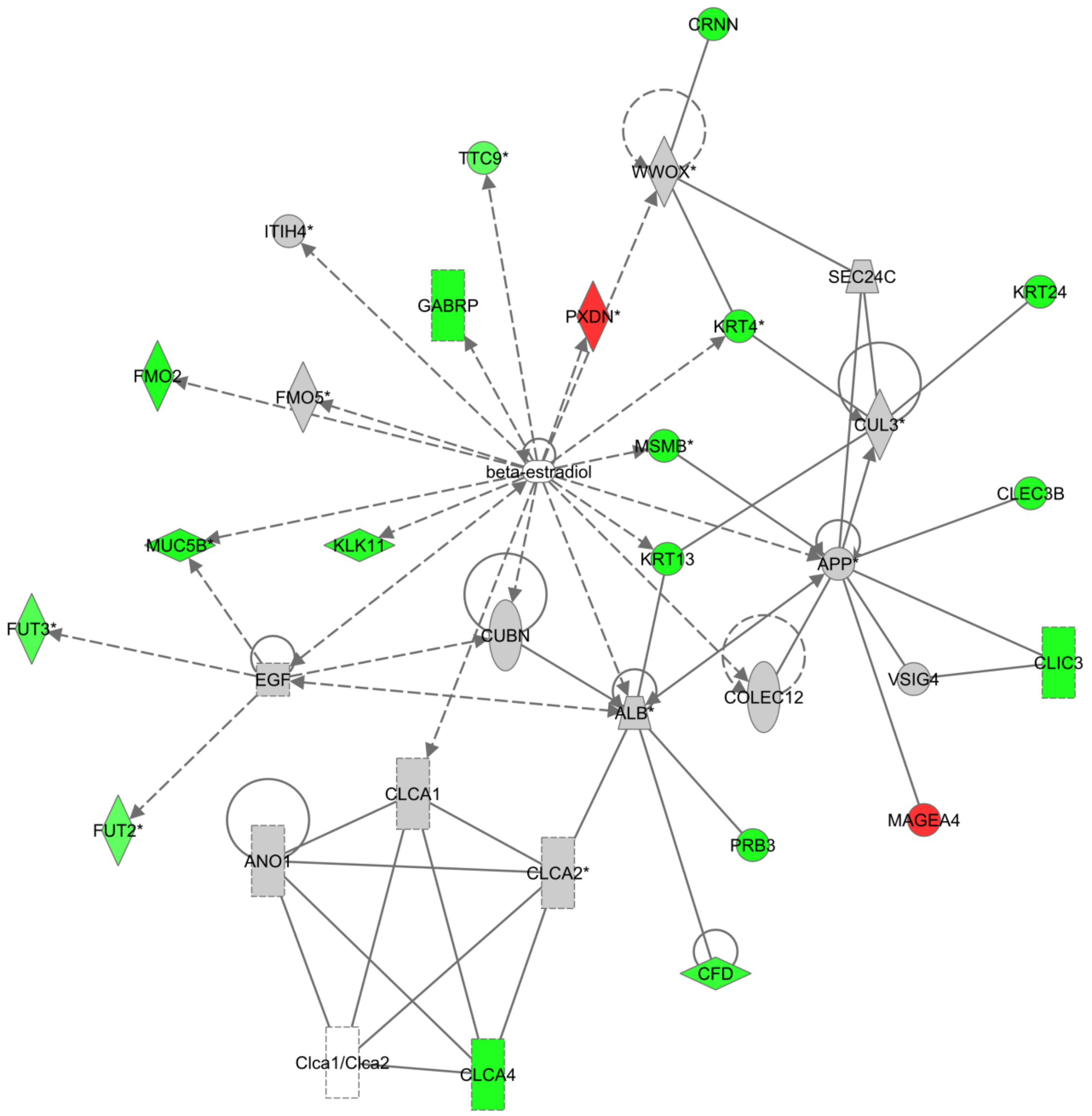

Fig. S14 – Top 03 network of differentially expressed genes in PT*vs.*N as identified by Ingenuity Pathway Analysis show strong association to molecular transport, cellular growth, proliferation and embryonic development, containing 19 focus molecules. For network analyses a threshold of $log_2FC > |1.5|$ with a p-value < 0.0001 was used, giving an overview of involved genes within the networks and their differential expression. Node colour indicates up-regulated (red), down-regulated (green) or not significantly changed according to threshold (grey) genes. Lines and arrows between nodes represent direct (solid lines) and indirect (dashed lines) interactions between molecules. All edges are supported by at least one reference from the literature or from canonical information stored in the Ingenuity Knowledge Base. Node shapes represent functional classes of gene products: square → cytokine, triangle → kinase, rectangle → nuclear receptor, concentric circle → group or complex, vertical diamond → enzyme, horizontal diamond → peptidase, trapezium → transporter, vertical ellipse → transmembrane receptor horizontal ellipse→ transcription regulator, circle → other. See Tab. 1 for reference.

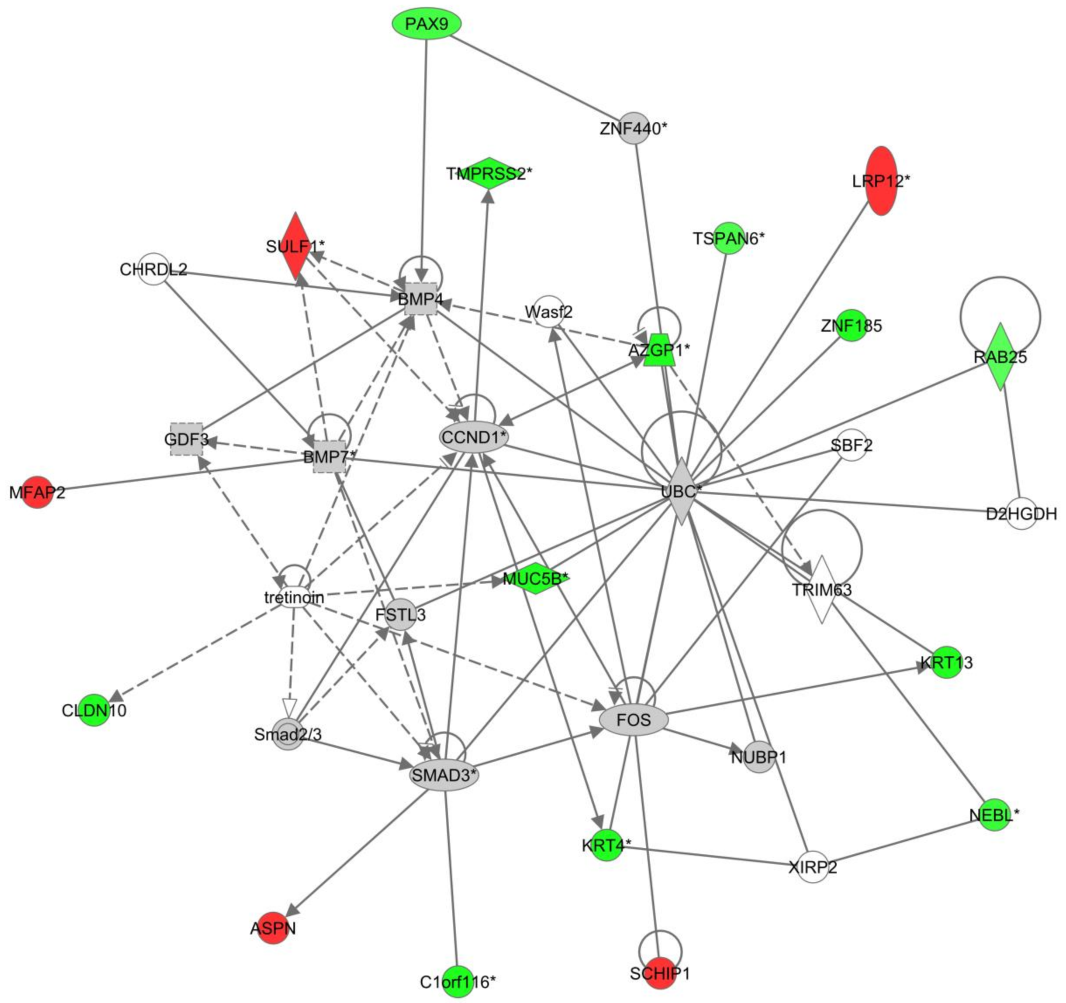

Fig. S15 – Top 04 network of differentially expressed genes in PT*vs.*N as identified by Ingenuity Pathway Analysis show strong association to embryonic developmet, organismal development and tissue development, containing 17 focus molecules. For network analyses a threshold of $\log_2 FC > |1.5|$ with a p-value < 0.0001 was used, giving an overview of involved genes within the networks and their differential expression. Node colour indicates up-regulated (red), down-regulated (green) or not significantly changed according to threshold (grey) genes. Lines and arrows between nodes represent direct (solid lines) and indirect (dashed lines) interactions between molecules. All edges are supported by at least one reference from the literature or from canonical information stored in the Ingenuity Knowledge Base. Node shapes represent functional classes of gene products: square → cytokine, triangle → kinase, rectangle → nuclear receptor, concentric circle → group or complex, vertical diamond → enzyme, horizontal diamond → peptidase, trapezium → transporter, vertical ellipse → transmembrane receptor horizontal ellipse→ transcription regulator, circle → other. See Tab. 1 for reference.

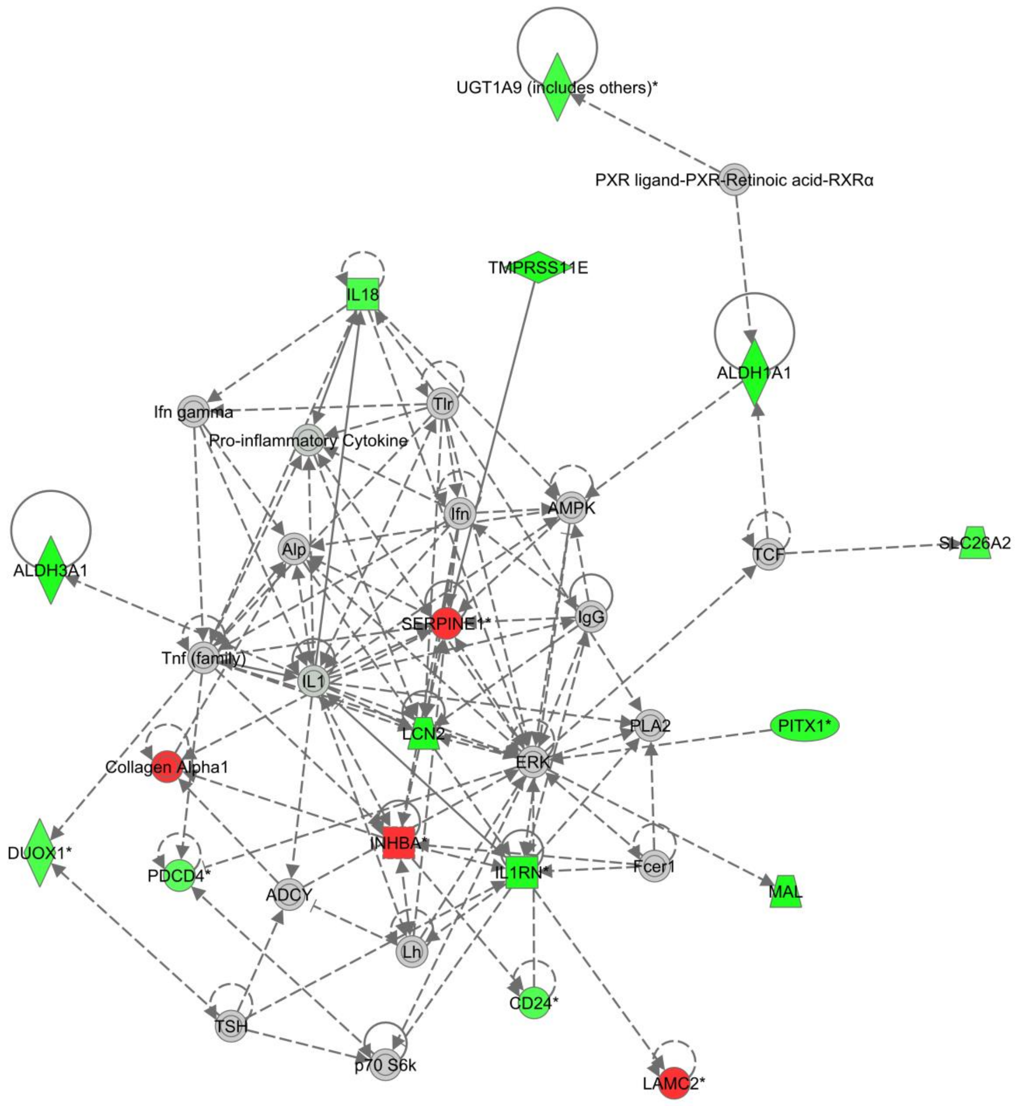

Fig. S16 – Top 05 network of differentially expressed genes in PT*vs.*N as identified by Ingenuity Pathway Analysis show strong association to inflammatory disease, organismal injury and abnormalities, as well as renal and urological disease, containing 16 focus molecules. For network analyses a threshold of $\log_2FC > |1.5|$ with a p-value < 0.0001 was used, giving an overview of involved genes within the networks and their differential expression. Node colour indicates up-regulated (red), down-regulated (green) or not significantly changed according to threshold (grey) genes. Lines and arrows between nodes represent direct (solid lines) and indirect (dashed lines) interactions between molecules. All edges are supported by at least one reference from the literature or from canonical information stored in the Ingenuity Knowledge Base. Node shapes represent functional classes of gene products: square → cytokine, triangle → kinase, rectangle → nuclear receptor, concentric circle → group or complex, vertical diamond → enzyme, horizontal diamond → peptidase, trapezium → transporter, vertical ellipse → transmembrane receptor horizontal ellipse→ transcription regulator, circle → other. See Tab. 1 for reference.

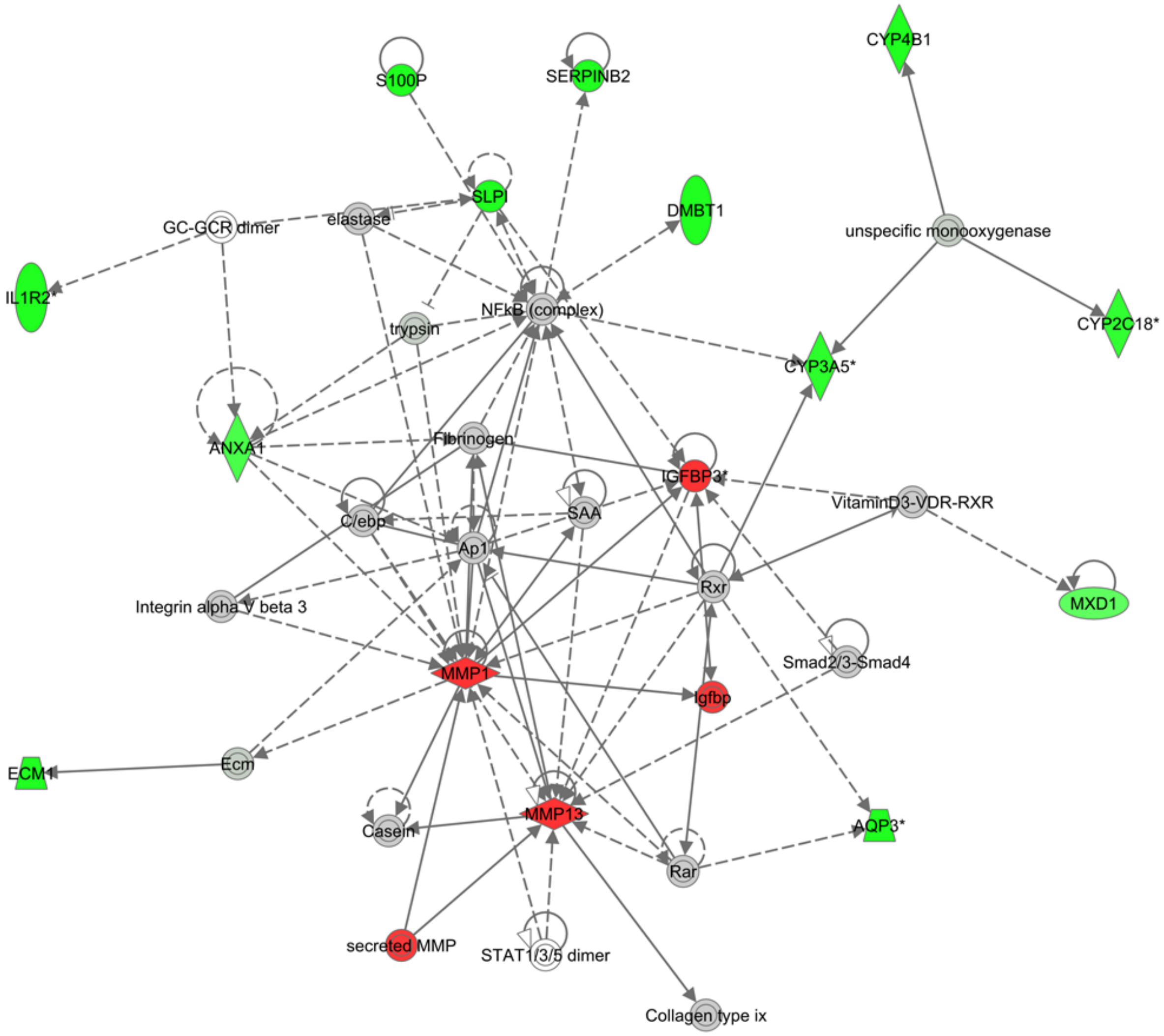

Fig. S17 – Top 06 network of differentially expressed genes in PT*vs.*N as identified by Ingenuity Pathway Analysis show strong association to dermatological diseases and conditions, inflammatory response and gastrointestinal disease, containing 15 focus molecules. For network analyses a threshold of $\log_2$FC >|1.5| with a p-value < 0.0001 was used, giving an overview of involved genes within the networks and their differential expression. Node colour indicates up-regulated (red), down-regulated (green) or not significantly changed according to threshold (grey) genes. Lines and arrows between nodes represent direct (solid lines) and indirect (dashed lines) interactions between molecules. All edges are supported by at least one reference from the literature or from canonical information stored in the Ingenuity Knowledge Base. Node shapes represent functional classes of gene products: square → cytokine, triangle → kinase, rectangle → nuclear receptor, concentric circle → group or complex, vertical diamond → enzyme, horizontal diamond → peptidase, trapezium → transporter, vertical ellipse → transmembrane receptor horizontal ellipse→ transcription regulator, circle → other. See Tab. 1 for reference.

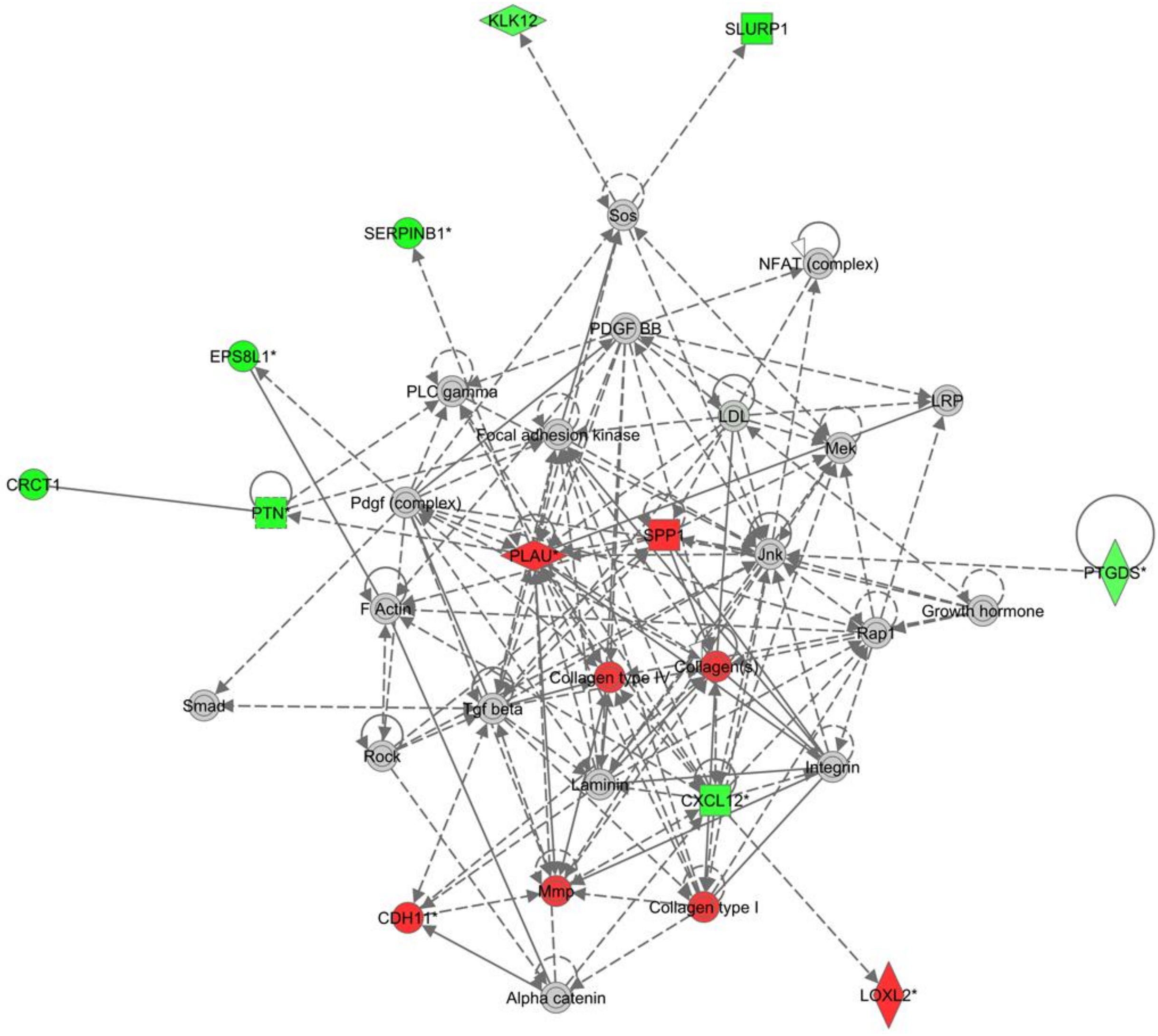

Fig. S18 – Top 07 network of differentially expressed genes in PT*vs.*N as identified by Ingenuity Pathway Analysis show strong association to organismal injury and abnormalities, as well as cellular movement, haematological system development and function, containing 12 focus molecules. For network analyses a threshold of $\log_2$FC >|1.5| with a p-value < 0.0001 was used, giving an overview of involved genes within the networks and their differential expression. Node colour indicates up-regulated (red), down-regulated (green) or not significantly changed according to threshold (grey) genes. Lines and arrows between nodes represent direct (solid lines) and indirect (dashed lines) interactions between molecules. All edges are supported by at least one reference from the literature or from canonical information stored in the Ingenuity Knowledge Base. Node shapes represent functional classes of gene products: square → cytokine, triangle → kinase, rectangle → nuclear receptor, concentric circle → group or complex, vertical diamond → enzyme, horizontal diamond → peptidase, trapezium → transporter, vertical ellipse → transmembrane receptor horizontal ellipse→ transcription regulator, circle → other. See Tab. 1 for reference.

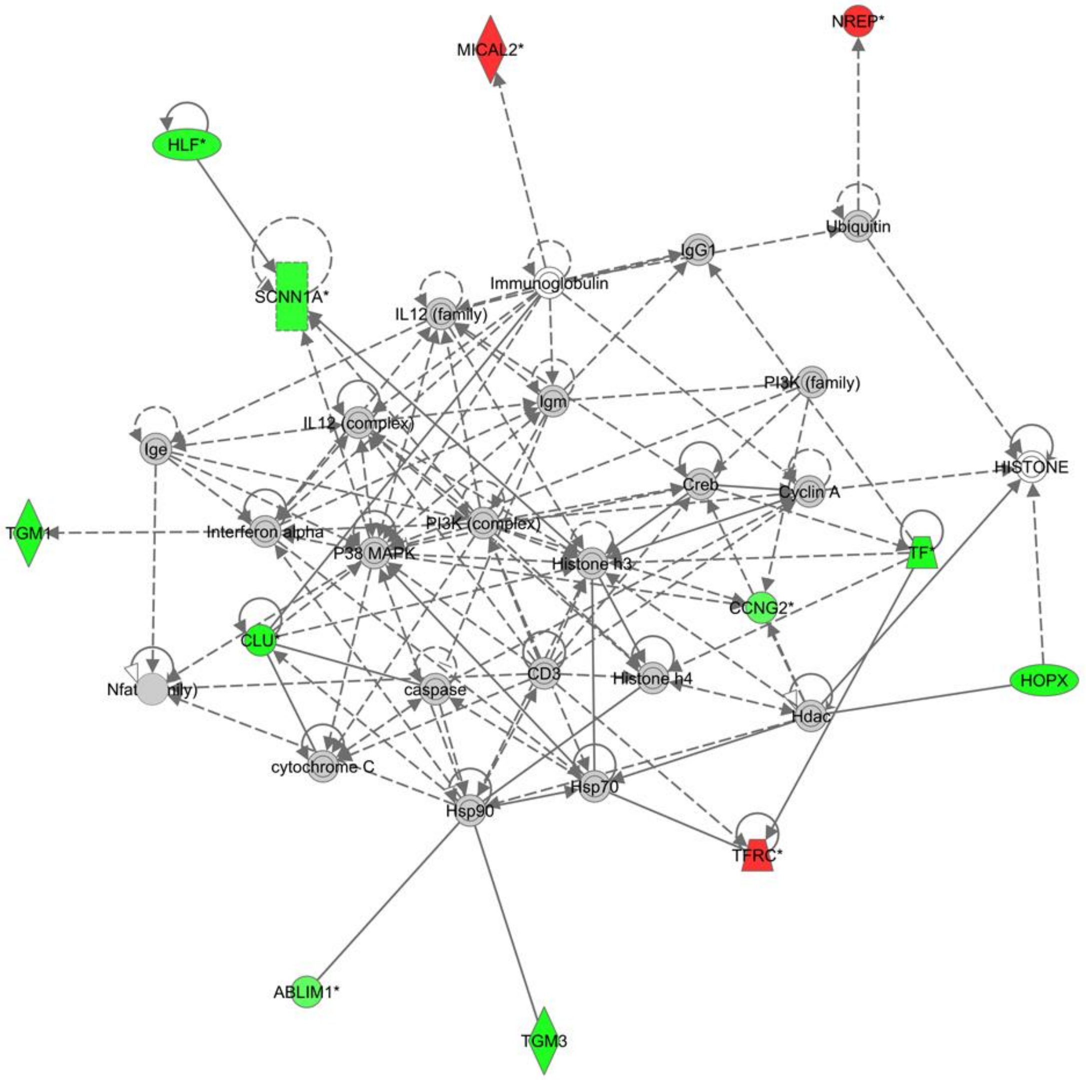

Fig. S19 – Top 08 network of differentially expressed genes in PT*vs.*N as identified by Ingenuity Pathway Analysis show strong association to cellular function and maintenance, small molecule biochemistry and amino acid metabolism, containing 12 focus molecules. For network analyses a threshold of $\log_2$FC >|1.5| with a p-value < 0.0001 was used, giving an overview of involved genes within the networks and their differential expression. Node colour indicates up-regulated (red), down-regulated (green) or not significantly changed according to threshold (grey) genes. Lines and arrows between nodes represent direct (solid lines) and indirect (dashed lines) interactions between molecules. All edges are supported by at least one reference from the literature or from canonical information stored in the Ingenuity Knowledge Base. Node shapes represent functional classes of gene products: square → cytokine, triangle → kinase, rectangle → nuclear receptor, concentric circle → group or complex, vertical diamond → enzyme, horizontal diamond → peptidase, trapezium → transporter, vertical ellipse → transmembrane receptor horizontal ellipse→ transcription regulator, circle → other. See Tab. 1 for reference.

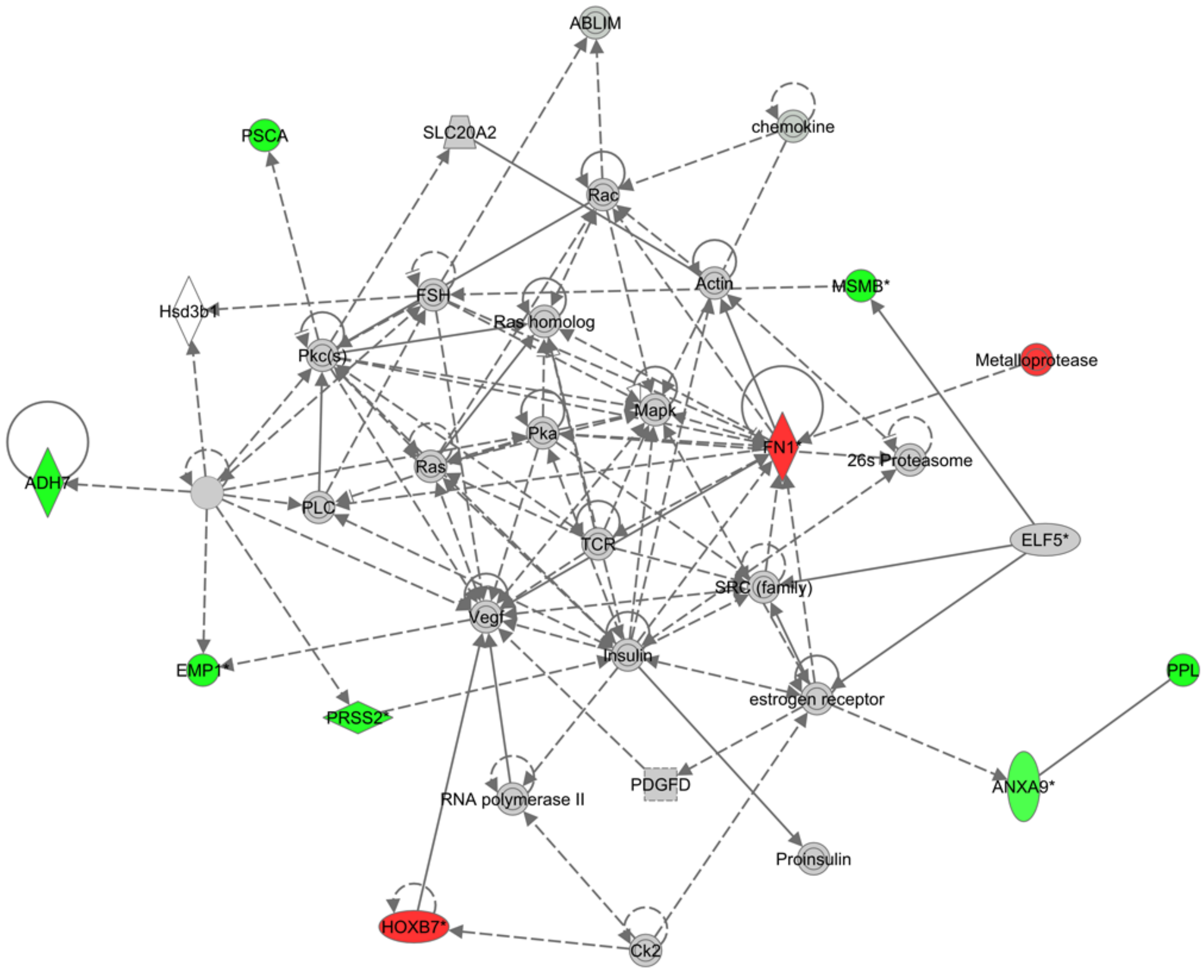

Fig. S20 – Top 09 network of differentially expressed genes in PT*vs.*N as identified by Ingenuity Pathway Analysis show strong association to cellular assembly and organization, as well as cellular function and maintenance, and cell death and survival, containing 9 focus molecules. For network analyses a threshold of $\log_2$FC >|1.5| with a p-value < 0.0001 was used, giving an overview of involved genes within the networks and their differential expression. Node colour indicates up-regulated (red), down-regulated (green) or not significantly changed according to threshold (grey) genes. Lines and arrows between nodes represent direct (solid lines) and indirect (dashed lines) interactions between molecules. All edges are supported by at least one reference from the literature or from canonical information stored in the Ingenuity Knowledge Base. Node shapes represent functional classes of gene products: square → cytokine, triangle → kinase, rectangle → nuclear receptor, concentric circle → group or complex, vertical diamond → enzyme, horizontal diamond → peptidase, trapezium → transporter, vertical ellipse → transmembrane receptor horizontal ellipse→ transcription regulator, circle → other. See Tab. 1 for reference.

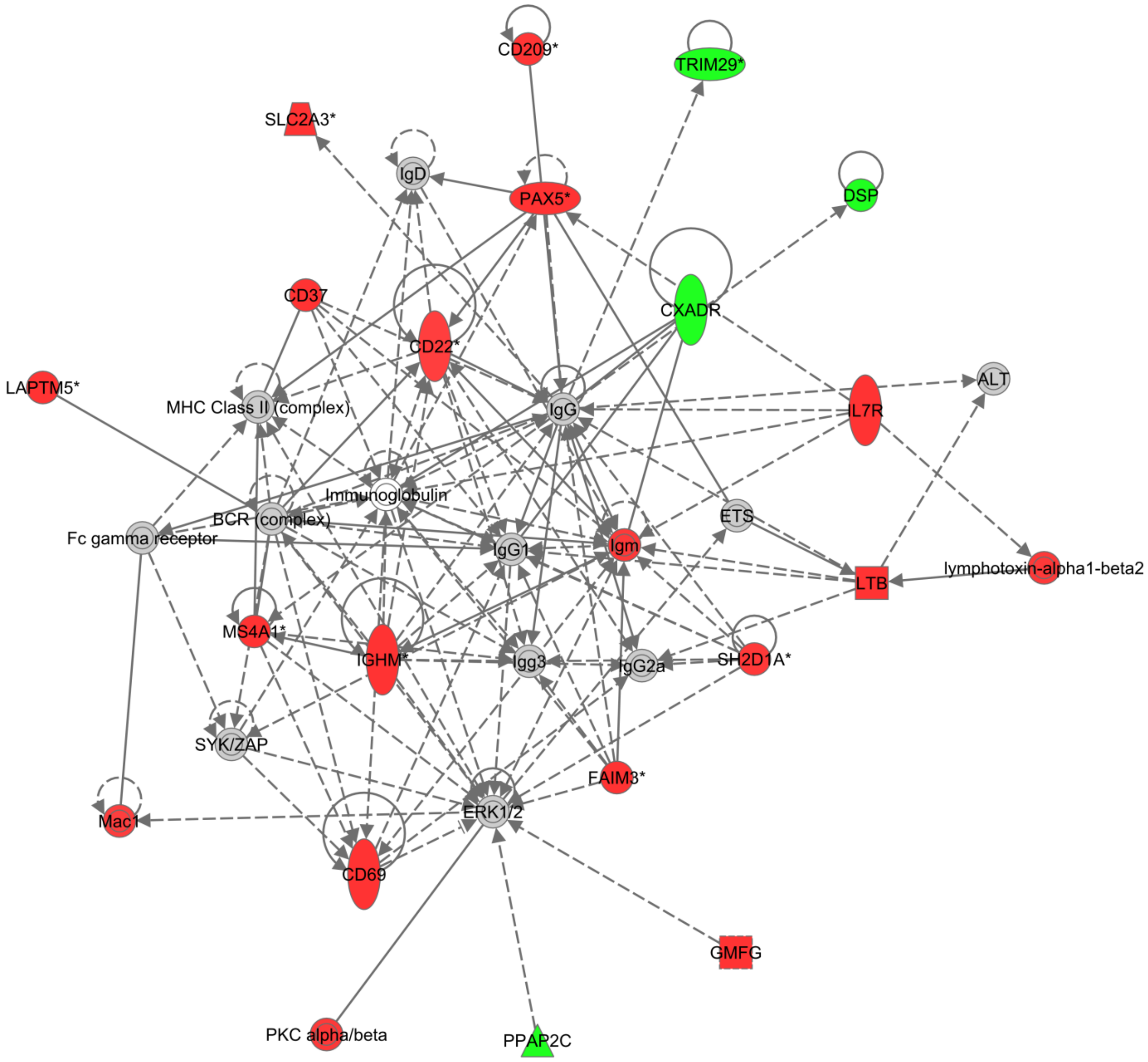

Fig S21 – Top 01 network of differentially expressed genes in M*vs.*PT as identified by Ingenuity Pathway Analysis show strong association to humoral immune response, protein synthesis, haematological system development and function, containing 18 focus molecules. For network analyses a threshold of $\log_2 FC > |1.5|$ with a p-value < 0.0001 was used, giving an overview of involved genes within the networks and their differential expression. Node colour indicates up-regulated (red), down-regulated (green) or not significantly changed according to threshold (grey) genes. Lines and arrows between nodes represent direct (solid lines) and indirect (dashed lines) interactions between molecules. All edges are supported by at least one reference from the literature or from canonical information stored in the Ingenuity Knowledge Base. Node shapes represent functional classes of gene products: square → cytokine, triangle → kinase, rectangle → nuclear receptor, concentric circle → group or complex, vertical diamond → enzyme, horizontal diamond → peptidase, trapezium → transporter, vertical ellipse → transmembrane receptor horizontal ellipse→ transcription regulator, circle → other. See Tab. 2 for reference.

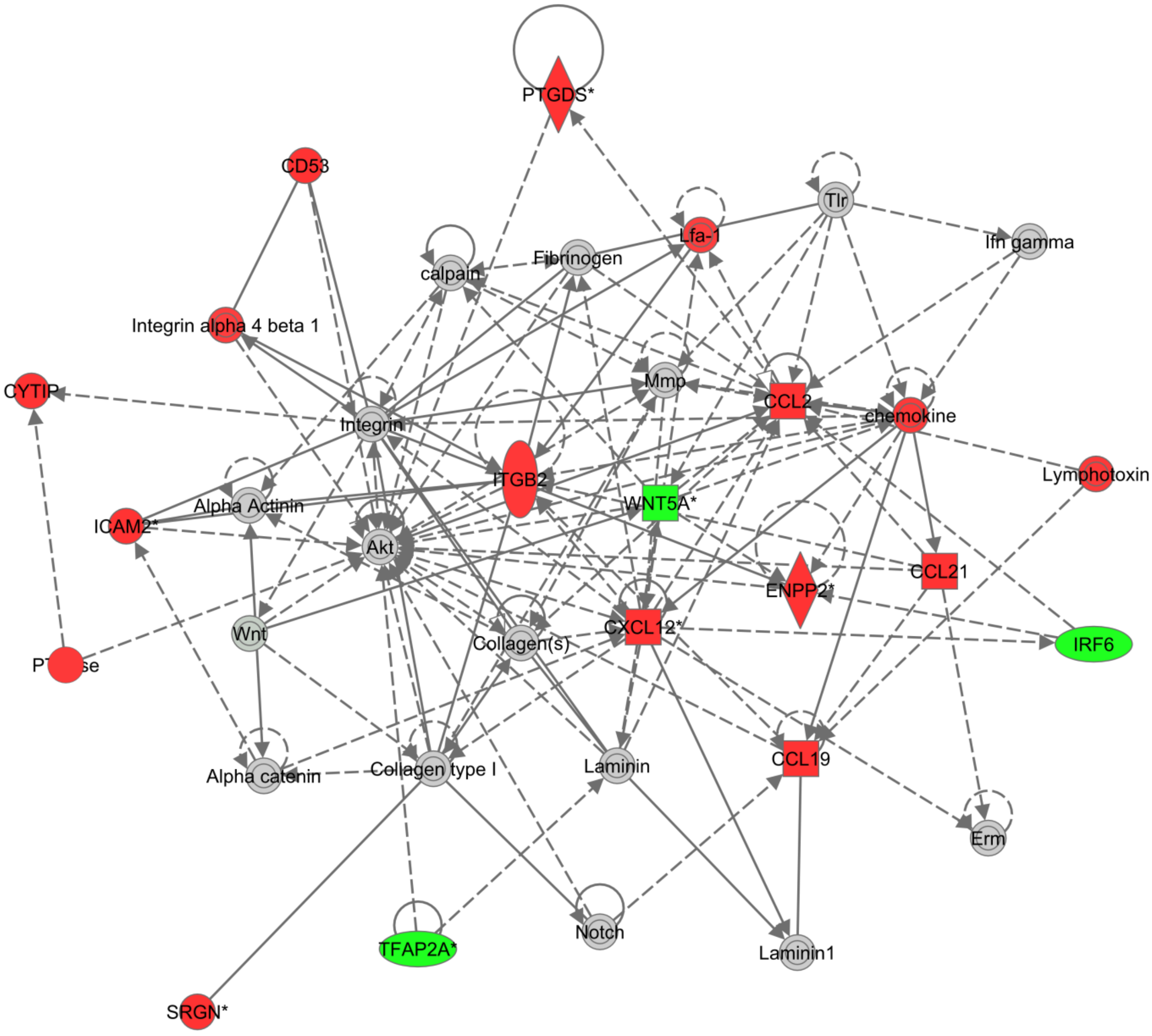

Fig S22 – Top 02 network of differentially expressed genes in M*vs.*PT as identified by Ingenuity Pathway Analysis show strong association to cellular movement, haematological system development and function, as well as immune cell trafficking, containing 14 focus molecules. For network analyses a threshold of $\log_2$FC >|1.5| with a p-value < 0.0001 was used, giving an overview of involved genes within the networks and their differential expression. Node colour indicates up-regulated (red), down-regulated (green) or not significantly changed according to threshold (grey) genes. Lines and arrows between nodes represent direct (solid lines) and indirect (dashed lines) interactions between molecules. All edges are supported by at least one reference from the literature or from canonical information stored in the Ingenuity Knowledge Base. Node shapes represent functional classes of gene products: square → cytokine, triangle → kinase, rectangle → nuclear receptor, concentric circle → group or complex, vertical diamond → enzyme, horizontal diamond → peptidase, trapezium → transporter, vertical ellipse → transmembrane receptor horizontal ellipse→ transcription regulator, circle → other. See Tab. 2 for reference.

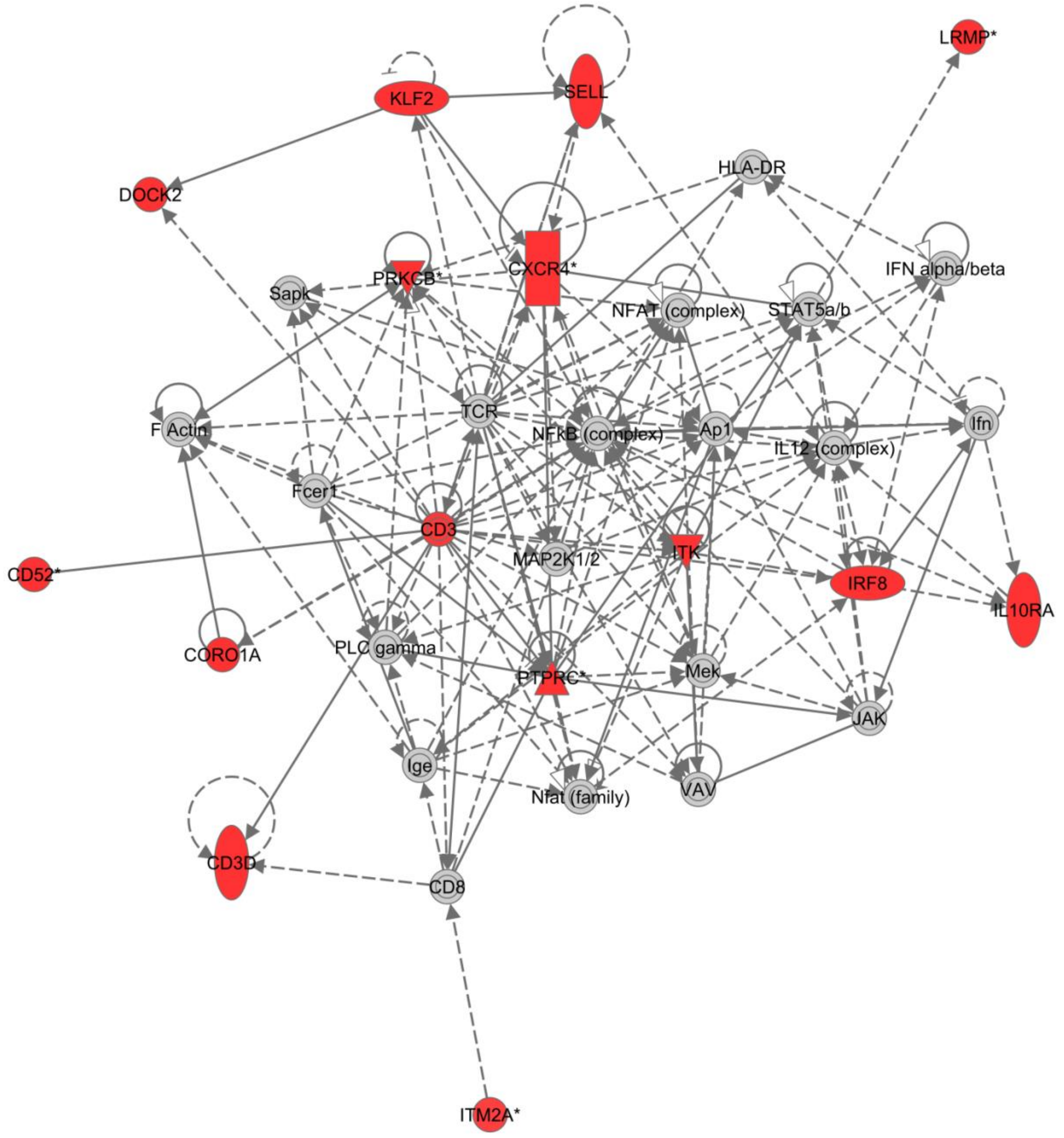

Fig. S23 – Top 03 network of differentially expressed genes in M*vs.*PT as identified by Ingenuity Pathway Analysis show strong association to cell-to-cell signalling and interaction, haematological system development and function, as well as tissue morphology, containing 14 focus molecules. For network analyses a threshold of log_2FC >|1.5| with a p-value < 0.0001 was used, giving an overview of involved genes within the networks and their differential expression. Node colour indicates up-regulated (red), down-regulated (green) or not significantly changed according to threshold (grey) genes. Lines and arrows between nodes represent direct (solid lines) and indirect (dashed lines) interactions between molecules. All edges are supported by at least one reference from the literature or from canonical information stored in the Ingenuity Knowledge Base. Node shapes represent functional classes of gene products: square → cytokine, triangle → kinase, rectangle → nuclear receptor, concentric circle → group or complex, vertical diamond → enzyme, horizontal diamond → peptidase, trapezium → transporter, vertical ellipse → transmembrane receptor horizontal ellipse→ transcription regulator, circle → other. See Tab. 2 for reference.

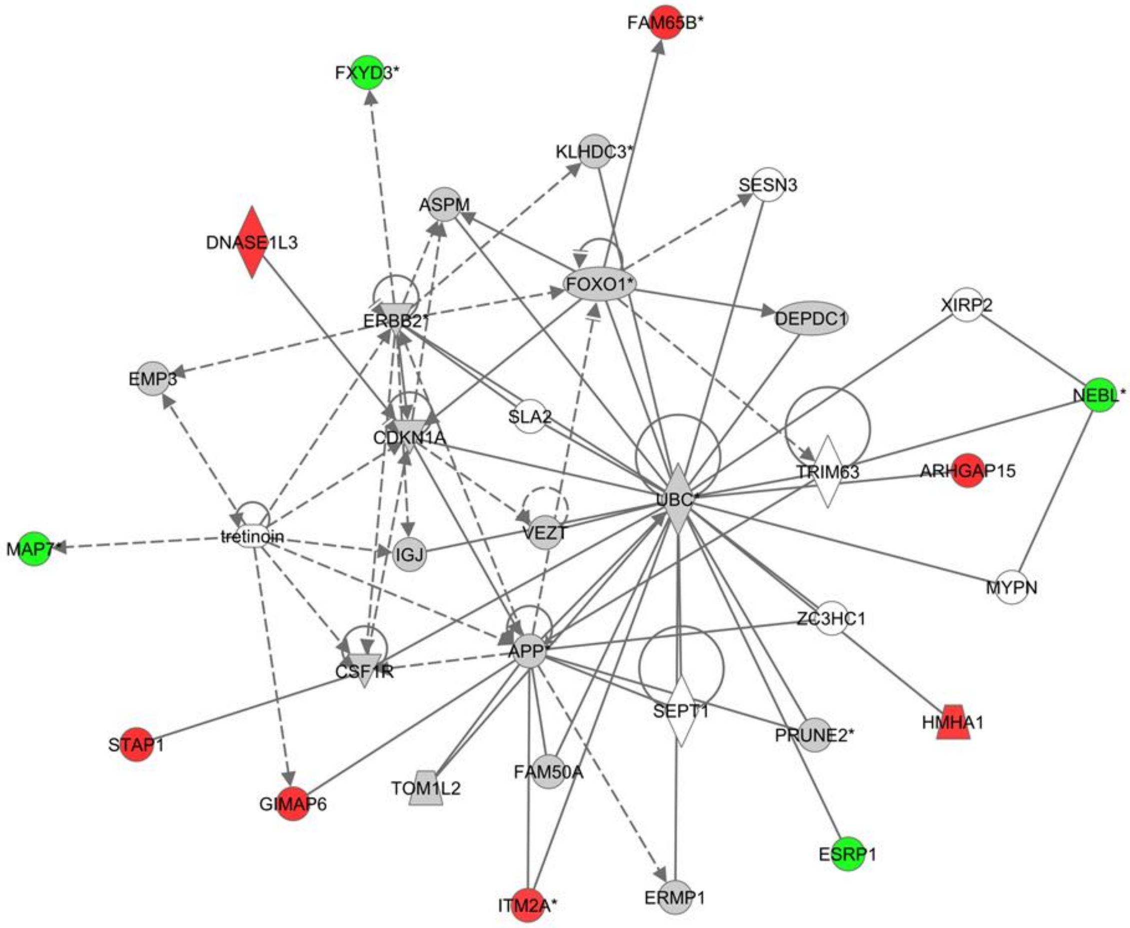

Fig. S24 – Top 04 network of differentially expressed genes in M*vs.*PT as identified by Ingenuity Pathway Analysis show strong association to cell cycle, cell morphology and cellular compromise, containing 11 focus molecules. For network analyses a threshold of $\log_2$FC >|1.5| with a p-value < 0.0001 was used, giving an overview of involved genes within the networks and their differential expression. Node colour indicates up-regulated (red), down-regulated (green) or not significantly changed according to threshold (grey) genes. Lines and arrows between nodes represent direct (solid lines) and indirect (dashed lines) interactions between molecules. All edges are supported by at least one reference from the literature or from canonical information stored in the Ingenuity Knowledge Base. Node shapes represent functional classes of gene products: square → cytokine, triangle → kinase, rectangle → nuclear receptor, concentric circle → group or complex, vertical diamond → enzyme, horizontal diamond → peptidase, trapezium → transporter, vertical ellipse → transmembrane receptor horizontal ellipse→ transcription regulator, circle → other. See Tab. 2 for reference.

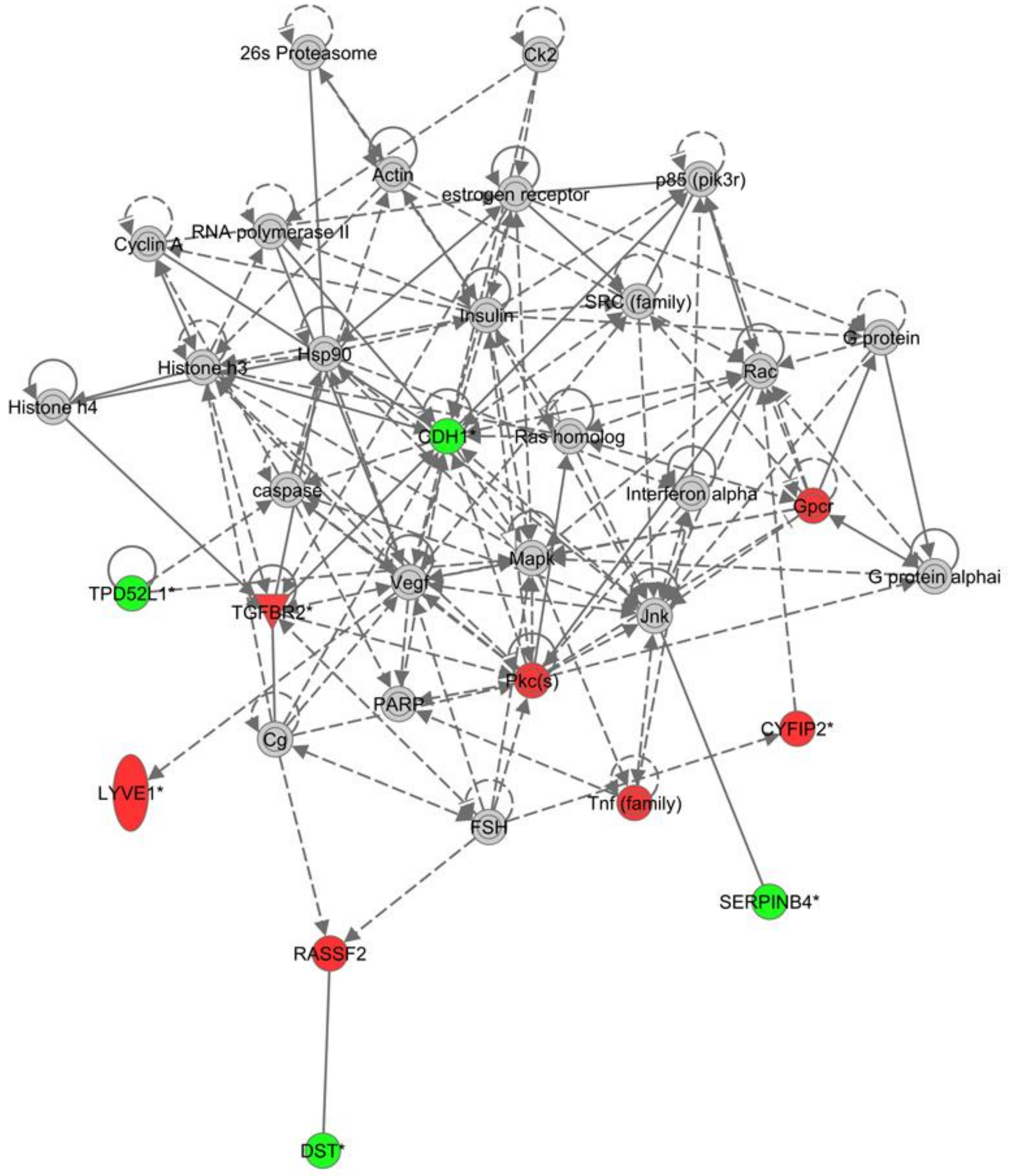

Fig. S25 – Top 05 network of differentially expressed genes in M*vs.*PT as identified by Ingenuity Pathway Analysis show strong association to cancer, dermatological diseases and conditions, as well as gastrointestinal disease, containing 8 focus molecules. For network analyses a threshold of $\log_2 FC > |1.5|$ with a p-value < 0.0001 was used, giving an overview of involved genes within the networks and their differential expression. Node colour indicates up-regulated (red), down-regulated (green) or not significantly changed according to threshold (grey) genes. Lines and arrows between nodes represent direct (solid lines) and indirect (dashed lines) interactions between molecules. All edges are supported by at least one reference from the literature or from canonical information stored in the Ingenuity Knowledge Base. Node shapes represent functional classes of gene products: square → cytokine, triangle → kinase, rectangle → nuclear receptor, concentric circle → group or complex, vertical diamond → enzyme, horizontal diamond → peptidase, trapezium → transporter, vertical ellipse → transmembrane receptor horizontal ellipse→ transcription regulator, circle → other. See Tab. 2 for reference.

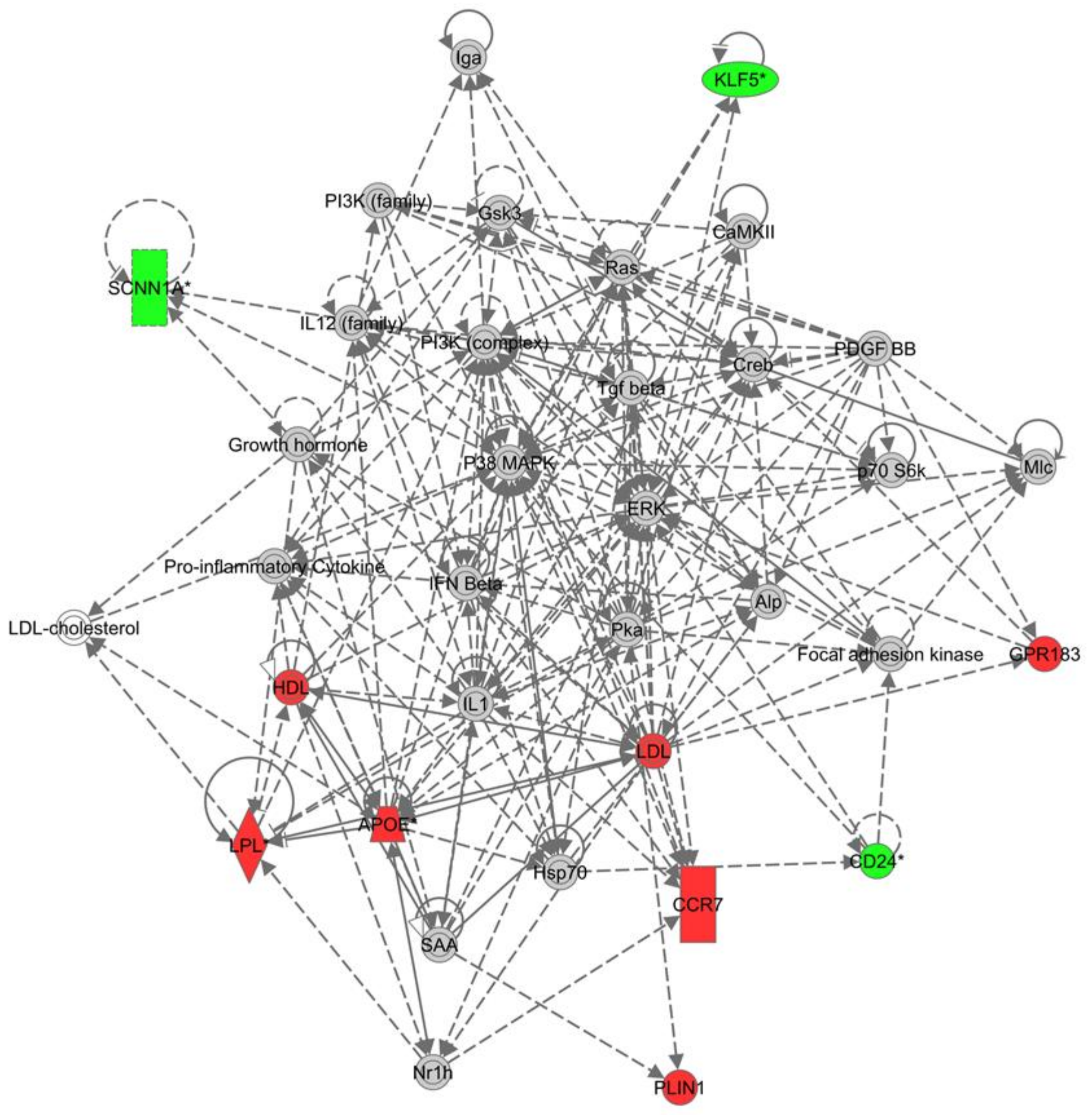

Fig. S26 – Top 06 network of differentially expressed genes in M*vs.*PT as identified by Ingenuity Pathway Analysis show strong association to lipid metabolism, small molecule biochemistry and cardiovascular disease, containing 8 focus molecules. For network analyses a threshold of $\log_2$FC >|1.5| with a p-value < 0.0001 was used, giving an overview of involved genes within the networks and their differential expression. Node colour indicates up-regulated (red), down-regulated (green) or not significantly changed according to threshold (grey) genes. Lines and arrows between nodes represent direct (solid lines) and indirect (dashed lines) interactions between molecules. All edges are supported by at least one reference from the literature or from canonical information stored in the Ingenuity Knowledge Base. Node shapes represent functional classes of gene products: square → cytokine, triangle → kinase, rectangle → nuclear receptor, concentric circle → group or complex, vertical diamond → enzyme, horizontal diamond → peptidase, trapezium → transporter, vertical ellipse → transmembrane receptor horizontal ellipse→ transcription regulator, circle → other. See Tab. 2 for reference.

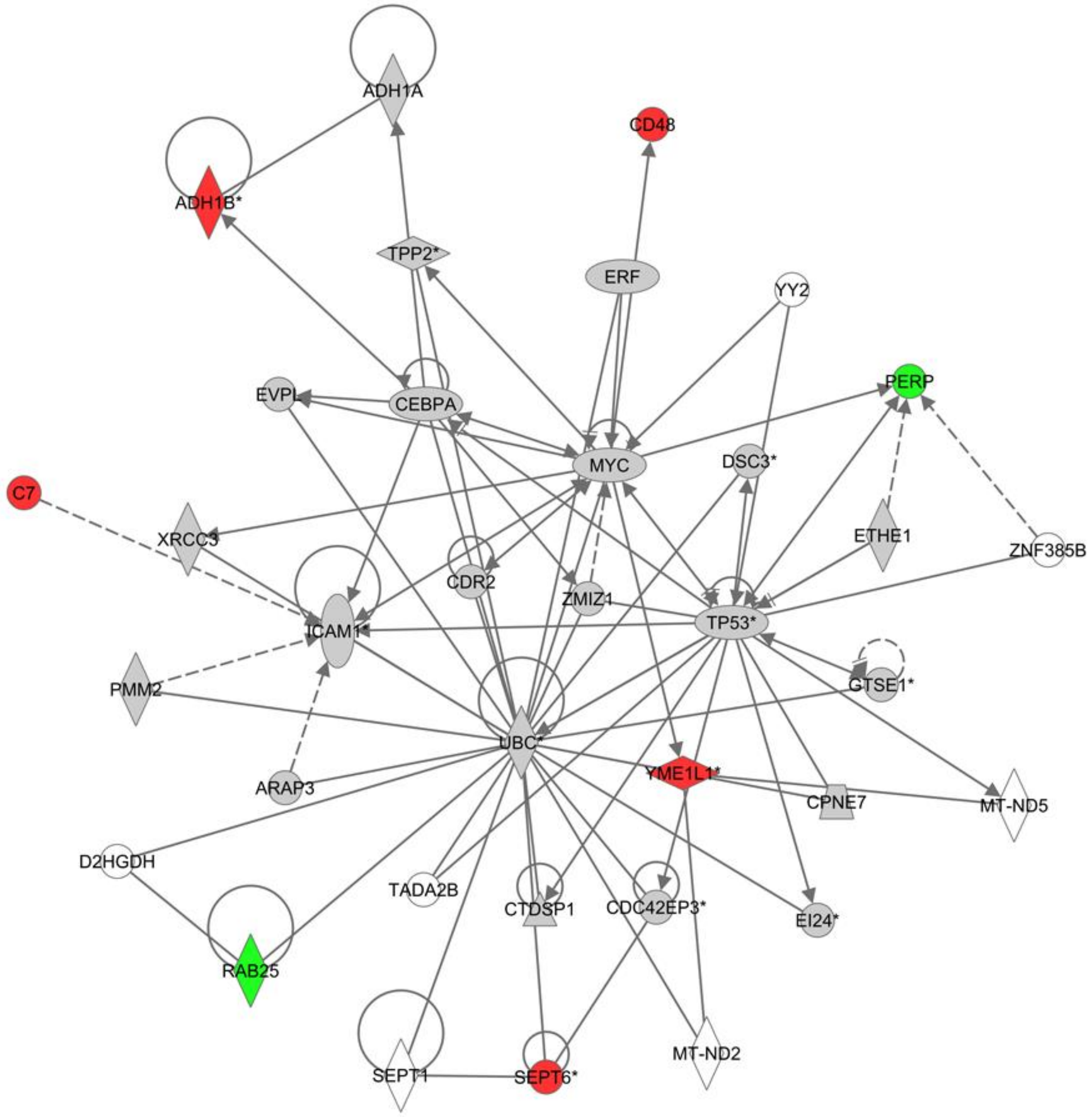

Fig. S27 – Top 07 network of differentially expressed genes in M*vs.*PT as identified by Ingenuity Pathway Analysis show strong association to cancer, endocrine system disorders and developmental disorder, containing 7 focus molecules. For network analyses a threshold of $log_2FC > |1.5|$ with a p-value < 0.0001 was used, giving an overview of involved genes within the networks and their differential expression. Node colour indicates up-regulated (red), down-regulated (green) or not significantly changed according to threshold (grey) genes. Lines and arrows between nodes represent direct (solid lines) and indirect (dashed lines) interactions between molecules. All edges are supported by at least one reference from the literature or from canonical information stored in the Ingenuity Knowledge Base. Node shapes represent functional classes of gene products: square → cytokine, triangle → kinase, rectangle → nuclear receptor, concentric circle → group or complex, vertical diamond → enzyme, horizontal diamond → peptidase, trapezium → transporter, vertical ellipse → transmembrane receptor horizontal ellipse→ transcription regulator, circle → other. See Tab. 2 for reference.

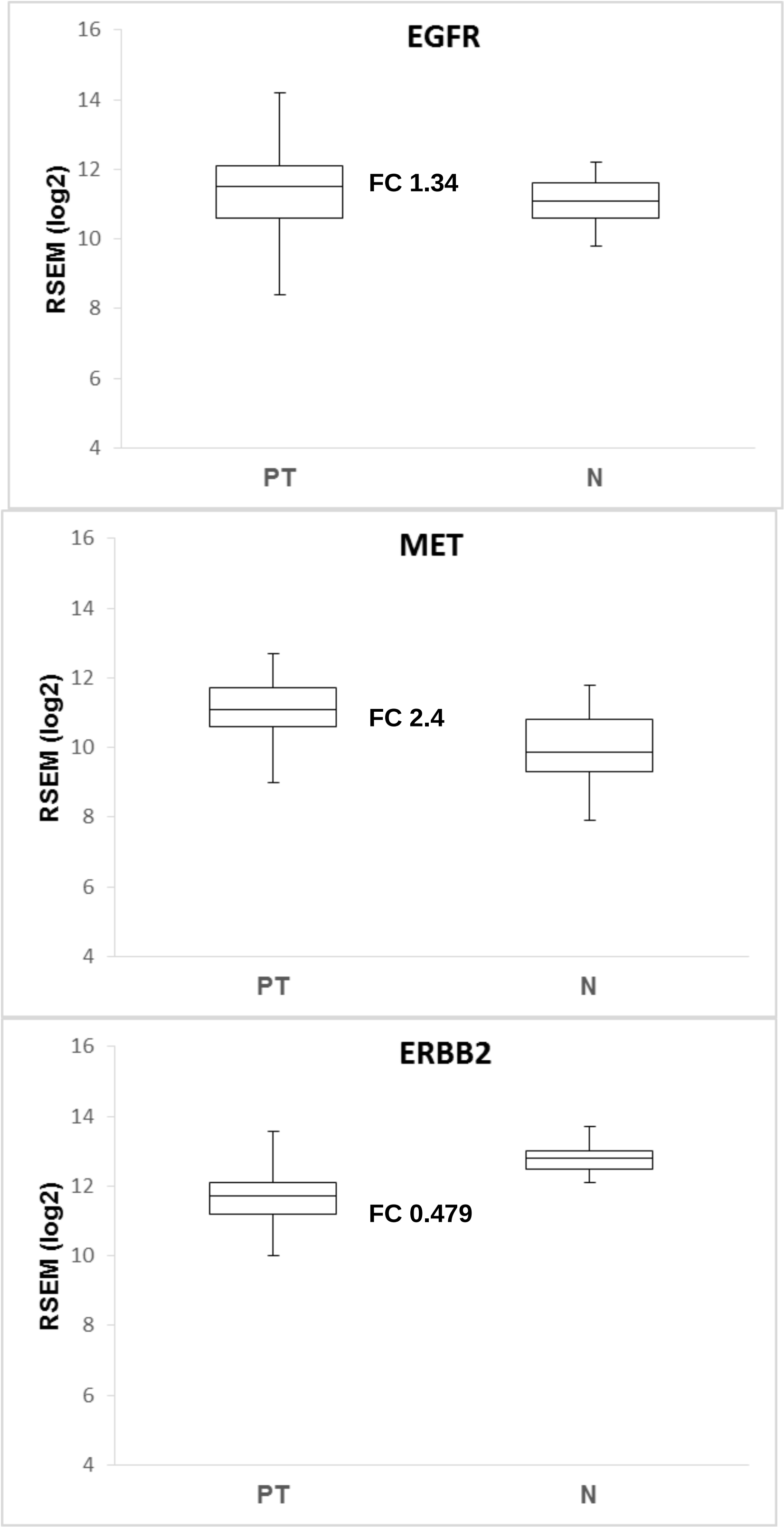

Fig. S28: Differential expression of selected genes based on TCGA HNSCC RNA-Seq Data[35]. Differential box plot of RSEM log2 values are shown for 522 HNSCC tumour samples (PT) compared to 44 normal samples (N). FC: fold change as indicated.

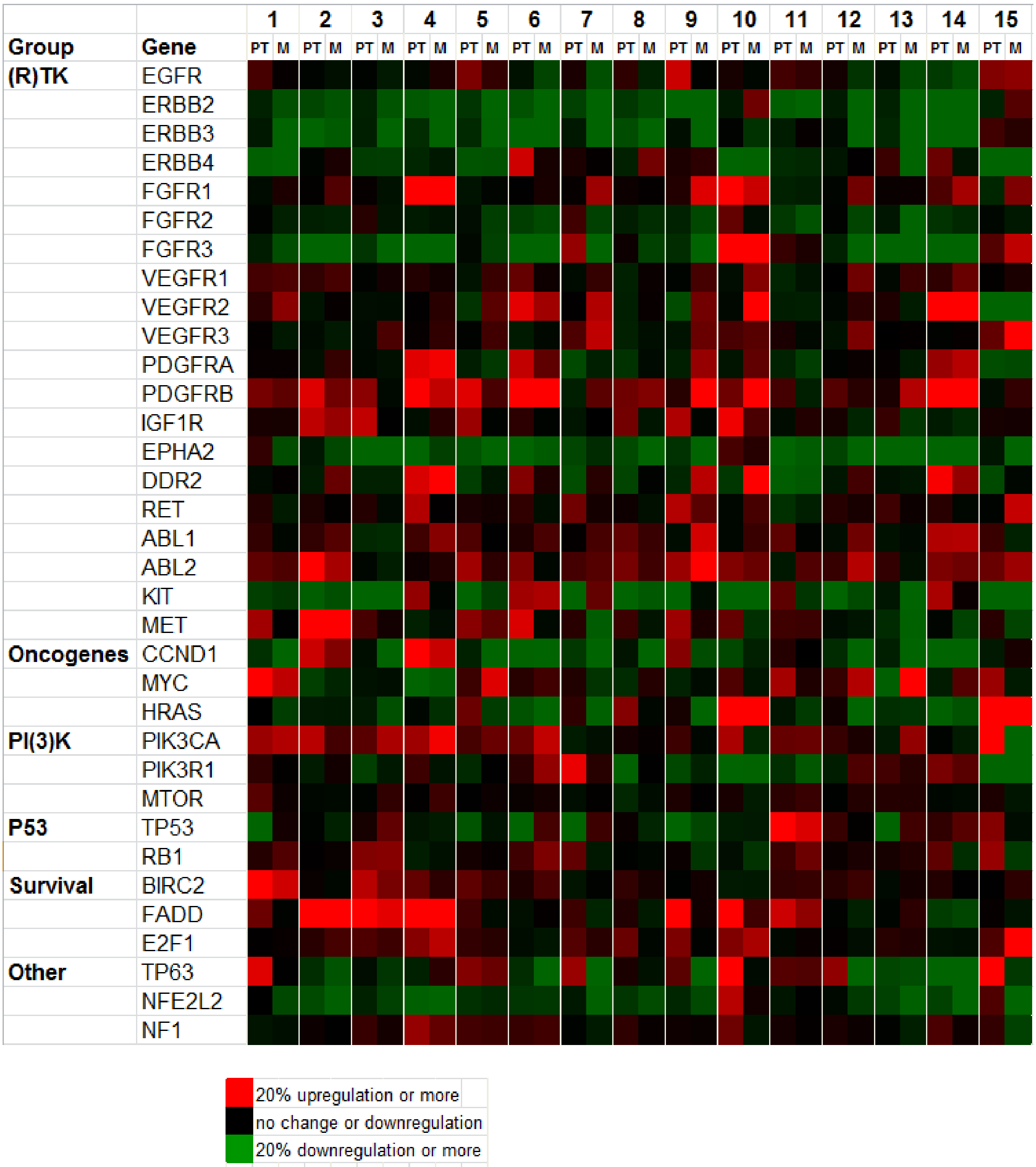

Fig. S29: Comparison of Receptor Tyrosine Kinase and Oncogene Expression of all patients. Differential expression values for selected genes were colour coded (bright red for an overexpression of ≥20%, bright green for a downregulation of ≥20%). In a ratio to their corresponding normal tissue mucosa, primary tumour values (PT) and metastasis values (M) are listed for each patient 1-15.

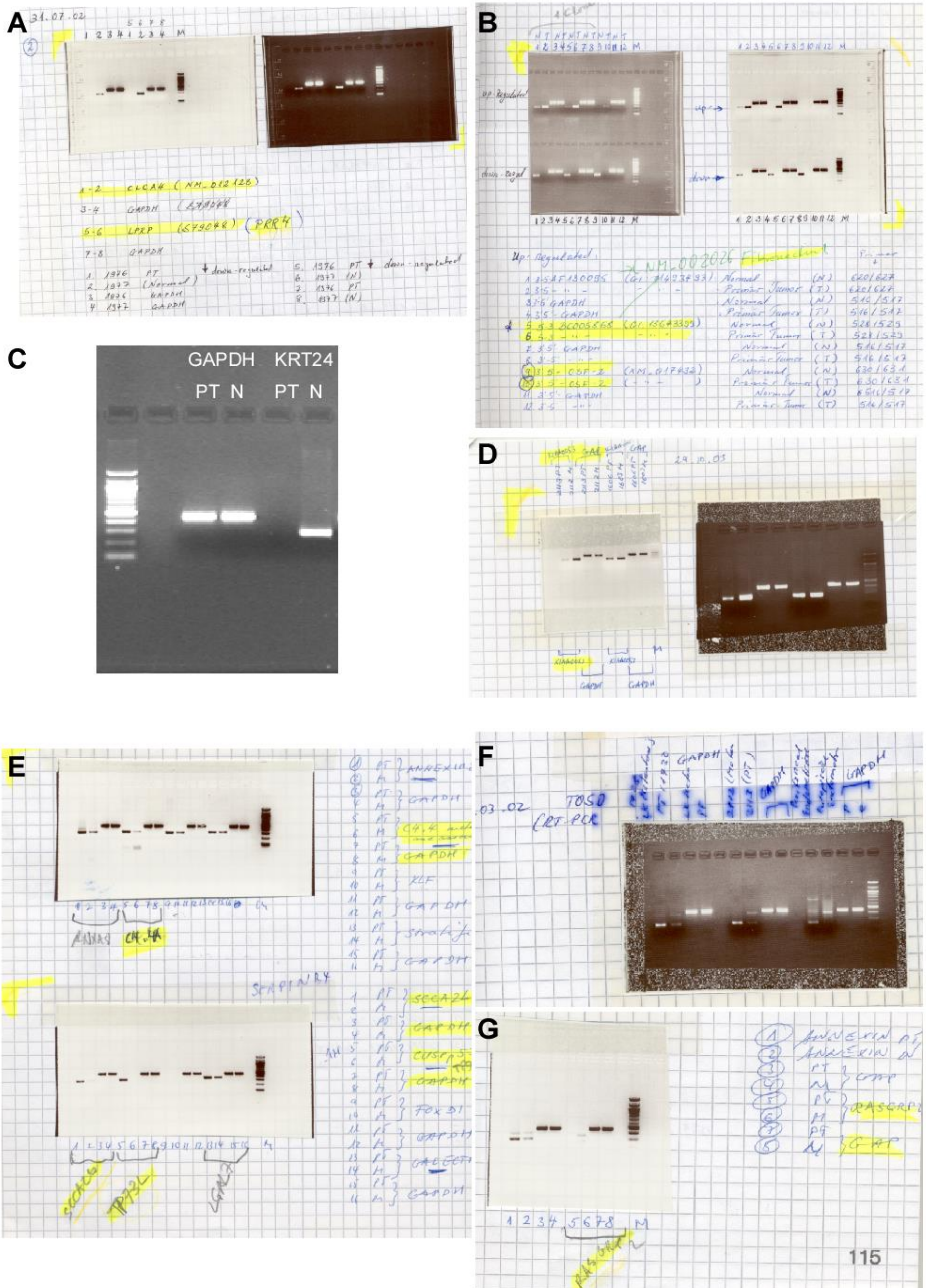
A
B
C
GAPDH
KRT24
PT N
PT N
D
E
F
G

Fig. S30: Verification of gene expression data obtained by Microarray Analysis via *Affymetrix U133A GeneChip* arrays for selected genes using RT-PCR. Full-length gels referring to Supplementary Figure S1. **A)** CLCA4 and PRR4 (LPRP), **B)** FN1 (BC005858) and OSF-2., **C)** KRT24 in PT*vs.*N. **D)** ARHGAP25 (KIAA0053), **E)** LYPD3 (C4.4A), SERPINB4 (SCC-A2), and TP73L (CUSP), **F)** FCMR (TOSO), and **G)** RASGRP2 in M*vs.*PT.
